# Controllable Enzyme Immobilization via Simple and Quantitative Adsorption of Dendronized Polymer–Enzyme Conjugates Inside a Silica Monolith for Enzymatic Flow-Through Reactor Applications

**DOI:** 10.1021/acsomega.2c02815

**Published:** 2022-07-21

**Authors:** Nicolas Ghéczy, Weina Xu, Katarzyna Szymańska, Andrzej B. Jarzębski, Peter Walde

**Affiliations:** † Laboratory for Multifunctional Materials, Department of Materials, ETH Zürich, Vladimir-Prelog-Weg 5, Zürich 8093, Switzerland; ‡ Department of Chemical Engineering and Process Design, Silesian University of Technology, Księdza Marcina Strzody 7, Gliwice 44-100, Poland; ⊥ Institute of Chemical Engineering, Polish Academy of Sciences, Baltycka 5, Gliwice 44-100, Poland

## Abstract

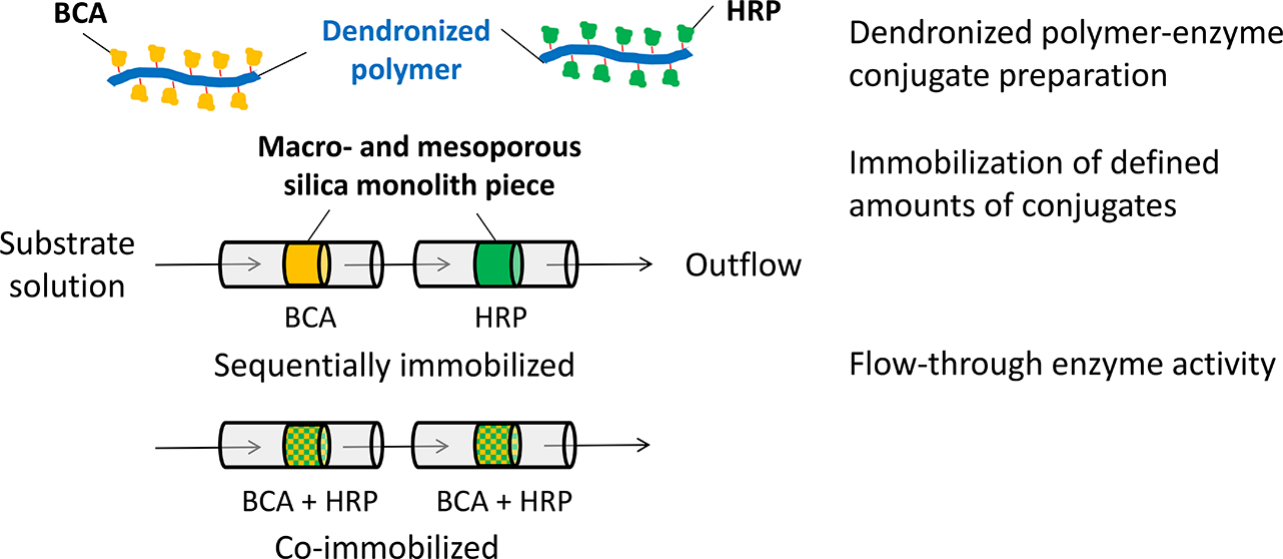

Although many different methods are known for the immobilization of enzymes on solid supports for use in flow-through applications as enzyme reactors, the reproducible immobilization of predetermined amounts of catalytically active enzyme molecules remains challenging. This challenge was tackled using a macro- and mesoporous silica monolith as a support and dendronized polymer–enzyme conjugates. The conjugates were first prepared in an aqueous solution by covalently linking enzyme molecules and either horseradish peroxidase (HRP) or bovine carbonic anhydrase (BCA) along the chains of a water-soluble second-generation dendronized polymer using an established procedure. The obtained conjugates are stable biohybrid structures in which the linking unit between the dendronized polymer and each enzyme molecule is a bisaryl hydrazone (BAH) bond. Quantitative and reproducible enzyme immobilization inside the monolith is possible by simply adding a defined volume of a conjugate solution of a defined enzyme concentration to a dry monolith piece of the desired size. In that way, (i) the entire volume of the conjugate solution is taken up by the monolith piece due to capillary forces and (ii) all conjugates of the added conjugate solution remain stably adsorbed (immobilized) noncovalently without detectable leakage from the monolith piece. The observed flow-through activity of the resulting enzyme reactors was directly proportional to the amount of conjugate used for the reactor preparation. With conjugate solutions consisting of defined amounts of both types of conjugates, the controlled coimmobilization of the two enzymes, namely, BCA and HRP, was shown to be possible in a simple way. Different stability tests of the enzyme reactors were carried out. Finally, the enzyme reactors were applied to the catalysis of a two-enzyme cascade reaction in two types of enzymatic flow-through reactor systems with either coimmobilized or sequentially immobilized BCA and HRP. Depending on the composition of the substrate solution that was pumped through the two types of enzyme reactor systems, the coimmobilized enzymes performed significantly better than the sequentially immobilized ones. This difference, however, is not due to a molecular proximity effect with regard to the enzymes but rather originates from the kinetic features of the cascade reaction used. Overall, the method developed for the controllable and reproducible immobilization of enzymes in the macro- and mesoporous silica monolith offers many possibilities for systematic investigations of immobilized enzymes in enzymatic flow-through reactors, potentially for any type of enzyme.

## 1 Introduction

Over the last few decades, a number of different methods for the immobilization of enzymes on solid surfaces have been developed, as summarized in many review articles.^1−27^ A recent focus is on the immobilization of enzymes for flow-through applications.^8,28−31^ Conceptually, the methods for enzyme immobilization on silica surfaces can be grouped into at least three categories: (i) covalent binding to a silica surface using organic linker moieties and a chemical modification of the silica surface,^28,32^ (ii) noncovalent adsorption on either neat silica or surface-modified silica,^27,32^ and (iii) entrapment in the pores of porous silica materials.^14,21,32^ For the methods based on noncovalent enzyme adsorption, three approaches are relevant for comparison to the work presented: (i) layer-by-layer deposition using a charged polymer that has an opposite charge to the overall charge of the enzyme at the pH applied,^33,34^ (ii) the use of recombinant enzymes carrying His-tags to bind to a silica surface that is surface-functionalized to allow the efficient binding of His,^24^ and (iii) the use of recombinant chimeric enzymes containing a polycationic protein module (an arginine-rich mini protein) that binds to unmodified, anionic silica surfaces (“fusion protein approach”).^12,13,24^ The methodology used in this work is somewhat related to the fusion protein approach, although the use of recombinant enzymes is not required. In our work, the enzyme of interest is immobilized noncovalently on unmodified silica surfaces after several enzyme molecules are first covalently bound to polymer molecules in an aqueous solution.^35−41^

**Figure 1:**
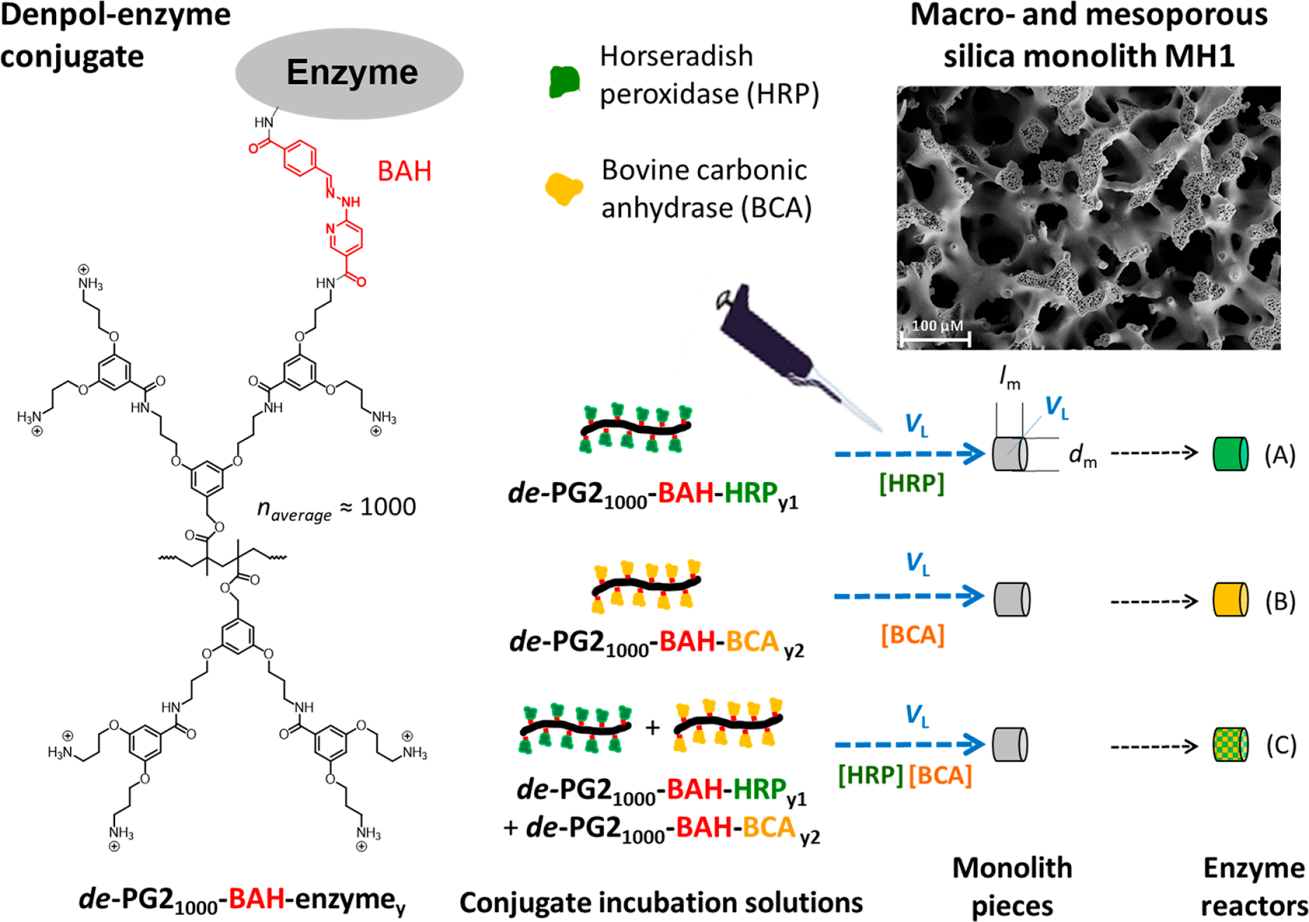
Chemical structure of the synthesized denpol–enzyme conjugates, *de*-PG2_1000_-BAH-enzyme_*y*_, and overview of the three types of enzyme reactors prepared. Using the enzymes HRP and BCA, defined volumes of aqueous conjugate incubation solutions (*V*_L_) containing either defined amounts of one of the two conjugates (at defined concentrations of active enzymes, [HRP] or [BCA]) or a defined mixture of the two conjugates were added to pieces of the macro- and mesoporous silica monolith MH1 of defined length (*l*_m_, usually 5 mm) and diameter (*d*_m_ ≈ 4 mm). This yielded enzyme reactors containing either (A and B) defined amounts of one of the two enzymes or (C) defined amounts of both enzymes. The enzyme molecules are attached along the chain of the water-soluble dendronized polymer *de*-PG2_1000_, with an average of 1000 r.u., via bisaryl hydrazone (BAH) bonds (in red). HRP is indicated in green (*y*_1_ = 20 or 40), and BCA is indicated in dark orange (*y*_2_ = 54 or 89). A scanning electron microscopy image of MH1 is shown to illustrate the ≈20–30 μm large macropores present (the length of the bar represents 100 μm).

The polymer used in all our previous work was a fully synthetic second-generation dendronized polymer (denpol) composed of four peripheral primary amines in each repeating unit (r.u.) (see Figure 1).^39,42,43^ This denpol is abbreviated as *de*-PG2_*x*_. The subscript *x* refers to the average number of r.u.’s, for example, *x* = 1000; PG2 stands for “dendronized polymer of second generation”; and *de* indicates that the denpol is deprotected, that is, the amine’s protecting group used during the chemical synthesis of the denpol (*tert*-butyloxycarbonyl) has been removed. Under conditions of at least the partial protonation of the many amino groups, the denpol *de*-PG2_*x*_ is water-soluble (at pH values below about 8).^37^ Enzyme molecules can be attached covalently along the denpol chain under mild conditions in an aqueous solution using the bisaryl hydrazone (BAH) linking chemistry.^39,44^ BAH bonds (Figure 1) form between enzyme molecules that are modified on their surface with S-4FB (*N*-succinimidyl 4-formyl benzoate) through reaction with the ε-amino groups of surface-exposed, reactive lysine residues and those amino groups of *de*-PG2_*x*_ modified with S-HyNic (*N*-succinimidyl 6-hydrazinonicotionate acetone hydrazone).^39,44^ The denpol–enzyme conjugates obtained in this way are biohybrid structures that contain several covalently bound enzyme molecules (*y*) along the denpol chain, abbreviated as *de*-PG2_*x*_-BAH-enzyme_*y*_, for example, *de*-PG2_2000_-BAH-proK_140_ (proK = *Engyodontium album* proteinase K).^38^

After demonstrating the successful immobilization of various enzymes on different silica surfaces through noncovalent adsorption of preprepared denpol–enzyme conjugates,^35−38,40,41^ we explored in this work the possibility of immobilizing predetermined amounts of active enzyme molecules inside a macro- and mesoporous silica monolith in a simple and reproducible way for enzymatic flow-through reactor applications. The work was carried out using the silica monolith MH1,^45,46^ the denpol *de*-PG2_1000_, and the two enzymes horseradish peroxidase isoenzyme C (HRP) and bovine erythrocyte carbonic anhydrase (BCA). The performances of the enzyme reactors in terms of product formation and the stabilities of the immobilized enzymes under different conditions were investigated. Finally, a comparison was made between enzyme reactors containing coimmobilized enzymes and enzyme reactors consisting of sequentially immobilized enzymes. For this comparison, a two-enzyme cascade reaction involving hydrolysis and oxidation steps was used.

## 2 Experimental Section

### 2.1 Materials

With the exceptions mentioned below, all chemicals used were the same as those reported in Ghéczy et al.^42^ In the case of hydrogen peroxide (H_2_O_2_) and 2,2′-azino-bis(3-ethylbenzothiazoline-6-sulfonic acid) diammonium salt (ABTS^2–^(NH_4_^+^)_2_), new batches of the same product were used; no significant difference was observed with respect to the ones used before.^47^

#### 2.1.1 Enzymes and Enzyme Substrates^35^

HRP (EC 1.11.1.7, catalogue number PEO-131, grade I, lot 8153665000, RZ(*A*_403_/*A*_260_) = 3.0, *M*_r_ ≈ 44 000, pI = 8.8)^48,49^ was from Toyobo Enzymes (molar absorption at λ = 403 nm, ε_403_ (HRP) = 102 000 M^–1^cm^–1^).^50^ BCA (EC 4.2.1.1, catalogue number C2624, lot SLBR4228 V, *M*_r_ ≈ 29 000, pI = 5.9),^51,52^ was from Sigma-Aldrich (ε_280_ (BCA) = 56 000 M^–1^cm^–1^).^53^ ABTS (ABTS^2–^(NH_4_^+^)_2_, ≥98%) was from Sigma-Aldrich (catalogue number A1888, lot SLBV6099, *M*_r_ = 548.68), and H_2_O_2_ (35 wt %, stabilized in water) was from Acros Organics (products 202460010 and 202465000, lots A0352305 and A0419470, respectively). For the commercial sources of *p*-nitrophenyl acetate (PNPA), 2′,7′-dichlorodihydrofluorescein diacetate (DCFH_2_-DA), and 2′,7′-dichlorofluorescein diacetate (DCF-DA), see Ghéczy et al..^42^

#### 2.1.2 Chemicals Used for Conjugate Preparation

The deprotected second-generation dendronized polymer (denpol) with an average of 1000 repeating units (r.u), abbreviated as *de*-PG2_1000_ (PDI = *M*_w_/*M*_n_ = 2.4, ε_285_ (per r.u) = 5000 M^–1^cm^–1^),^54^ was a gift from Dr. Daniel Messmer and Prof. A. Dieter Schlüter (ETH Zürich), see Hou et al.^35^ for details. *N*-Succinimidyl 6-hydrazinonicotinate acetone hydrazone (S-HyNic) and *N*-succinimidyl 4-formylbenzoate (S-4FB) were synthesized by Dr. Andrea Grotzky and Dr. Chengmin Hou, respectively, as described previously.^55^ For the commercial sources of the quantification agents 2-hydrazinopyridine dihydrochloride (2-HyPy), 4-nitrobenzaldehyde (4-NiBe), and trypan blue (TB), see Hou et al.^35^

#### 2.1.3 Buffer Solutions

Throughout this work, the buffer solution most frequently used was a phosphate-buffered saline solution (prepared with 100 mM NaH_2_PO_4_ and 150 mM NaCl, pH = 7.2), which was abbreviated as PBS. In the case of the purification and storage of the denpol–enzyme conjugate stock solutions, PBS* was used, which was prepared with 100 mM NaH_2_PO_4_ and 1.15 M NaCl, pH = 7.2. For reactions with PNPA, PB, a phosphate buffer solution *without* added NaCl (prepared with 10 mM NaH_2_PO_4_, pH = 7.2), was applied. These three different phosphate buffer solutions were prepared using Milli-Q water, NaH_2_PO_4_ (≥99.0%, from Sigma-Aldrich), and NaCl (analytic reagent grade, from Fischer), and the pH value was subsequently adjusted with aqueous NaOH (2 M solution). For the additional buffer solutions used during conjugate preparation, see Hou et al.^35^ and Yoshimoto et al.^36^

#### 2.1.4 Stock Solutions

Due to adsorption of *de*-PG2_1000_ on silica,^54^ all denpol stock solutions were stored in polypropylene (PP) tubes at 4 °C before use. HRP (50, 10, or 5 μM) or BCA stock solutions (100 μM) in PBS were prepared by first dissolving the enzymes at 4 (HRP) or 6 (BCA) mg mL^–1^. The solutions were then diluted with PBS to the enzyme concentrations that were determined on the basis of the absorbances measured at λ = 403 or 280 nm, namely, *A*_403_ or *A*_280_, respectively, using the molar absorptions mentioned in section 2.1.1. For the transfer of stock solutions, Gilson Pipetmans and PP tips were used. Denpol stock solutions of 2 mg mL^–1^ were prepared in MOPS buffer at pH = 7.6 (0.1 M MOPS and 0.15 M NaCl). Purified denpol–enzyme conjugate stock solutions (in PBS*) were quantified as described below (section 2.5). To prepare 2 M H_2_O_2_ stock solutions, 35 wt % H_2_O_2_ (11.7 M) was diluted with Milli-Q water. The stock solutions were stable for at least one month upon storage at 4 °C. Further dilutions were freshly prepared on the day of use from the 2 M stock solution.

The following other stock solutions were prepared at room temperature (RT) and then stored at *T* = 4 °C: ABTS^2–^ (20 mM in PBS, freshly prepared every day), PNPA (100 mM in dry acetonitrile), DCFH_2_-DA (5 mM in dry DMSO), and DCF-DA (1 mM in dry DMSO). For the in situ preparation of a 500 μM DCFH_2_ solution from the DCFH_2_-DA stock solution, see the Supporting Information (later in chapter 25). Stock and reaction solutions containing ABTS^2–^, H_2_O_2_, DCFH_2_-DA, or DCFH_2_ were kept light-protected to avoid photochemical reactions that could occur.

#### 2.1.5 Macro- and Mesoporous Silica Monolith (Type MH1)^35^

Cylindrical monoliths of type MH1 (length *l*_m_ ≈ 40 mm and diameter *d*_m_ ≈ 4 mm) were prepared from tetraethyl orthosilicate (TEOS), polyethylene glycol 35 000 (PEG), water, nitric acid (HNO_3_), and cetyltrimethylammonium bromide (CTAB) at a molar ratio of 1:0.52:14.25:0.26:0.027 according to the procedure described by Szymańska et al.^45^ In this previous publication, details about the properties of the monolith are also provided. The monolith of type MH1 has macropores (≈20–30 μm) as well as mesopores (≈20 and ≈2 nm). The estimated internal surface spanned by the macropores in MH1 is *S*_macropores_ = 0.72 m^2^ g^–1^, and the water-accessible volume (abbreviated as *V*_L_) is 4 cm^3^ g^–1^.^35^ For the monolith rods used (25 × 10^–3^ g cm^–1^), *V*_L_ = 100 μL cm^–1^. This value was confirmed independently in this work by determining the total water volume that was taken up through capillary forces by a piece of dry monolith of length *l*_m_ = 10 mm.

### 2.2 Instruments and Methods

#### 2.2.1 UV–Vis Spectrophotometry

Throughout this work, UV–vis absorption spectra were recorded using four types of spectrophotometers. To assess the initial reaction rates with chromogenic enzyme substrates, absorption spectra were recorded every 5 s with either a Specord S600 diode array spectrophotometer (from Analytik Jena) or a Cary 60 spectrophotometer (from Agilent Technologies). The spectra were recorded at RT in disposable polystyrene (PS) cuvettes (semimicro, path length *l* = 1 cm, from BRAND). Initial reaction rates were determined from linear fits of d*A*_λ_/d*t* using the Origin software, where *A*_λ_ was the measured absorbance *A* at wavelength λ at which the formation of the reaction product was convenient to follow (see below).

For slow reactions for which the absorption spectrum was recorded every 5 min only, or to analyze stable solutions that did not change with time, high-resolution spectra were recorded between λ = 250 and 600 nm on a JASCO V-670 UV–vis−NIR spectrophotometer, equipped with an EC-717 Peltier-temperature control. For these measurements, quartz glass cuvettes from Hellma Analytics were used (*l* = 1.0 cm, type 114-10-40 for sample volumes of 1 mL or type 105-201-15-40 for sample volumes of 50 μL).

For quick analyses of concentrated solutions of HRP, BCA, or *de*-PG2_1000_, a Thermo Scientific Nano-Drop ND ONE spectrophotometer was used at *l* = 1 mm (sample volume of 2 μL).

#### 2.2.2 Flow-Through Equipment

Flow-through reactor devices were prepared by placing small pieces of the monolith (*l*_m_ = 5 mm) inside soft LDPE tubing, as described previously,^35^ followed by loading the monoliths with denpol–enzyme conjugates (see later in section 2.6). To wash the monolith pieces, a peristaltic pump (model P-1 from Pharmacia) was used to pass PBS through them (at 200 μL min^–1^). For the connections between the LDPE monolith unit and the pump or the flow-through cell, poly(tetrafluoroethylene) (PTFE) and silicone tubing were used, the final piece being a silicone tube (*d*_inner_ = 2 mm and *d*_outer_ = 4 mm) that fit tightly into the soft LDPE tubing (*d*_inner_ ≈ 4 mm). The connections were sealed by additionally wrapping them with Parafilm. For flow-through reactions with monolith pieces containing immobilized conjugates (run at 200 or 5 μL min^–1^), syringe pumps from either World Precision Instruments (model AL1000) or HARVARD Apparatus (model PHD Ultra or Pump 11 Elite) were used. PP syringes were fitted to the reactors with the same silicone tubing as mentioned above in the case of the peristaltic pump. To assess product formation in flow-through reactions at a flow rate of 200 μL min^–1^, spectra of the outflow were taken online every minute with the Specord or Cary spectrophotometers using a quartz glass flow-through cell (176-765-15-40-QS from Hellma Analytics, *l* = 1.0 or 0.1 cm and *V*_cell_ = 0.11 mL). The cell was fitted to the reactor with silicone tubing as mentioned above. For flow-through reactions at a flow rate of 5 μL min^–1^, the outflow was pooled regularly, and its absorbance was measured in a conventional cuvette.

#### 2.2.3 Scanning Electron Microscopy (SEM)

SEM images were recorded on a SEM Gemini 450 instrument. Prior to the SEM analysis, the samples were coated with 3 nm Pt.

### 2.3 Enzymatic Reactions in Bulk Solution

#### 2.3.1 HRP Activity Measurements with ABTS^2–^/H_2_O_2_

The HRP activity was measured in PS cuvettes using ABTS^2–^ and H_2_O_2_ as substrates by quantifying the formation of ABTS^•–^ (ε_414_(ABTS^•–^) = 36 000 M^–1^ cm^–1^).^56^ The assay conditions were similar to those used before^35,42^ with only slight modifications as follows: a mixture of PBS, pH = 7.2; [ABTS^2–^]_0_ = 1.0 mM; and [H_2_O_2_]_0_ = 0.2 mM was prepared at RT by mixing appropriate volumes of the corresponding stock solutions (see chapter 1 of the Supporting Information for details). Known concentrations of native HRP were correlated to initial rates of ABTS^2–^ oxidation in a calibration curve (Figure S1). With this calibration curve, the amount of HRP that caused an observed initial rate of ABTS^•–^ formation, *v*_in_ (M s^–1^) was determined not only in analyte solutions containing native HRP but also in solutions of modified HRP or denpol–HRP. With this “activity-based” determination of the HRP concentration, the true (molecular) HRP concentration in the analyte solution might deviate due to, for example, the possible presence of inactive HRP molecules or HRP molecules for which access to the active site was hindered.

Using the ABTS conditions mentioned above, the observed rate constant was *k*_obs_(bulk solution) = *v*_in_[HRP]^−1^ = 51 s^–1^ (see Figure S1). HRP-containing analyte solutions were typically measured at an activity-based concentration of [HRP] ≈ 1 nM (corresponding to ≈51 nM ABTS^•–^ s^–1^).

#### 2.3.2 BCA Activity Measurements with PNPA

The activity of BCA was measured in PS cuvettes using PNPA as the substrate. BCA catalyzes the hydrolysis of PNPA to acetate and *p*-nitrophenolate (ε_405_(*p*-nitrophenolate and *p*-nitrophenol) = 10 510 M^–1^cm^–1^ at pH = 7.2).^57^ The assay conditions were the same as those used previously by Yoshimoto et al.:^36^ PB, pH = 7.2, and [PNPA]_0_ = 1.0 mM (1 vol % acetonitrile) at RT (see chapter 2 of the Supporting Information for details). Please note that all rates of reaction measured in the presence of BCA were corrected by the non-negligible autohydrolysis of PNPA in the buffer solution used. With this, a previously determined calibration curve was used.^36^ Similarly to the case of HRP, BCA concentration determinations in analyte solutions were “activity-based” (see section 2.3.1).

Using the PNPA assay conditions mentioned above, the observed rate constant was *k*_obs_(bulk solution) = *v*_in_[BCA]^−1^ = 0.78 s^–1^. BCA-containing analyte solutions were typically measured at an activity-based concentration of [BCA] ≈ 100 nM (corresponding to ≈78 nM PNPA s^–1^).

#### 2.3.3 Cascade Reaction of DCFH_2_-DA with BCA, HRP, and H_2_O_2_

Bulk solution reaction mixtures to which BCA, HRP, DCFH_2_-DA (always 50 μM in PBS, 1 vol % DMSO), and H_2_O_2_ were added initially were prepared in quartz glass cuvettes from the corresponding stock solutions and then measured and analyzed similarly to what we described previously.^42^ The concentrations of BCA (0, 1.0, 1.5, or 3.5 μM), HRP (0, 50, or 100 nM), and H_2_O_2_ (0, 10, or 30 μM) were varied by adding to PBS portions of 100 μM BCA, 5 μM HRP, and 1 mM H_2_O_2_ stock solutions to yield reaction volumes of 1 mL. After the addition of 10 μL of a DCFH_2_-DA stock solution (5.0 mM in DMSO), followed by the placement of a stopper and a few inversions of the cuvette, spectra were recorded every 5 min for 15 h at 25 °C using the JASCO instrument. When applying the cascade reaction for the quantification of H_2_O_2_ ([H_2_O_2_]_0_ = 0 and 1–10 μM), H_2_O_2_ from a 100 μM stock solution (in PBS) was added last to a light-protected PP reaction tube, and spectra were measured after 2 h of storage at RT. For the spectral analysis of the reactions at pH = 7.2 to determine the concentrations of the reaction components, the molar absorption coefficients and isosbestic points obtained in our earlier work were used.^42^

For the protocols for the reactions of DCF-DA (10 μM) and BCA or DCFH_2_ (50 μM) with HRP/H_2_O_2_, see the Supporting Information (described later in chapter 23 or 25, respectively).

### 2.4 Conjugate Preparations

The denpol–enzyme conjugates used in this work were freshly prepared using the same methodology described previously for HRP^35^ and BCA^36^ with only a few modifications.

In a first step, the enzymes were modified with, on average, less than one moiety of 4-formylbenzoate (4FB) to yield enzyme-4FB and the denpol chains were modified with several moieties of 6-hydrazinonicotinate (HyNic) to yield *de*-PG2_1000_-HyNic. The extent of the enzyme and denpol modification with 4FB and HyNic, respectively, was determined by spectral analysis, where the UV–vis absorption spectra of the obtained enzyme-4FB and *de*-PG2_1000_-HyNic solutions were measured and compared with the known reference spectra of the unmodified enzymes,^42^ methyl-4FB,^36^ unmodified denpol (Figure S2),^54^ and denpol-bound HyNic.^35^ Such analysis was already performed previously for BCA-4FB^36^ and was carried out in the present work for the first time for *de*-PG2_1000_-HyNic and HRP-4FB. The results of the spectral analysis agreed well with the more laborious chemical quantification reactions performed using 4-nitrobenzaldehyde (for determining HyNic) and 2-hydrazinopyridine (for determining 4FB) except for HRP-4FB, where the spectral analysis was considered to be more accurate than the chemical quantification due to a side reaction of HRP with excess hydrazine (see chapter 3 of the Supporting Information and Figures S2–S5).

In a second step, aqueous solutions of either purified HRP-4FB (pH = 4.7) or purified BCA-4FB (pH = 7.2) and *de*-PG2_1000_-HyNic (pH = 4.7 or 7.2, respectively) were mixed to induce the conjugation reactions, which resulted in the formation of the conjugates *de*-PG2_1000_-BAH-HRP or *de*-PG2_1000_-BAH-BCA, respectively (see Figure S3 or S5). Through these conjugation reactions, several enzyme molecules of the same type (either HRP or BCA) were covalently attached to the denpol chains via BAH bonds (see Figure 1). After the conjugation reactions were complete, the conjugates were purified from the remaining free enzymes by repetitive ultrafiltration^35,36^ (see chapter 4 of the Supporting Information).

For this work, four conjugates were prepared, which were abbreviated as *de*-PG2_1000_-BAH-HRP_20_, *de*-PG2_1000_-BAH-HRP_40_, *de*-PG2_1000_-BAH-BCA_54_, and *de*-PG2_1000_-BAH-BCA_89_. The subscripted digits of HRP and BCA indicate the average number of active enzyme molecules per denpol chain, as determined experimentally after purification (see section 2.5.1). While the conjugates prepared in this work were more or less the same as those prepared in our previous work (since the conditions for their preparation were the same), the indicated average number of enzyme molecules per denpol chain in this work are based on a quantification of *active* enzyme molecules (see sections 2.3.1 and 2.3.2). In the previous work, the number of denpol-bound enzyme molecules was determined via BAH bond quantification (see section 3.1.3).

### 2.5 Characterization of the Stock Solutions of Purified Conjugates

After purification, the conjugates were left in solution and then used as “stock solutions of purified conjugates”. These stock solutions were characterized in terms of the concentrations of active enzymes and denpol r.u.’s (see chapter 5 of the Supporting Information and Tables S1 and S2).

#### 2.5.1 Active Enzyme Concentrations

The concentrations of active enzymes in the stock solutions of the purified conjugates were determined using calibration curves made with known amounts of native enzymes in a bulk solution and ABTS^2–^/H_2_O_2_ (in the case of HRP) and PNPA (for BCA) as substrates (see sections 2.3.1 and 2.3.2, respectively). The conditions were chosen such that the activity-based concentrations inside the cuvette were ≈1 nM for HRP and ≈0.1 μM for BCA. Note that the activity measurements were started *immediately* after a short period of mixing upon the addition of a small aliquot of the conjugate stock solution to the cuvette containing a solution of the substrate(s). The reason for mentioning this is that the enzyme activity was observed to decrease upon the storage of highly diluted conjugate solutions, while the concentrated conjugate solutions remained stable for a long period of time (see chapter 6 of the Supporting Information and Figure S6). In the case of the purified stock solution of *de*-PG2_1000_-BAH-HRP_20_, [HRP] was determined not only using ABTS^2–^/H_2_O_2_ and the corresponding calibration curve but also with DCFH_2_/H_2_O_2_ and DCFH_2_-MA/H_2_O_2_ (for details, see chapter 7 of the Supporting Information). The obtained results were the same for all three determinations. Additionally, *A*_403_ obtained from the spectral analysis (originating from the Soret band of the heme group of HRP, ε_403_(HRP) = 102 000 M^–1^cm^–1^)^50^ correlated well with [HRP] determined from the activity assay (see the Figure S7).

#### 2.5.2 Repeating Unit Concentration

The concentration of denpol repeating unit (r.u.) in the denpol–enzyme stock solutions was determined from the UV–vis spectra of the purified conjugates, as outlined in the following. First, *A*_354_ (originating from the BAH bond) was measured (ε_354_(BAH) = 29 000 M^–1^cm^–1^).^39^ Assuming that the ratio between the concentration of stable BAH bonds and the concentration of the denpol r.u., [BAH]/[r.u.], did not change during purification, measuring *A*_354_ of both the conjugation reaction mixtures (“rm”) after the completion of the reaction and of the resulting stock solutions of the purified conjugates (“pur”) allowed the quantification of the denpol recovery yield after purification (independent from ε_354_(BAH), as it was considered to be the same before and after purification), i.e., [BAH]_pur_/[r.u.]_pur_ = [BAH]_rm_/[r.u.]_rm_. The concentration of the denpol r.u. in *de*-PG2_1000_-HyNic used in the conjugation reaction mixtures, [r.u.]_rm_, was known from its quantification with the trypan blue assay.^36,42^ Therefore, [r.u.]_pur_ = [BAH]_pur_ × ([r.u.]_rm_/[BAH]_rm_). This method worked well for the denpol–BAH–BCA conjugates, since BCA does not absorb at λ = 354 nm.^36^ For the denpol–BAH–HRP conjugates, however, the contribution of HRP to *A*_354_ had to be taken into account. Careful spectral analysis and the use of a control experiment in which *de*-PG2_1000_-HyNic was exposed to unmodified native HRP (see chapter 8 of the Supporting Information and Figure S7) showed that it is possible to determine [r.u.] in *de*-PG2_1000_-BAH-HRP with a slightly modified, but still simple and quick, spectrophotometric method. In this case, the activity-based HRP concentration (see section 2.5.1) and the *A*_354_/*A*_403_ ratio (instead of their absolute values) were considered (see chapter 9 of the Supporting Information). For the stock solution of *de*-PG2_1000_-BAH-HRP_20_, the trypan blue assay was also used. The [r.u.] value was the same as that determined with the spectral method, confirming the validity of the spectral method. The latter was thus used for the analysis of all other denpol–enzyme conjugate stock solutions prepared in this work (see chapters 10 and 11 of the Supporting Information and Table S1).

### 2.6 Controlled Immobilization of Conjugates in Monolith MH1 for Use as Enzymatic Flow-Through Reactors

#### 2.6.1 General Protocol

To load the monolith MH1 with *de*-PG2_1000_-BAH-enzyme conjugates, a defined volume of an aqueous conjugate solution of a defined conjugate concentration (called “conjugate incubation solution”) was added to a monolith piece of defined length and diameter that was placed inside a LDPE tube (see chapter 12 of the Supporting Information and Figures S8 and S9). Before the addition of the conjugate incubation solution, the monolith inside the PE tubing was washed with Milli-Q water (at a rate of 2 mL min^–1^ for 5 min) and then dried with a nitrogen gun (until the monolith weight was the same as it did before washing). The general immobilization procedure is sketched in Figure 1.

The aqueous conjugate incubation solution was pipetted directly onto one of the two front sides of the monolith inside the LDPE tubing. The amount of the added conjugate solution was 100 μL per centimeter of the monolith length (see section 2.1.5). If not mentioned otherwise, monolith pieces of *l*_m_ = 5 mm were used, that is, the volume of the conjugate solution added—and taken up completely by the monolith piece—was *V*_L_ = 50 μL. For the later washing steps and for use as enzyme reactor units, solutions that were pumped though the monolith were always pumped from the same front side as the monolith was loaded with the conjugate solution.

After the monolith piece was filled with the conjugate incubation solution, the LDPE tubing holding the monolith piece was closed on both ends (inlet and outlet) with Parafilm and incubated at RT for 3 h. Then, it was washed with PBS at a rate of 200 μL min^–1^ using a peristaltic pump overnight (15 h at RT) unless otherwise mentioned. After washing, the prepared enzyme reactor was stored in the wet state (filled with PBS and closed with Parafilm) at 4 °C until further use.

**Figure 2:**
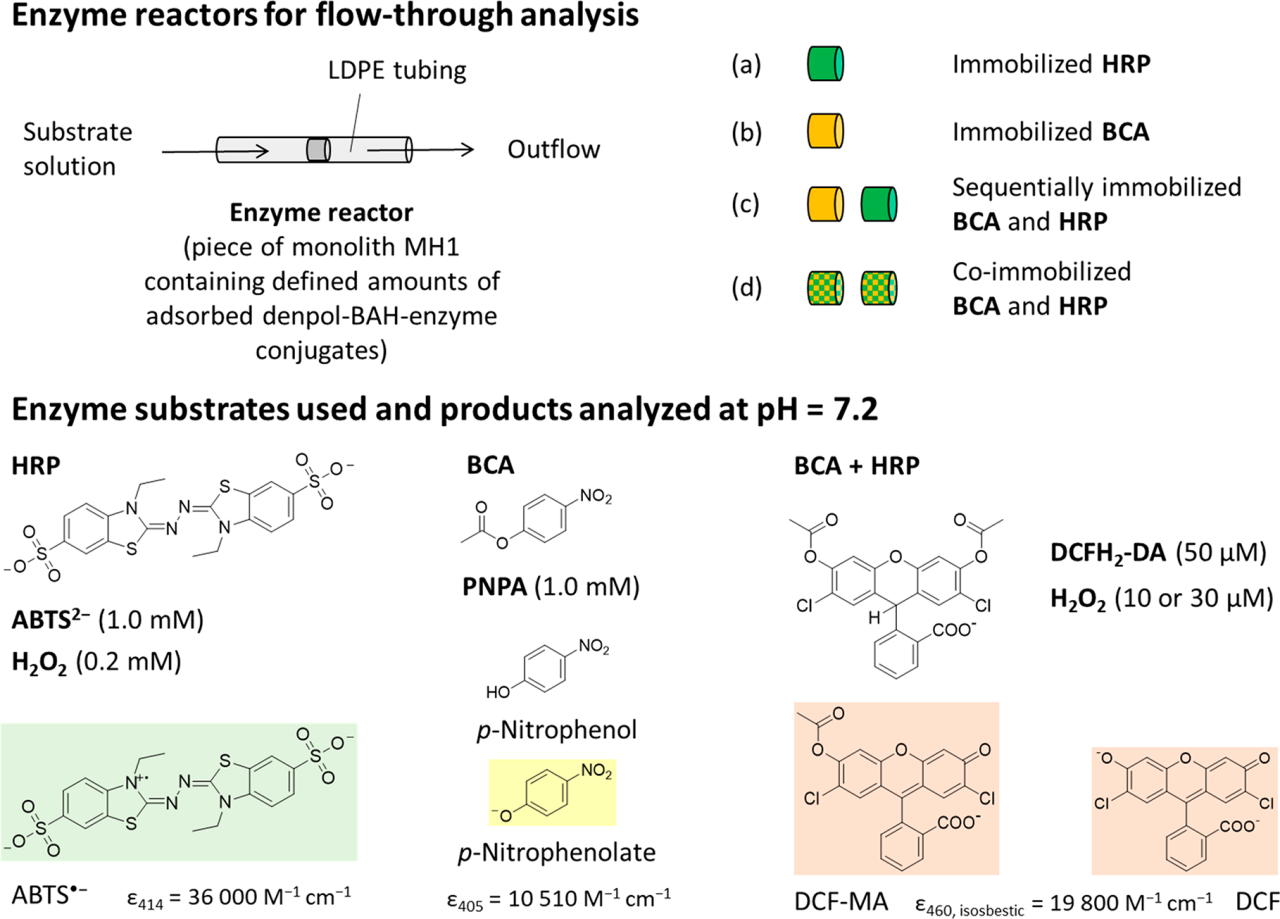
(Upper half) Schematic representation of the four types of enzymatic flow-through reactor systems prepared with the help of LDPE tubing, see section 2.6. (Lower half) Chemical structures of both the enzyme substrates used to quantify the performance of the immobilized enzymes and the reaction products that were analyzed spectrophotometrically in the enzyme reactor outflows. The same substrates were also used to determine the activities of the two enzymes (HRP and BCA) in the bulk solution at pH = 7.2. (a) HRP reactor analyzed with ABTS^2–^/ H_2_O_2_. ABTS^•–^ was detected at λ = 414 nm. (b) BCA reactor analyzed with PNPA. *p*-Nitrophenolate was detected at λ = 405 nm. (c) Sequentially and (d) coimmobilized BCA and HRP reactor systems analyzed with DCFH_2_-DA/H_2_O_2_. DCF-MA and DCF were detected at λ_iso_ = 460 nm. The monolith piece of the enzyme reactors had a diameter *d*_m_ ≈ 4 mm and a typical length *l*_m_ = 5 mm (*V*_L_ = 50 μL), see Figure 1. For panels a and b, a flow rate of 200 μL min^–1^ was used. For the cascade reaction with the enzyme reactor systems in panels c and d, the flow rate was 5 μL min^–1^. See section 2 for details.

For the different types of enzyme reactors prepared, see Figure 2. Details about the conjugate incubation solutions used and the different enzyme reactor types are described in the following sections 2.6.2–2.6.4.

#### 2.6.2 Individual Immobilization of HRP or BCA

To prepare an individual enzymatic flow-through reactor with either immobilized *de*-PG2_1000_-BAH-HRP or *de*-PG2_1000_-BAH-BCA (*l*_m_ = 5 mm and *V*_L_ = 50 μL, see reactor types a and b in the upper half of Figure 2), the preparation and handling of the conjugate incubation solutions were as follows. The conjugate stock solutions (stored at 4 °C in PBS*) were first allowed to reach RT, then diluted with PBS and PBS* such that the content of the two buffers after dilution was in all cases 60 vol % PBS and 40 vol % PBS*, respectively (resulting in consistent incubation conditions for all conjugate incubation solutions of 0.1 M phosphate and 0.55 M NaCl, pH = 7.2). Immediately after dilution, the resulting conjugate incubation solutions were vortexed for a few seconds and then added to a monolith piece. The enzyme concentration in these incubation solutions was varied. For *de*-PG2_1000_-BAH-HRP, [HRP] = 50–500 nM ([r.u.] = 2.5–25 μM). For *de*-PG2_1000_-BAH-BCA, [BCA] = 1.0–5.1 μM ([r.u.] = 18–76 μM). As an example of the procedure described, a typical incubation solution of *de*-PG2_1000_-BAH-HRP_20_ with [HRP] = 500 nM was prepared by first mixing 48 μL of PBS with 14 μL of PBS*, followed by adding 18 μL of the conjugate stock solution (made with PBS*). From these 80 μL conjugate incubation solutions, 50 μL was added to the monolith piece. To prepare the other conjugate incubation solutions with different HRP concentrations, the amounts of the stock solution and PBS* were adjusted accordingly. For control experiments, enzyme-free monolith pieces were prepared in the same way, with the exception that a buffer solution was used instead of the conjugate incubation solution.

#### 2.6.3 Sequential Immobilization of BCA and HRP

In experiments with enzyme reactor systems that contained sequentially immobilized enzymes (reactor type **c** in the upper half of Figure 2), two separately prepared and washed enzyme reactors were connected. The first reactor always contained immobilized BCA, and the second always contained immobilized HRP. The BCA reactor was prepared using a conjugate incubation solution containing *de*-PG2_1000_-BAH-BCA_89_ with [BCA] = 5.1 μM. To prepare the HRP reactor, a conjugate incubation solution containing *de*-PG2_1000_-BAH-HRP_40_ with [HRP] = 310 nM was applied. For the typical size of the monolith pieces used (*l*_m_ = 5 mm and *V*_L_ = 50 μL), the amounts of active BCA and HRP in the two incubation solutions were 256.5 pmol and 15.5 pmol, respectively.

To vary the residence time (τ) at constant flow rate, two experiments were carried out with monolith pieces of varied lengths. In the first experiment, the monolith piece containing immobilized BCA had a length *l*_m_(BCA) = 10 mm (*V*_L_(BCA) = 100 μL), while for the second one containing immobilized HRP the length was *l*_m_(HRP) = 5 mm (*V*_L_(HRP) = 50 μL). In this case, τ(BCA) = 2 × τ(HRP). In the second experiment, *l*_m_(BCA) = 5 mm (*V*_L_(BCA) = 50 μL) and *l*_m_(HRP) ≈ 6.5 mm (*V*_L_(HRP) ≈ 65 μL). In this case, τ(HRP) ≈ 1.3 × τ(BCA).

#### 2.6.4 Coimmobilization of BCA and HRP

To prepare the enzyme reactors containing coimmobilized enzymes, the same types of conjugate incubation solutions that were used for the sequential immobilization were first prepared separately, comprising either a *de*-PG2_1000_-BAH-BCA or *de*-PG2_1000_-BAH-HRP conjugate (see section 2.6.3). The two solutions were then mixed. The mixture contained *de*-PG2_1000_-BAH-BCA_89_ at [BCA] = 2.55 μM and *de*-PG2_1000_-BAH-HRP_40_ at [HRP] = 155 nM. This mixed conjugate incubation solution was then added to two monoliths, each with *l*_m_ = 5 mm and *V*_L_ = 50 μL. The two enzyme reactors were washed individually, as described above, and then connected for flow-through applications. Compared to the sequentially immobilized enzyme reactors (see section 2.6.3), the two coimmobilized enzyme reactors contained the same total amounts of enzyme molecules, but both enzymes were equally distributed over both monolith pieces instead of inside one monolith piece. This means that at equal flow rate the residence time, τ, for the coimmobilized enzyme reactors was twice as high as that in the case of the sequentially immobilized enzyme reactors.

#### 2.6.5 Evaluation of the Enzyme Immobilization Yield and Leakage from the Monolith Under Flow-Through Conditions

The enzyme immobilization yields in the monolith pieces were calculated using the mass balance by considering the activity-based amount of enzyme present in the conjugate incubation solution that was added to the monolith piece and the amount of enzyme that leaked from the monolith piece during the washing step and therefore was found in the outflow when the monolith piece was flushed with the buffer solution (determined with the ABTS or PNPA assay, see section 2.3.1 or 2.3.2, respectively). To quantify the amount of enzymes that leaked during the washing step, the eluate was assayed in continuously pooled fractions until activity was no longer detected. During the flow-through use of the enzyme reactors (see later sections 2.8.3 and 2.8.4), aliquots of the outflow were collected and assayed to detect active enzyme molecules that potentially leaked from the monolith pieces during operation (flow-through assay). In both cases (buffer washing and flow-through assays), the flow rate was set to 200 μL min^–1^, which corresponded to a cross-sectional flow of 1.6 mL min^–1^ cm^–2^. For detailed washing protocols and the analysis of the eluates, see chapter 13 of the Supporting Information and Figure S10.

### 2.7 Conjugate Adsorption for the SEM Analysis

Cut pieces of monolith MH1 (*l*_m_ = 5 mm and *d*_m_ ≈ 4 mm) and flat circular glass coverslips (diameter of 5 mm and thickness of 0.16–0.19 mm, from Science Services) were exposed to conjugate incubation solutions of either *de*-PG2_1000_-BAH-HRP_20_ ([HRP] = 0.5 μM and [r.u.] = 25 μM) or *de*-PG2_1000_-BAH-BCA_54_ ([BCA] = 4.1 μM and [r.u.] = 77 μM) in PBS. In the case of the monolith pieces, the pore volume that was accessible to an aqueous solution (*V*_L_ = 50 μL for *l*_m_ = 5 mm and *d*_m_ ≈ 4 mm) was filled by adding the aqueous conjugate incubation solution to the monolith piece with a pipet. The added solution was taken up by the monolith piece through capillary forces and remained inside the monolith piece (see also section 2.6). In the case of the coverslips, they were immersed in 100 μL of the conjugate incubation solution. The monolith pieces and the coverslips were placed into separate 2 mL PP tubes. After 3 h at RT (to allow for conjugate adsorption), the monolith pieces and the coverslips were washed twice with PBS and four times with Milli-Q water (1 mL each, with gentle shaking of the PP tubes), then predried with a N_2_-gun. With this procedure, the monolith pieces turned snow-white. Further drying was applied using a vacuum pump for 2 h (≈1 mbar). Afterward, the monolith pieces were cut into smaller parts, followed by Pt-coating and SEM analysis; the coverslips were Pt-coated at full size. For the reference measurements of “blank surfaces”, the same procedures were applied using PBS instead of a conjugate incubation solution.

### 2.8 Flow-Through Reactions Using the Prepared Enzyme Reactors

#### 2.8.1 General Methods

Before starting flow-through measurements, the prepared enzyme reactors (stored immersed in PBS at 4 °C) were first washed for 10 min with PBS using a peristaltic pump at a flow rate of 200 μL min^–1^. Afterward, the PBS solution was removed from the tube holding the enzyme reactor using a twisted paper tissue. With this treatment, the enzyme reactor remained wet, i.e., it never dried completely. For measurements involving two enzyme reactors, the reactors were connected by silicone tubing. Finally, the enzyme reactors were attached to light-protected syringes that were filled with the respective substrate solutions, which had the same composition as that in the case of the substrate solutions used to determine the activities of the enzymes in the bulk solution (see sections 2.3.1–2.3.3). The respective substrate solutions were pumped through the enzyme reactors using a syringe pump, and the UV–vis absorption spectrum of the outflow was recorded online by passing the outflow through a flow-through cell (see section 2.2.2). The first spectrum was recorded after the cell was filled completely with substrate solution. For flow-through measurements with a very low flow rate (5 μL min^–1^), see section 2.8.4. After the flow-through measurements were completed, the silicone tubing was removed to separate the enzyme reactors, and the reactors were washed individually with PBS for 10 min using the peristaltic pump (200 μL min^–1^). The PBS-filled enzyme reactors were stored until further use at 4 °C (closed with Parafilm).

#### 2.8.2 Analysis of the Performance of the HRP Reactor Using ABTS^2–^/H_2_O_2_

The activity of HRP immobilized in the enzymatic flow-through reactors was determined with the same substrate solution as that used to measure the activity of HRP in the bulk solution, specifically PBS, pH = 7.2, [ABTS^2–^]_0_ = 1.0 mM, and [H_2_O_2_]_0_ = 0.2 mM at RT (see section 2.3.1 and Figure 2). The substrate solution was passed through the HRP reactor at a flow rate of 200 μL min^–1^ with the help of a syringe pump, and the UV–vis absorption spectrum was measured using the flow-through cell (*l* = 0.1 cm). A background spectrum of the substrate solution was also recorded, and Δ*A*_414_ = *A*_414_(outflow) – *A*_414_(background) was typically recorded for 20–50 min of continuous flow. In control measurements, monolith pieces that were either treated with incubation solutions of native HRP (in PBS) or with PBS only, followed by extensive washing with PBS at a rate of 200 μL min^–1^, were analyzed in the same way. As a result, no significant *A*_414_ was detected in the outflows of these two controls.

##### 2.8.2.1 Analysis Conditions

The (repeatedly) determined Δ*A*_414_ values in the outflow were single-point data with respect to the progression curve of the reaction.^58^ To stay close to initial reaction rate conditions,^59^ the flow rate for a given enzyme reactor length and enzyme loading was chosen such that the substrate conversion within the residence time inside the reactors (τ) was low (conversion <15% [ABTS^2–^]_0_). See also section 3.3.1.

#### 2.8.3 Analysis of the Performance of the BCA Reactor Using PNPA

The activity of BCA immobilized in the enzymatic flow-through reactors was measured using the same substrate solution as that applied to measure the activity of BCA in bulk solution, specifically PB, pH = 7.2, and [PNPA]_0_ = 1.0 mM at RT (see section 2.3.2 and Figure 2). The substrate solution was passed through the BCA reactor at a flow rate of 200 μL min^–1^ using a syringe pump. The nonenzymatic hydrolysis of PNPA was taken into account by measuring *A*_405_ of the outflow from an “empty” monolith of the same length (no BCA) using the same substrate solution at the same time after the substrate solution preparation. To obtain the net BCA-catalyzed change in *A*_405_ during the passage of the PNPA solution through the monolith reactor, that is, Δ*A*_405_, the background measurements were subtracted from the measurements with immobilized BCA.

##### 2.8.3.1 Analysis Conditions

As for the HRP reactors (section 2.8.2), the conditions were chosen such that the extent of substrate conversion was low (below 6% [PNPA]_0_), indicating that the reaction conditions yielded initial reaction rates.

#### 2.8.4 Analysis of the Cascade Reaction with the Enzymatic Flow-Through Reactors Using DCFH_2_-DA and H_2_O_2_ as Substrates

**Figure 3:**
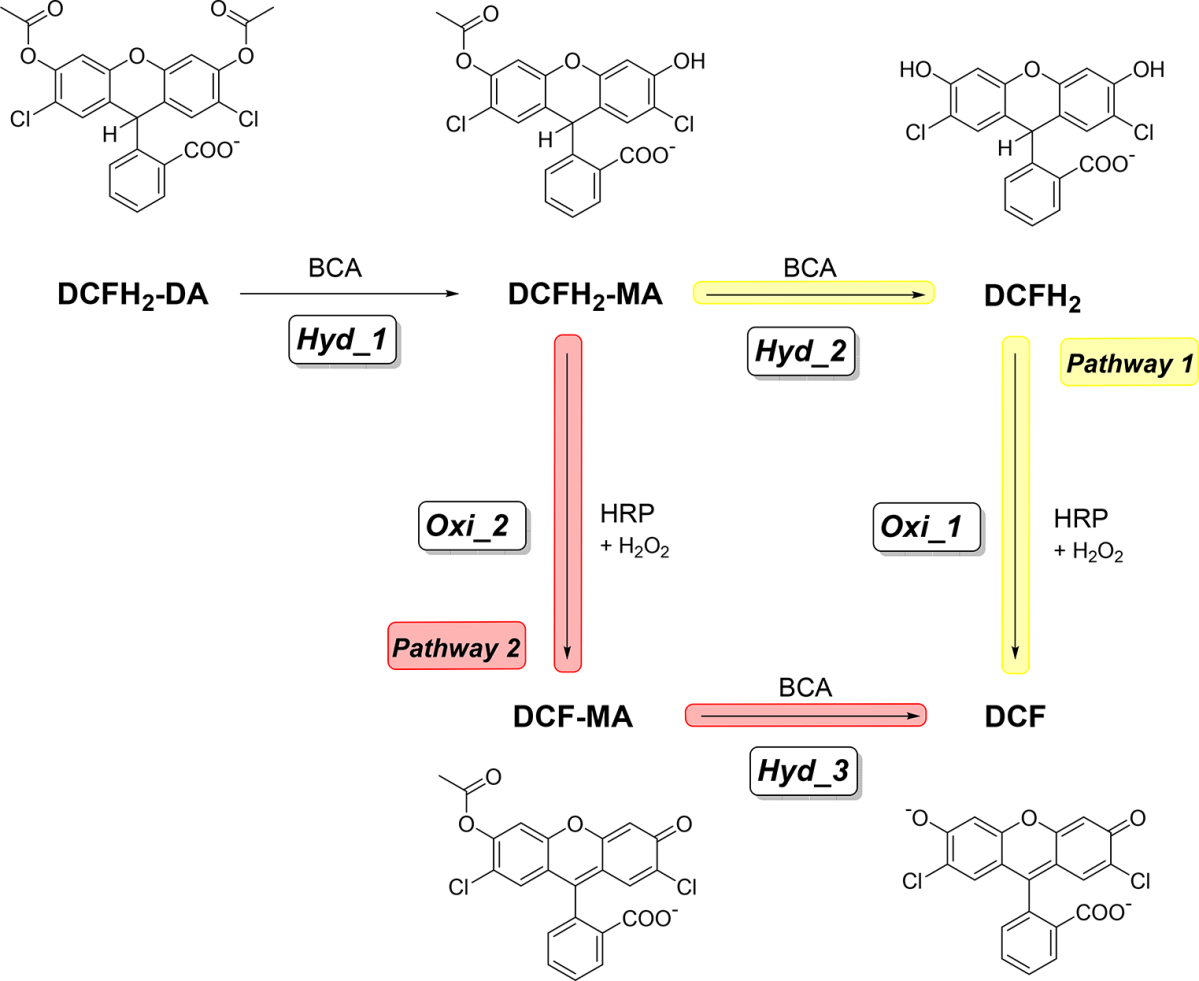
Summary of the two-enzyme cascade reaction system applied in this work to analyze the performances of two enzyme reactor systems consisting of BCA and HRP; see Figure 2c and d and Ghéczy et al.^42^ In a reaction mixture containing DCFH_2_-DA, H_2_O_2_, BCA, and HRP at pH = 7.2 and RT, the hydrolysis of DCFH_2_-DA to DCFH_2_-MA and the hydrolysis of DCFH_2_-MA to DCFH_2_ are catalyzed by BCA (hydrolysis steps 1 and 2, abbreviated as *Hyd_1* and *Hyd_2*). Depending on the amounts of BCA, HRP, and H_2_O_2_ present, the HRP-catalyzed oxidation of DCFH_2_ to DCF (oxidation step *Oxi_1*) occurs slower or faster than the HRP-catalyzed oxidation of the intermediate DCFH_2_-MA to DCF-MA (*Oxi_2*), which eventually undergoes hydrolysis to DCF (*Hyd_3*). Therefore, once DCFH_2_-MA is formed, there are two pathways to yield DCF, *pathway 1* via DCFH_2_ and *pathway 2* via DCF-MA. Please note that a possible further oxidation of DCF to the oxidized forms of DCF are omitted here since the overoxidation of DCF only occurs at high concentrations of HRP and H_2_O_2_, which were not used in the present work. Moreover, in the present work, the conditions were chosen such that the reaction proceeded predominately along *pathway 2*; see the text and chapter 22 of the Supporting Information for details. The scheme is a simplified version of the one published by Ghéczy et al.^42^ Reproduced in part from ref (42) with permission from the Royal Society of Chemistry.

Although only two enzymes, namely BCA and HRP, take part in the cascade reaction, as shown in Figure 3, there are three possible hydrolysis steps that are catalyzed by BCA and two possible oxidation reactions that are catalyzed by HRP with H_2_O_2_ as limiting oxidant.^42^ The performances of the two immobilized enzymes in this cascade reaction were determined at a flow rate of 5 μL min^–1^ using one of the following two substrate solutions: PBS (1 vol % DMSO), pH = 7.2, [DCFH_2_-DA]_0_ = 50 μM, and [H_2_O_2_]_0_ = 10 or 30 μM at RT (see section 2.3.3 and Figure 2). Since the flow rate was low (5 μL min^–1^), a few modifications were applied as compared to the individual enzyme reactors used at a flow rate of 200 μL min^–1^. After the enzyme reactors were connected to the syringe, the chosen substrate solution was first pumped at a rate of 100 μL min^–1^ toward the enzyme reactors until the substrate solution within the tubing approached the first monolith piece (without touching it). Then, the flow was set to 5 μL min^–1^, and the entire setup was light-protected. The outflow was analyzed by collecting volumes of 70 μL, followed by immediately recording the UV–vis absorption spectrum against PBS (no use of a flow-through cuvette due to the low flow rate) using a microcuvette (*l* = 1 cm). Measurements of pooled outflows were repeated every 30 min for an additional 5 h of continuous flow.

##### 2.8.4.1 Analysis Conditions

*A*_460_ and *A*_503_ of the spectra were monitored over time and found to reach stable values after ≈4 h of continuous flow. Thus, the average values for the steady-state condition reached after 4, 4.5, and 5 h of flow were considered for the analysis (residence time τ = 20 min per centimeter of the monolith piece length). As for the cascade reaction carried out with enzymes in the bulk solution, the previously determined molecular absorptions and isosbestic points at pH = 7.2 were applied to determine the concentrations of the reaction components (see Ghéczy et al.^42^).

## 3 Results and Discussion

### 3.1 Conjugate Preparation and Characterization in an Aqueous Solution

#### 3.1.1 Overview

In this work, two different types of denpol–enzyme conjugates were prepared in an aqueous solution using the denpol *de*-PG2_1000_ and HRP or BCA, then purified from free enzyme molecules (see section 3.1.2). During the synthesis, purification, and storage, the conjugates were always kept dissolved in an aqueous solution, i.e., they were never isolated as solid, dried compounds.

Four aqueous conjugate stock solutions containing *de*-PG2_1000_-BAH-HRP_20_, *de*-PG2_1000_-BAH-HRP_40_, *de*-PG2_1000_-BAH-BCA_54_, or *de*-PG2_1000_-BAH-BCA_89_ were prepared. The subscripts indicate the average number of denpol r.u.’s and fully active enzyme molecules per denpol chain, as obtained after purification (see sections 1 and 3.1.3).

#### 3.1.2 Conjugate Preparation

Several enzyme molecules of the same type (i.e., either HRP or BCA) were attached covalently to *de*-PG2_1000_ along the denpol chain using the UV–vis-quantifiable BAH bond-linking chemistry, as described in detail previously^35,36,39,42^ (see Figure 1). The formation of the conjugate is simple. After the separate modification of (i) some of the many primary amines present in *de*-PG2_1000_ with S-HyNic and (ii) on average less than one of the accessible lysine residues present in HRP or BCA with S-4FB in slightly alkaline aqueous solution, simply mixing an aqueous solution of purified HRP-4FB or purified BCA-4FB and an aqueous solution of purified *de*-PG2_1000_-HyNic at pH = 4.7 (for HRP) or 7.2 (for BCA) resulted in the formation of the conjugates *de*-PG2_1000_-BAH-HRP or *de*-PG2_1000_-BAH-BCA, respectively. The protocols for the formation and purification of the conjugates were very similar to those used in our previous works.^35,36^ The main difference with respect to the previous works was the quantification of both the extent of enzyme modification with 4-FB and the modification of the denpol with HyNic before the conjugation reaction was initiated. These determinations were made by direct spectral analysis (not by a chemical reaction with 2-hydrazinopyridine or 4-nitrobenzaldehyde). The direct spectral analysis only required (i) recording of the absorption spectra of the obtained solutions of purified HRP-4FB, BCA-4FB, and *de*-PG2_1000_-HyNic and (ii) performing the trypan blue assay to determine [r.u.] in the *de*-PG2_1000_-HyNic solution. After the conjugation reaction reached an equilibrium state, the conjugates were purified from remaining free enzymes by repetitive ultrafiltration. For details, see section 2.4 and chapter 4 of the Supporting Information.

#### 3.1.3 Characterization of the Purified Conjugate Stock Solutions

The denpol–enzyme conjugate stock solutions obtained after conjugate purification were characterized by the two molar concentrations relevant for achieving controlled conjugate immobilization: (i) the concentration of active enzyme molecules and (ii) the concentration of denpol repeating units (see the Table S1). The concentration of active enzyme molecules was determined using activity assays and calibration curves made with native enzymes in the bulk solution, yielding the “activity-based” enzyme concentrations [HRP] (with ABTS^2–^/H_2_O_2_ as the substrate) and [BCA] (with PNPA as the substrate). In the case of HRP, DCFH_2_ was also used as the reducing substrate instead of ABTS^2–^. The determination of [HRP] in the stock solution of *de*-PG2_1000_-BAH-HRP_20_ was shown to be independent from the reducing substrate used. Therefore, for the two stock solutions containing either *de*-PG2_1000_-BAH-HRP_20_ or *de*-PG2_1000_-BAH-HRP_40_, the determined activity-based concentration of HRP can be considered as the true concentration of catalytically active HRP, as it is independent from the chemical structure of the substrate used in the assay (see section 2.5.1 and chapter 7 of the Supporting Information). The same was assumed to be the case for the activity-based determination of [BCA] in the two denpol–BCA conjugate stock solutions containing either *de*-PG2_1000_-BAH-BCA_54_ or *de*-PG2_1000_-BAH-BCA_89_. To quantify the r.u. concentration in the denpol–enzyme conjugate stock solutions, the trypan blue assay and a spectral method were used (see section 2.5.2). Note that in our previous investigations the average number of conjugated enzyme molecules per denpol chain was quantified differently, either (i) via the amount of BAH bonds (calculated by using ε_354_(BAH) = 29 000 M^–1^cm^–1^, assuming in most cases one bound enzyme molecule per BAH bond)^35,39^ or (ii) by determining the number of enzyme molecules used for the conjugate preparation and subtracting from this value the determined number of enzyme molecules that did not bind to the denpol during the conjugate formation and were removed during conjugate purification (mass balance approach).^36,42^ With the activity-based determinations of the enzyme concentration used in this work, the concentration of conjugated and active enzyme molecules is obtained. This is the relevant concentration of enzymes as biocatalysts, ignoring the possible presence of inactivated or inaccessible enzyme molecules. Moreover, active enzyme attachment via more than one BAH bond per enzyme molecule would not falsify the enzyme concentration determination. For further details and a discussion about the conjugate characterization and the yields observed upon conjugate preparation, see chapters 10 and 11 of the Supporting Information, respectively.

#### 3.1.4 Storage Stability of the Conjugate Stock Solutions

The denpol–enzyme conjugates retained their full enzymatic activity for more than one year if stored as stock solutions at pH = 7.2 and 4 °C (phosphate buffer). For specific data for the stock solutions of *de*-PG2_1000_-BAH-HRP_20_ and *de*-PG2_1000_-BAH-BCA_54_ that were obtained after purification at [HRP] = 2.2 μM and [BCA] = 7.4 μ and 18 months of storage at *T* = 4 °C, see chapter 14 of the Supporting Information and Figure S11. The storage stabilities of the denpol–enzyme conjugates agree with the stabilities of free HRP and BCA at comparable concentrations in phosphate buffer solutions of similar pH value. Therefore, under the conditions used, (i) denpol-bound HRP and BCA are neither more nor less stable than the free enzymes and (ii) the denpol has no detrimental effect on the two enzymes. Both findings are beneficial for the enzyme immobilization method, which we developed and applied in this work. Once a sufficiently large volume of a denpol–enzyme stock solution is prepared, it can be used for a long period of time if stored at 4 °C. Such stock solutions are the “starting point” for preparing well-defined enzymatic flow-through reactors using an extremely simple and highly controlled procedure (see section 3.2 as follows).

### 3.2 Controlled Conjugate Adsorption on Silica Surfaces

#### 3.2.1 SEM Analysis and Estimations of the Silica Surface Occupancy of the Conjugates

As demonstrated before,^35−38^*de*-PG2_1000_-BAH-enzyme conjugates adsorbed readily on silica surfaces when a silica surface was simply exposed to an aqueous solution containing the conjugates. Using a transmission interferometric adsorption sensor and atomic force microscopy measurements, it was shown that a stable surface coating of a relatively homogeneous monolayer can be obtained if flat silica surfaces were exposed to an excess volume of an aqueous *de*-PG2_1400_-BAH-HRP_108_ solution to achieve maximal conjugate adsorption (see Küchler et al.^37^). However, in the present work, a *subsaturation* of the internal monolith surface by the conjugates was targeted (not maximal coverage) to establish a protocol that allowed controlled and simple enzyme immobilization using identical volumes of conjugate solutions of the desired enzyme concentration for monolith pieces of a desired length (usually *l*_m_ = 5 mm and *V*_L_ = 50 μL; see section 3.2.2).

##### 3.2.1.1 SEM Analysis

**Figure 4:**
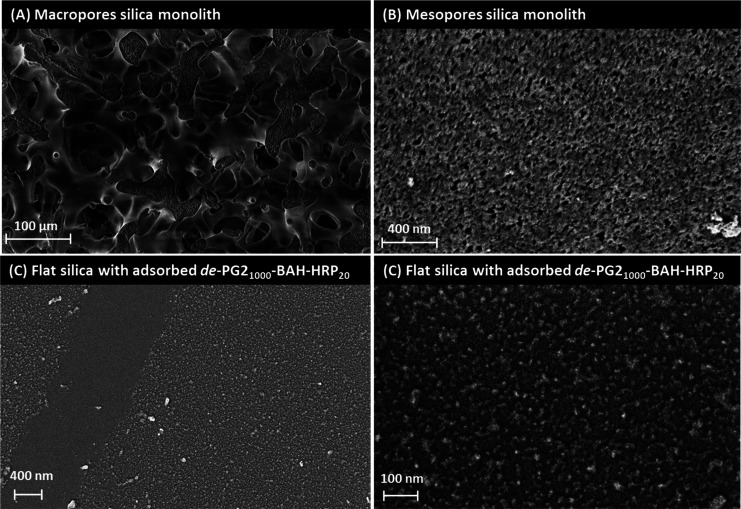
SEM images of (A and B) the macro- and mesoporous monolith MH1, respectively, and (C and D) the denpol–enzyme conjugate *de*-PG2_1000_-BAH-HRP_20_ adsorbed on flat silica glass coverslips. In panel A, the bicontinuous internal structure spanned by macropores with sizes of ≈20–30 μm can be seen. The denpol–enzyme conjugates were immobilized on this internal silica surface under subsaturating conditions via a multitude of noncovalent interactions. In (B), the mesoporous structure of the internal monolith surface can clearly be seen (average pore diameter ≈20 nm); for information about a previous quantification of the mesopores by other means, see also Szymańska et al.^45^ However, adsorbed denpol–HRP conjugates are not visible due to the rough internal surface and the comparatively low height of the adsorbed conjugates. (C) Under saturating conditions, the vast majority of the adsorbed conjugates appeared as a monolayer, i.e., without much overlapping of the conjugates. The corresponding images of adsorbed denpol–BCA conjugates were very similar. (D) Magnification of an area that contained adsorbed denpol–HRP. For additional SEM images, see the Figures S12–S16.

SEM images of the monolith MH1 were recorded. For a view into the meandering macrochannels, see Figure 4A. Mesopores with diameters of about 20 nm were visualized for the first time. The SEM analysis confirmed the existence of such pores, which were suggested on the basis of earlier nitrogen adsorption and desorption measurements of the same type of monolith^45^ (see Figure 4B, chapter 15 of the Supporting Information and Figures S12 and S13). Mesopores with diameters of only about 2 nm,^45^ could not be visualized by SEM. When images of monoliths containing adsorbed conjugates were recorded (the adsorption was confirmed by enzyme activity measurements, see later section 3.3), the adsorbed conjugates could not be identified by SEM. With their comparatively low height (∼5 nm, as determined previously by AFM measurements in dried state),^37^ the denpol–enzyme conjugates could not be distinguished from the rough internal surface of the monolith. In an alternative attempt, we recorded SEM images of flat silica coverslips that were saturated with either *de*-PG2_1000_-BAH-BCA_54_ or *de*-PG2_1000_-BAH-HRP_20_. In both cases, a monolayer of the conjugates was obtained and the individual conjugates were clearly identified as small objects; only a small fraction of overlapping conjugates was present. The accumulation of large aggregates of conjugates was not observed, see the case of *de*-PG2_1000_-BAH-HRP_20_ in Figures 4C and D and S14. The corresponding images for *de*-PG2_1000_-BAH-BCA_54_ were very similar (Figures S15 and S16). The conjugate layers were found to be homogeneous and rather densely (but not perfectly) packed. In the region of the scratches that were present, the difference between adsorbed conjugates and the coverslip background is obvious (Figure 4C, left part). We assume that the coverage of the inner surface of the monolith after exposure to an aqueous conjugate solution is similar to the coverage of the coverslips, i.e., no extensive overlapping of adsorbed conjugates. However, in contrast to the experiments regarding conjugate immobilization on coverslips, there was no intention to saturate the inner surface of the monolith with the conjugate.

##### 3.2.1.2 Estimation of the Silica Surface Occupancy of the Conjugates

Using the SEM images shown in Figure 4C and D, we estimated the maximal amount of conjugate for an overlap-free conjugate adsorption on the surface to be 44 pmol r.u. cm^–2^ (see chapter 16 of the Supporting Information). This defensive estimation is a very rough estimation that does not consider any differences that might originate from the enzyme type and the average number of enzyme molecules bound per *de*-PG2_1000_ chain.

With a surface occupancy of 44 pmol r.u. cm^–2^, the calculated amount of adsorbed HRP molecules in the case of the conjugate *de*-PG2_1000_-BAH-HRP_20_ was about 0.9 pmol HRP cm^–2^ accessible inner monolith surface. Considering the total inner surface area of the macropores present in a monolith piece of length *l*_m_ = 5 mm (90 cm^2^) and the total volume of this monolith piece that is accessible to an aqueous conjugate solution (50 μL), a surface coverage of about 0.9 pmol HRP cm^–2^ might be achieved by complete conjugate adsorption from 50 μL of a conjugate solution containing about 80 μM r.u. ([r.u.]_max_, corresponding to 80 pmol HRP in the case of *de*-PG2_1000_-BAH-HRP_20_; see chapter 16 of the Supporting Information for the calculation). If the adsorption of the conjugate from the conjugate incubation solution onto the inner surface of a monolith piece occurs quantitatively, the amount of adsorbed HRP should be controllable through the enzyme concentration of the conjugate incubation solution as long as [r.u.] < 80 μM = [r.u.]_max_. This was the idea behind the controlled immobilization protocol described in the following section.

#### 3.2.2 Simple Protocol for Controlled Conjugate Immobilization Inside Cut Pieces of the Silica Monolith MH1 for Enzymatic Flow-Through Reactor Applications

With the methodology developed and applied, the immobilization of *de*-PG2_1000_-BAH-enzyme_*y*_ conjugates inside cut pieces of the silica monolith MH1 is based on the simple adsorption of the conjugates from added aqueous incubation solutions containing the conjugates. As mentioned above (see section 3.2.1), the conjugate concentration in the incubation solutions was kept below 80 μM r.u., i.e., below the estimated concentration for the saturation of the internal surface by the conjugates ([r.u.]_max_). This means, that for the conjugates prepared and used in this work (see Table S1) the highest concentrations of active enzyme molecules that could be used in the conjugate incubation solutions were 3.2 μM HRP and 7.1 μM BCA. Since [r.u.]_max_ = 80 μM was estimated defensively, the actual maximal loading may be higher. However, the maximal loading of the monolith with conjugates was not the target of this work. In contrast, the aim was to ensure the *subsaturation* of the surface for denpol–enzyme conjugate immobilization as controllable and stable as possible. The key features of the immobilization protocol are described and discussed in the following sections. For experimental details, see section 2.6.

The enzymatic flow-through reactors were assembled from cut pieces of MH1 rods inserted into LDPE tubing (see chapter 12 of the Supporting Information for details). The tight embedding in simple tubing is quick and cheap and allows for an arbitrary choice of monolith length. Solutions that flow through the tortuous channels of the monolith are expected to be mixed radially on their way through the monolith for the typical flow rate applied in this work (200 μL min^–1^, corresponding to 1.6 mL min^–1^ cm^–2^ at *d*_m_ ≈ 4 mm). This cross-sectional flow was similar to the one used by van der Helm et al.^60^ for the same type of monolith (≈0.3–1 mL min^–1^ cm^–2^). Based on previous investigations,^45^ the expectation is that the flow through the MH1 channels under the conditions used will be homogeneous (low flow hindrance). This is important if one aims to control and correctly interpret the observed product formation (under initial reaction rate conditions) for substrate solutions passed through the monolith pieces containing immobilized enzymes.

The enzyme immobilization steps are depicted in Figure 1. Conjugate incubation solutions of the same volume as the pore volume of the cut monolith piece were added to the monolith piece (50 μL for *l*_m_ = 5 mm). The entire solution volume was sucked up by the monolith due to capillary forces. This monolith piece was then incubated at RT for 3 h to allow for conjugate adsorption. The concentration of active enzyme molecules in the conjugate incubation solution was varied between [HRP] = 50 and 500 nM or [BCA] = 1.0 and 5.1 μM by simple diluting the respective conjugate stock solutions (see section 2.6.2). After incubation with the conjugate incubation solution, the loaded monolith pieces were washed thoroughly with PBS (overnight at 200 μL min^–1^), and the small amounts of enzyme (if any) that were washed out were quantified (see section 2.6.5, chapters 13 and 17 of the Supporting Information, and Figure S17).

The *enzyme immobilization yield* was determined by comparing the amount of enzyme that was added to the monolith piece with the amount of enzyme that remained inside the monolith piece after it was washed with PBS. For all conjugate incubation solutions used, the immobilization was highly reproducible, with immobilization yields between 97 and 100% (see chapter 5 of the Supporting Information and Table S2). This indicates an extremely efficient use of the prepared denpol–enzyme conjugates, with almost no waste.

### 3.3 Flow-Through Activity of Enzyme Reactors

#### 3.3.1 Controllable Enzyme Activity upon the (Co-)Immobilization of Defined Amounts of Enzyme Molecules

**Figure 5:**
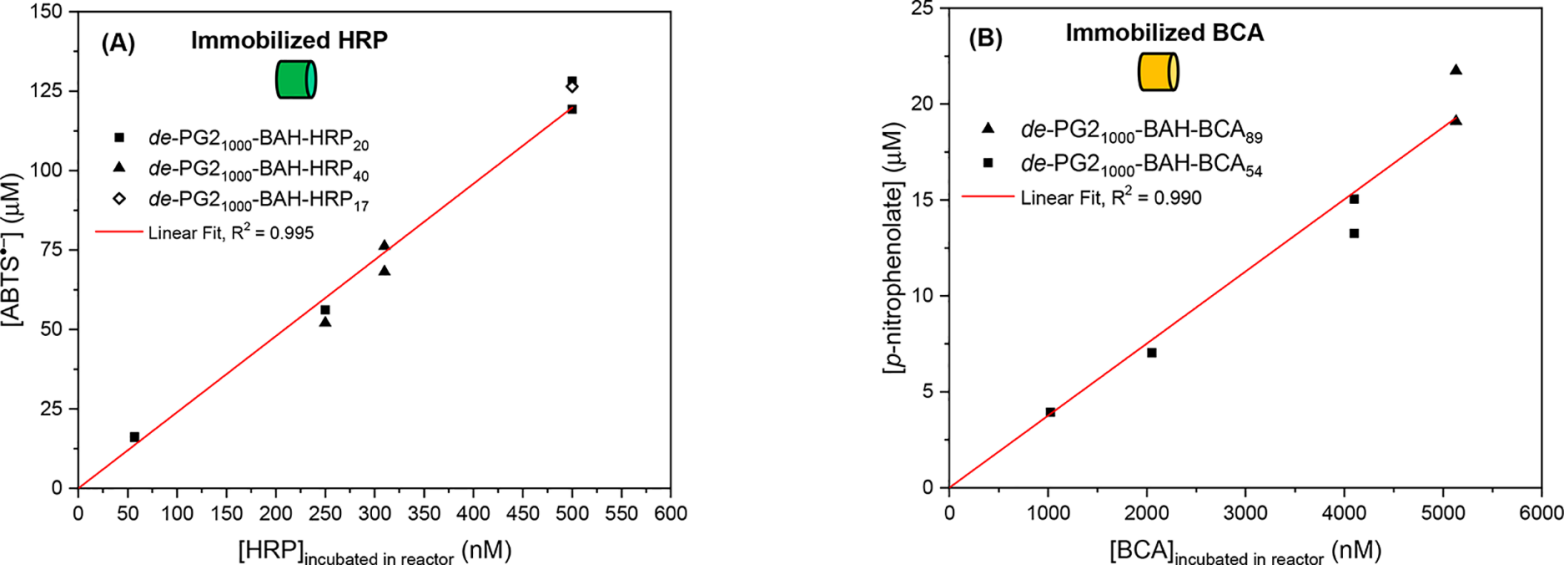
Proportionality between the measured reaction product concentration present under steady-state conditions (after *t* = 20 min) in the outflows from (A) HRP or (B) BCA reactors and the concentration of the enzyme in the conjugate incubation solutions used for the preparation of the enzyme reactors. All enzyme reactors were built from monolith pieces of the same size (*d*_m_ ≈ 4 mm and *l*_m_ = 5 mm), and the volume of the added conjugate incubation solution was always 50 μL (*V*_L_). The flow rate at which the substrate solutions were pumped through the enzyme reactors was 200 μL min^–1^ in all cases; see Figure 2a and b. In panel A, the substrate solution for the HRP reactors is as follows: 1.0 mM ABTS^2–^ and 0.2 mM H_2_O_2_, pH = 7.2 (PBS). The product analyzed was ABTS^•–^ (at λ = 414 nm). The data point included for *de*-PG2_1000_-BAH-HRP_17_ (empty symbol) was taken for comparison from Hou et al.^35^ Note that there are two data points at [HRP] = 57 nM with more or less identical product concentrations in the outflow. In panel B, the substrate solution for the BCA reactors is as follows: 1.0 mM PNPA, pH = 7.2 (PB). The product analyzed was *p*-nitrophenolate (at λ = 405 nm). For time vs reaction product concentration profiles of the measured enzyme reactors, see the Supporting Information, Figures S18 and S19.

As shown above, the strength of our immobilization method is that the number of immobilized enzymes in the monolith piece is determined—and therefore controlled—by the enzyme concentration in the conjugate incubation solution used. With this, various enzyme reactors were prepared using incubation solutions of *de*-PG2_1000_-BAH-HRP_40_, *de*-PG2_1000_-BAH-HRP_20_, *de*-PG2_1000_-BAH-BCA_54_, or *de*-PG2_1000_-BAH-BCA_89_. The length of the monolith piece usually was *l*_m_ = 5 mm, with *d*_m_ ≈ 4 mm and *V*_L_ = 50 μL (see Figure 2a and b). The activities of the enzymes immobilized inside the monolith piece were measured in continuous flow-through mode at a flow rate of 200 μL min^–1^ using either 1.0 mM ABTS^2–^/0.2 mM H_2_O_2_ and PBS (pH = 7.2) (for HRP) or 1.0 mM PNPA and PB (pH = 7.2) (for BCA) as substrate solutions (see sections 2.8.2. and 2.8.3, chapter 18 of the Supporting Information and Figures S18–S20). When the steady-state activities of the enzyme reactors under flow-through conditions (determined as initial reaction rates) were compared to the amount of conjugated enzyme in the conjugate incubation solutions used to prepare the reactors, a linear proportionality was found (see Figure 5). The reproducibility of the measurements was high and independent of the type of enzyme or average number of enzyme molecules per conjugate. The data in Figure 5, which also include data for a HRP conjugate prepared and immobilized in MH1 in our previous work (empty diamond),^35^ show not only that reproducible, controlled enzyme immobilization in a monolith piece is possible but also that the simple method we developed is rather robust. Using (i) a monolith piece with twice the length of the monoliths used in the measurements shown in Figure 5 (*l*_m_ = 10 instead of 5 mm), (ii) the same conjugate incubation solution for the immobilization of the enzymes as that in the case of the measurements shown in Figure 5 (but *V*_L_ = 100 instead of 50 μL), (iii) the same substrate solution pumped through the prepared enzyme reactors, and (iv) the same flow rate (200 μL min^–1^, high enough for initial rate conditions), the amount of product obtained at steady-state was doubled due to a doubling of the residence time (τ) (see chapter 19 of the Supporting Information and Figures S21 and S22). In addition, if *the same amount* of conjugates was distributed within one monolith piece of length *l*_m_ = 5 mm or within two monolith pieces (each again of length *l*_m_ = 5 mm), *the same product formation* in flow-through operation was observed (compare the one reactor (1*x*) with [HRP]_incubated_ = 500 nM in Figure S18 with 2[HRP]_incubated_ = 250 nM in Figure S22). With such simple control of the enzyme concentration in the conjugate incubation solution and the choice of a desired length of the monolith piece, it is possible to predetermine the extent of the reaction product formation per time unit in a flow-through device. Such a predetermination of the rate of product formation should be possible for any type of enzymatic flow-through reactor if the immobilization of the enzyme can be done in a highly controlled manner, for example, using the His-tag or fusion protein approaches.^12,13,24^ The predetermination of a defined amount of immobilized enzymes is an important requirement for the targeted controlled *coimmobilization* of *de*-PG2_1000_-BAH-HRP_40_ or *de*-PG2_1000_-BAH-BCA_89_.

**Figure 6:**
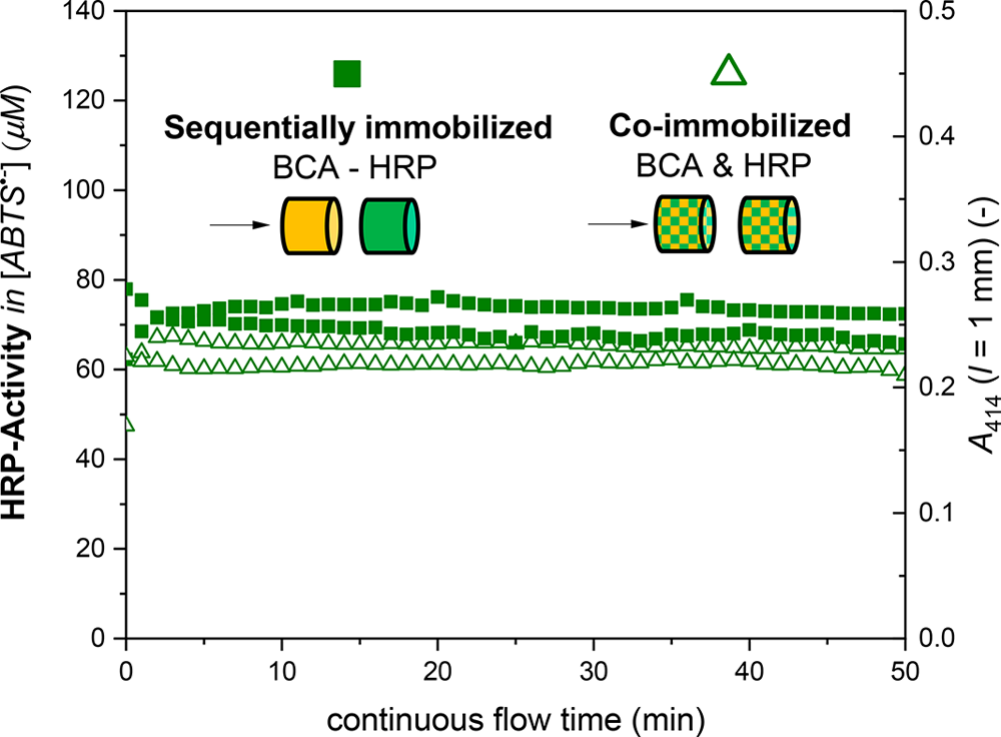
Flow-through analysis of the activity of HRP (no cascade reaction) in two two-enzyme reactor systems. The first one consisted of a BCA reactor connected to a HRP reactor. In the second system, two reactors consisting of BCA and HRP were connected; see Figure 2c and d. Schematic drawings of the two enzyme reactor systems that were placed inside the LDPE tubing (not shown) are given, with arrows indicating the direction of the continuous flow of the substrate solution for 50 min at 200 μL min^–1^. The substrate solution is as follows: 1.0 mM ABTS^2–^ and 0.2 mM H_2_O_2_, pH = 7.2 (PBS), at RT. All four monolith pieces had the same size (*d*_m_ ≈ 4 mm and *l*_m_ = 5 mm) and were loaded with the same volume of conjugate incubation solutions (*V*_L_ = 50 μL). The conjugates used were *de*-PG2_1000_-BAH-BCA_89_ and *de*-PG2_1000_-BAH-HRP_40_. The enzyme concentrations in the conjugate incubation solutions were [BCA] = 5.1 μM and [HRP] = 310 nM in the case of sequential enzyme immobilization and [BCA] = 2.55 μM and [HRP] = 155 nM in the case of enzyme coimmobilization. The concentration of the reaction product ABTS^•–^ in the outflow from the reactor systems is plotted vs the flow time.

Regarding the satisfying results presented so far, the only additional condition to be met for the coimmobilization of the two enzymes was that the two conjugates do not influence each other’s adsorption in a negative manner (or do not impact the activity assays used for their quantification). Therefore, the following experiments were carried out. Incubation solutions of the two conjugates were added to two monolith pieces of the same length and diameter, either together (i.e., they were *coimmobilized* in both monolith pieces) or individually (i.e., they were immobilized separately, one conjugate type per monolith piece, with a *sequential* connection of the two monolith pieces, BCA first and HRP second; see Figure 2d and c, respectively). The total number of enzyme molecules incubated was the same for each enzyme in both experiments (256.5 pmol active BCA and 15.5 pmol active HRP, see sections 2.6.3 and 2.6.4, respectively). As shown in Figure 6, the HRP activity (determined with ABTS^2–^ and H_2_O_2_ as substrates) was about the same for both setups. The same was the case for BCA (determined with PNPA; see Figure S21). Therefore, for the conditions used, it seems that the two conjugates did not disturb each other during adsorption and their quantification was not affected. Obviously, there was both enough internal monolith surface onto which both conjugates could adsorb and enough time provided for the conjugates to adsorb (3 h), so that there was no effect from possible differences in conjugate adsorption kinetics.

#### 3.3.2 “Activity Recovery” upon Immobilization

There are several methods of quantifying and comparing the activities of immobilized enzymes in a flow-through device with the activities of the enzyme molecules used for the immobilization. At some point one is forced to compare the “performance” of the same enzyme in two completely different situations (environments), that is, immobilized and in bulk solution.^1,2^ Therefore, such qa uantitative comparison is not trivial. It depends not only on how efficient the immobilization of the enzyme is but also on how the immobilized enzyme behaves under flow with respect to a comparable behavior in solution. Applying the terminology described by Sheldon and van Pelt^1^ to our system, “activity recovery” is defined as the product of (i) the enzyme immobilization yield (see section 3.2.2) and (ii) the enzyme immobilization efficiency, i.e., the activity of the immobilized enzyme measured with a flow-through assay under certain flow-through conditions in comparison to the activity of the enzyme measured with the same assay in the bulk solution. Determined values of “activity recovery” are listed in Table 1. Since the enzyme immobilization yield we used for the enzyme immobilization method was always nearly 100% (all denpol–enzyme conjugates present in the conjugate incubation solution adsorbed inside the monolith piece), the determined assay-dependent activity recovery (30–60%) is the actual enzyme immobilization efficiency. While being reproducible for flow-through reactors containing different amounts of immobilized enzyme molecules, the activity recovery for equal reactors was found to be dependent on the assay conditions used. Therefore, the enzyme immobilization efficiency also depends on the type of substrate and other experimental conditions used during the flow-through assay, not just on the immobilization method as such.^1^ See also chapter 20 of the Supporting Information and Tables S3 and S4.

**Table 1:** Activity Recovery upon Immobilization of the Denpol–Enzyme Conjugates Inside Silica Monolith Pieces^a^

immobilized conjugate *de*-PG2_1000_-BAH-...	substrate (pH = 7.2)^b^	flow rate (μL min^–1^)	activity recovery^c^^,^^d^ (%)	*k*_obs_^c^ (immobilized) (s^–1^)	*k*_obs_^c^ (bulk solution) (s^–1^)
HRP_20 or 40_	1.0 mM ABTS^2–^, 0.2 mM H_2_O_2_, PBS	200	31 ± 4 (*n* = 8)	16 ± 2	51
BCA_54 or 89_	1.0 mM PNPA, PB	200	32 ± 2 (*n* = 6)	0.25 ± 0.20	0.78
HRP_40_	DCFH_2_-MA, 30 μM H_2_O_2_, PBS	5	60 ± 0 (*n* = 4)	0.027 ± 0.000	0.045
BCA_89_	50 μM DCFH_2_-DA, PBS	5	51 ± 1^e^ (*n* = 4)	-e	-e

a Determined using different flow-through assays.

b The flow-through assay conditions are given for each activity recovery determination (the composition of substrate solutions pumped through the enzyme reactors). The data for ABTS^2–^ and PNPA were taken from the measurements shown in Figure 5. The data for DCFH_2_-MA and DCFH_2_-DA were taken from measurements of the cascade reaction, see Figure 3 for reaction scheme and Supporting Information chapter 20 with Tables S3 and S4 for data. For DCFH_2_-MA, the concentration was not known, as this compound appeared as reaction intermediate.

c The activity recovery was calculated by comparing the activity observed in the flow-through enzyme reactors with that the conjugate incubation solutions used to prepare the reactors. Under initial reaction rate conditions, the activity recovery was represented by the comparison of observed rate constants, *k*_obs_ (s^–1^), which described the activity per used enzyme before and after immobilization (activity recovery = *k*_obs_(immobilized)/*k*_obs_(bulk solution)). See Supporting Information chapter 20 for details about the determination of *k*_obs_ and sections 2.3.1 and 2.3.2 and Supporting Information chapter 24 for details about the bulk solution assays.

d Mean values and standard deviations are given; *n*, number of enzyme reactors used.

e Under the conditions applied to assay the BCA-catalyzed hydrolysis of DCFH_2_-DA, substrate depletion could not be ignored (conversion >20% and a substrate concentration below enzyme saturation). The activity recovery was thus calculated using first-order kinetics with respect to the decreasing substrate concentration. See Supporting Information chapter 20 for details.

#### 3.3.3 Enzyme Reactor Stability

To obtain information about the stability of the immobilized enzymes inside the monoliths, three tests were carried out. The first test was about the stability of the immobilized enzymes during the storage of the enzyme reactor under defined conditions, called “storage stability”. The second test was about the stability of the immobilized enzymes under conditions at which a buffer solution was pumped continuously through the enzyme reactor at a defined flow rate, called “stability under flow with buffer solution”. The third test was about the stability of the immobilized enzymes under the continuous conversion of substrate molecules in solutions that were pumped through the enzyme reactor at a defined flow rate, called “operational stability”. The stability tests were made with enzyme reactors, which were prepared using incubation solutions containing *de*-PG2_1000_-BAH-BCA_89_ (at [BCA] = 5.1 μM) and *de*-PG2_1000_-BAH-HRP_40_ (at [HRP] = 310 nM).

##### 3.3.3.1 Storage Stability

**Figure 7:**
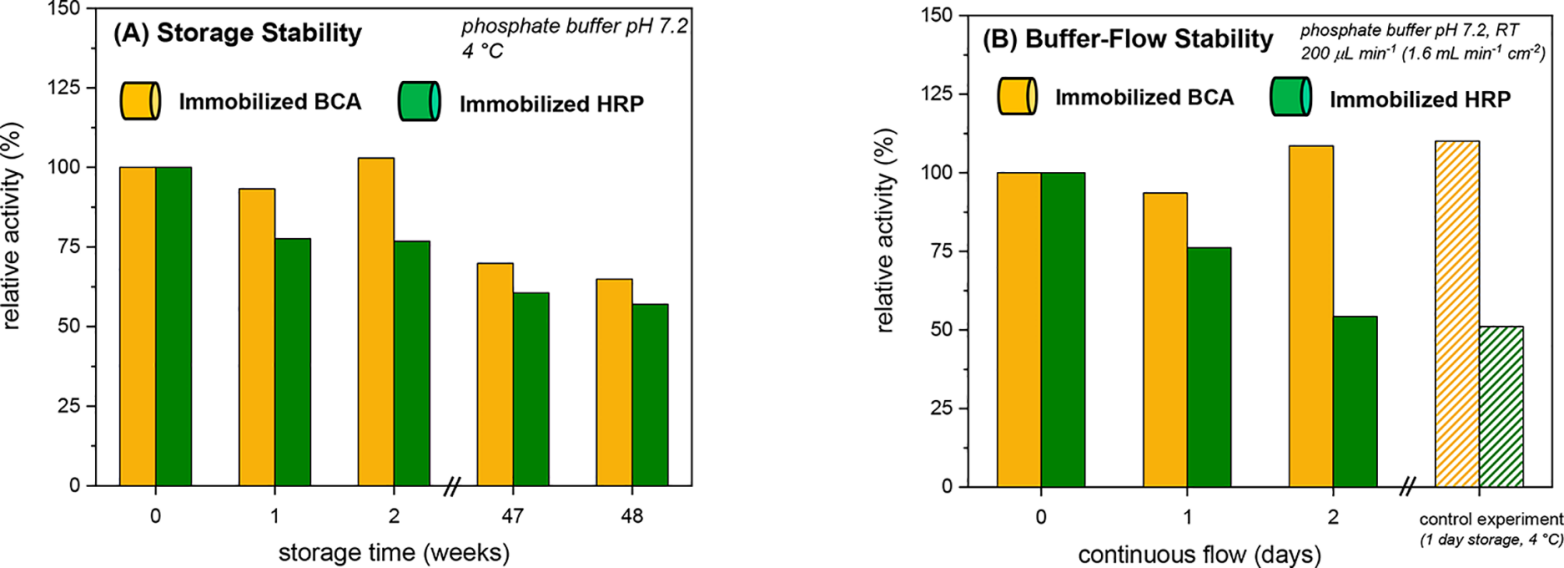
(A) Storage stability and (B) stability under flow with a buffer solution of BCA and HRP reactors. The enzyme reactors were prepared using conjugate incubation solutions containing either *de*-PG2_1000_-BAH-BCA_89_ at [BCA] = 5.1 μM and *de*-PG2_1000_-BAH-HRP_40_ at [HRP] = 310 nM or *de*-PG2_1000_-BAH-BCA_89_ at [BCA] = 5.1 μM and *de*-PG2_1000_-BAH-HRP_20_ at [HRP] = 500 nM for the experiments shown in panels A and B, respectively. In panel A, the enzyme reactors were stored at 4 °C, filled with PBS, and analyzed after the indicated storage time by passing substrate solutions of the following compositions through the reactors at a rate of 200 μL min^–1^ at RT: for the HRP reactor, 1.0 mM ABTS^2–^ and 0.2 mM H_2_O_2_, pH = 7.2 (PBS); for the BCA reactor, 1.0 mM PNPA, pH = 7.2 (PB). After the indicated period of storage, the product concentration—[ABTS^•–^] for HRP or [*p*-nitrophenolate] and [*p*-nitrophenol] for BCA—was determined in the outflows under steady-state conditions (after 20 min) and compared to the product concentration measured in the outflow for the same enzyme reactor before storage (set to 100%). In panel B, the enzyme reactors were exposed to a continuous flow of PBS at 200 μL min^–1^ at RT. At the beginning, after 1 and 2 days, the same substrate solutions mentioned for panel A were pumped through the reactors at a rate of 200 μL min^–1^, and the product concentrations in the outflows were determined under steady-state conditions (after 20 min). The last two dashed panels in panel B refer to control experiments in which the two enzyme reactors that were first exposed to a continuous flow with PBS over 2 days were stored at 4 °C for 1 day without flow before the enzyme activity was measured in the same way as described above.

Immediately after the two enzyme reactors were prepared, they were assayed (for 50 min at 200 μL min^–1^) with their respective substrate solutions (ABTS^2–^/H_2_O_2_ and PBS (pH = 7.2) for HRP, PNPA and PB (pH = 7.2) for BCA) and then stored immersed in PBS (pH = 7.2) at 4 °C. The activity measurements were repeated after 1, 2, 47, and 48 weeks of storage. The measured residual activity is plotted in Figure 7A. The BCA reactor fully retained its activity for the first two weeks after preparation and then dropped to about 70% its initial value after 47–48 weeks. For the HRP reactor, the HRP activity decreased to 75% after one week and retained this activity during the second week of storage. After 47–48 weeks, the HRP activity was still at about 60% the initial value. A similar storage stability behavior of an immobilized denpol–HRP conjugate was previously observed for *de*-PG2_1400_-BAH-HRP_108_ adsorbed on glass coverslips (stored in phosphate buffer solution 4 °C for 10 weeks).^37^ For both monolith enzyme reactors analyzed in the present work, BCA and HRP still exhibited activities about 60% their initial values after storage at 4 °C for almost one year (Figure 7). In the case of HRP, one possible reason for the decrease of ≈25% during the first week of storage could be that H_2_O_2_-mediated inactivation of some of the HRP molecules occurred over time after an initial contact of the immobilized HRP molecules with H_2_O_2_ (used for the first activity measurement with ABTS^2–^).^61^ This is, however, pure speculation.

##### 3.3.3.2 Stability under Flow with a Buffer Solution

The mechanical impact of continuously passing PBS at RT through a monolith piece containing immobilized HRP or BCA was tested at a flow rate of 200 μL min^–1^ (corresponding to 1.6 mL min^–1^ cm^–2^) for a duration of two days. The remaining activity of the immobilized enzymes was measured immediately after the preparation of the enzyme reactor, after 24 h, and after 48 h by pumping substrate solutions (instead of PBS) through the enzyme reactors for a period of 20 min at the same flow rate (200 μL min^–1^) and monitoring the substrate conversion. The results obtained are plotted in Figure 7B. While the activity of the BCA reactor remained stable, the activity of the HRP reactor dropped within two days to 50% the initial value. Compared to the BCA reactor, the HRP reactor was again less stable, with a loss of HRP activity after two days that exceeded the loss of HRP activity during storage at 4 °C without flow (Figure 7A). In a control experiment, the same two reactors were stored for one day at 4 °C after being kept under flow with PBS for two days, and the activity was determined once more. The measurements showed that there was no further decrease in HRP activity (see the panels on the right side of Figure 7B). This demonstrates that the HRP storage stability determined under flow with a buffer solution reflects the true effect of buffer flow on the HRP stability, i.e., the determination is not affected by the storage stability of HRP without flow.

The reason for the loss in HRP activity when exposed to PBS flow is not clear. What can be excluded, however, is the leakage of active HRP molecules from the monolith piece, since active HRP could not be detected in collected outflows from the monolith piece (at least with the HRP assay used and its detection limit of 5 pM HRP). This agrees with the previous observation that the physical desorption of *de*-PG2_1400_-BAH-HRP_108_ immobilized on flat silica surfaces does not occur under pH = 7.2 (PBS) conditions.^37^

##### 3.3.3.3 Operational Stability

The operational stability of one of the prepared HRP reactors was determined by continuously pumping a ABTS^2–^ (1.0 mM)/H_2_O_2_ (0.2 mM) solution (pH = 7.2, PBS) through a monolith piece containing immobilized *de*-PG2_1000_-BAH-HRP_20_ at a rate of 200 μL min^–1^ for 48 h at RT (see chapter 21 of the Supporting Information and Figure S23). Before this experiment was started, the HRP reactor was filled with PBS and then stored at 4 °C for two weeks for “HRP activity equilibration” (the change in HRP reactor activity during the first two weeks of storage at 4 °C is shown in Figure 7A). As shown in Figure S23, during continuous flow with the substrate solution, the activity of the HRP reactor decreased to 50% its initial value after 24 h and to 25% its initial value after 48 h. Therefore, there was a significant loss in HRP reactor activity under continued substrate oxidation. Due to the high flow rate with which the substrate solution was pumped through the monolith (200 μL min^–1^), the achieved substrate conversion was relatively low, corresponding to initial reaction rate conditions. Earlier operational stability determinations of a monolith that was loaded with a previously prepared *de*-PG2_1000_-BAH-HRP_70_ conjugate, again using a ABTS^2–^/H_2_O_2_ substrate solution of the same composition, indicated no decrease in activity after 10 h.^35^ The difference between the results obtained previously and the present work originates from the significantly reduced flow rate in the previous work (35 μL min^–1^ as compared to 200 μL min^–1^ in the present work, which was accompanied by a higher enzyme loading of 2 μM versus 310 nM in the present work),^35^ which resulted in higher substrate conversion. For enzymatic assays in general, the reaction product formation can usually be considered to be linearly proportional to the concentration of the active enzyme for substrate conversions below 20%.^59^ When measuring single points on the reaction progression curve (as one is mostly forced to do in flow-through assays), using a too long reaction time significantly decreases the range within which a direct correlation exists between the measured amount of reaction product formed and the amount of the active enzyme.^58,59,62^ As a consequence, at high substrate conversions, two similar measured product concentrations might originate from two very different concentrations of active enzyme; for an example, see Figure 1.5 in the work of Bisswanger.^58^ This effect of substrate depletion on the measured reaction rate is particularly relevant for surface-immobilized enzymes.^63,64^ For immobilized enzymes that are assayed at a high substrate conversion, a high retention of the reaction product concentration in the outflow may not be due to a high stability of the immobilized enzymes. For operational stability data that directly reflect the *true* stability of the immobilized enzyme during substrate conversion, flow conditions must be applied that yield *low substrate conversions* (<20%). For practical synthetic applications of flow-through enzyme reactors, however, high substrate conversion is usually desired.

Operational stability measurements of a BCA reactor that was run for the same time period as that in the case of the HRP reactor (48 h) were not possible due to the high rate of the autohydrolysis of PNPA in the 10 mM phosphate buffer solution at pH = 7.2 (PB). However, the analysis showed that the prepared BCA reactor was stable during continuous substrate conversion for at least 50 min (see Figures S18 and Figure S19).

### 3.4 Analysis of an Enzymatic Cascade Reaction in Both Bulk Solution and Flow-Through Reactors Containing Immobilized BCA and HRP

As demonstrated above using BCA and HRP as model enzymes, the strength of the method we developed for the immobilization of enzymes inside the porous silica monolith MH1 is the *high control* over the amount of immobilized enzyme molecules. BCA and HRP were chosen in this work because in one of our previous investigations we showed that the two enzymes could be used to catalyze a two-enzyme cascade reaction (see Figure 3 and Ghéczy et al.^42^). In that previous work, this cascade reaction was studied in great detail with either BCA and HRP dissolved in bulk solution or the two enzymes immobilized in glass fiber filters for flow-through applications.^42^ In the present work, the same cascade reaction was applied with both enzymes immobilized at defined amounts inside MH1 monolith pieces, either sequentially or together (coimmobilized) (Figure 2), and the performances of the prepared enzyme reactors were analyzed. The focus was on answering the question of whether there would be a benefit to using enzyme coimmobilization over sequential enzyme immobilization. Compared to the previous experiments with BCA and HRP immobilized in glass fiber filters as support materials,^42^ the use of the monolith MH1 has clear advantages in terms of reproducibility, the control and efficiency (activity recovery) of enzyme immobilization, the adjustment of enzyme reactor length, and the general ease of enzyme reactor handling.

**Figure 8:**
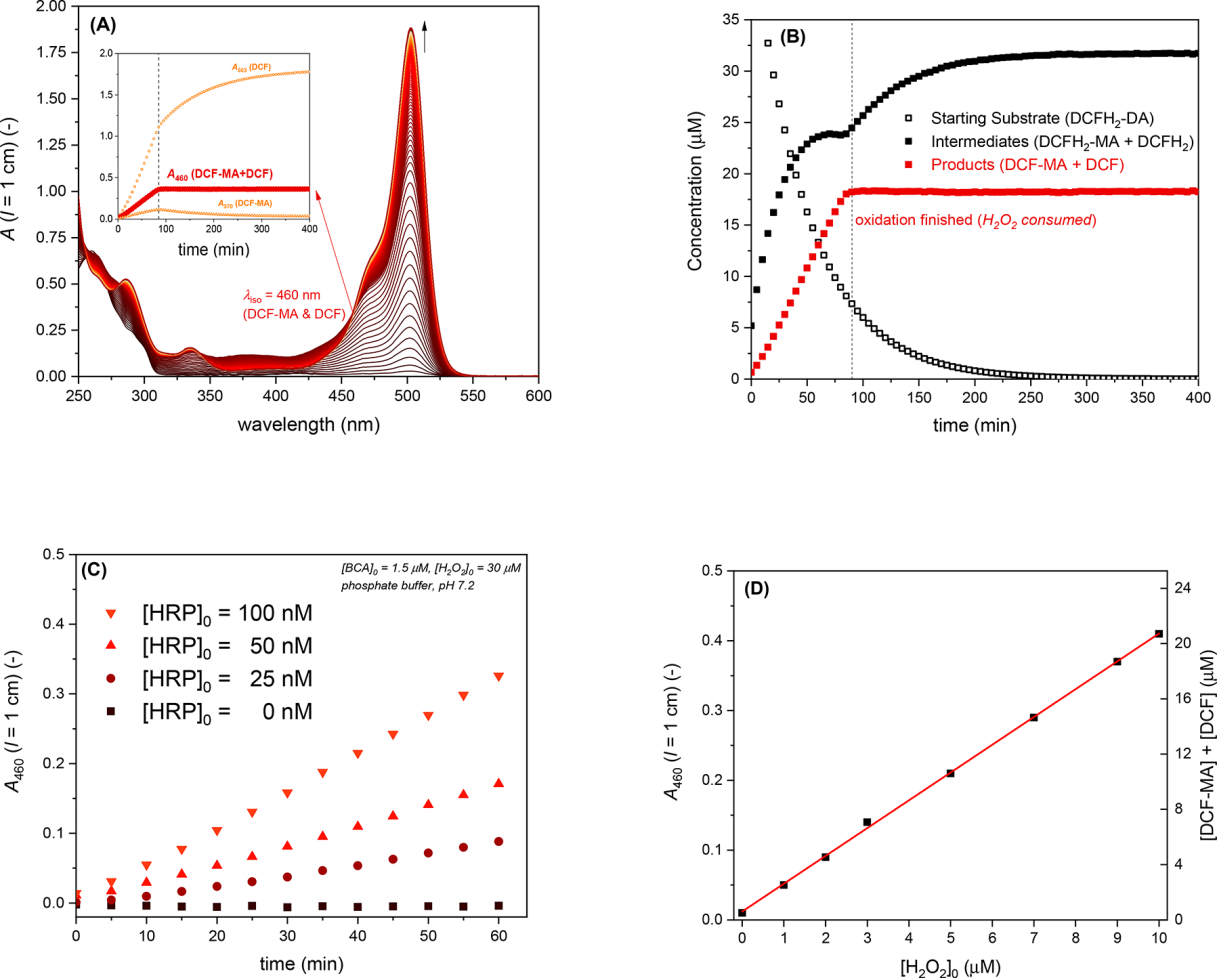
Reference measurements of the two-enzyme cascade reaction shown in Figure 3 run in the bulk solution inside quartz glass cuvettes at RT with the two enzymes, namely, BCA and HRP, and the substrates DCFH_2_-DA and H_2_O_2_, all of which were present from the beginning at [BCA] = 1.5 μM and [DCFH_2_-DA]_0_ = 50 μM, pH = 7.2 (PBS). The conditions were chosen such that the reaction proceeded predominantly over *pathway 2* (see Figure 3). (A) UV–vis spectra of the reaction mixture recorded during the reaction (every 5 min for 15 h) using [HRP] = 100 nM and [H_2_O_2_]_0_ = 10 μM. In the inset, *A*_460_ (red) indicates the formation of DCF-MA or DCF (absorbance at the isosbestic point of the two molecules) and *A*_503_ (orange) is the absorbance at λ_max_ of DCF. The change in *A*_370_ reflects changes in [DCF-MA].^42^ After 90 min, *A*_460_ (DCF-MA and DCF) remained stable, while *A*_370_ (DCF-MA) decayed exponentially and *A*_503_ (DCF) continued to increase due to *Hyd_3* (see Figure 3). Since *A*_460_ remained stable above *t* = 90 min, oxidation no longer took place, i.e., all the H_2_O_2_ was used up. (B) Changes in the molar concentrations of DCFH_2_-DA, DCFH_2_-MA and DCFH_2_, and DCF-MA and DCF for the reaction shown in panel A. Changes were determined using ε_460_(DCF-MA/DCF) = 19 800 M^–1^cm^–1^ (see Ghéczy et al.^42^) and *k*_cat_/*K*_M_ = 0.0130 μM^–1^ min^–1^ for the BCA-catalyzed hydrolysis of DCFH_2_-DA (see the Supporting Information, Figure S24) and by considering the mass balance to determine [DCFH_2_-MA] + [DCFH_2_] = 50 μM – ([DCFH_2_-DA] + [DCF-MA] + [DCF]). The calculated concentrations were crosschecked by spectral fitting the spectrum at *t* = 90 min (using the molar absorptions for all reaction components as obtained in our previous work).^42^ (C) Dependence of the initial rate of formation of DCF-MA and DCF on the concentration of HRP for [H_2_O_2_]_0_ = 30 μM, as analyzed by recording the increase in *A*_460_ with time (see the Supporting Information, Figure S26, for details). (D) Dependence of the formation of DCF-MA and DCF on the initial concentration of H_2_O_2_ (between 1 and 10 μM) for [HRP] = 100 nM, as analyzed by recording *A*_460_ after *t* = 2 h. The ordinate on the right side refers to the total concentration of [DCF-MA] and [DCF], which was calculated by taking into account ε_460_(DCF-MA/DCF) = 19 800 M^–1^cm^–1^ (see Ghéczy et al.^42^). As expected for the peroxidase cycle of HRP,^50^ for each two electron reduction of H_2_O_2_ by HRP, two one-electron oxidation products are produced. The solid line (red) reflects the linear regression of [DCF-MA] + [DCF] = 0.61 μM + 2.01 × [H_2_O_2_]_0_ (*R*^2^ = 0.9994, see the Supporting Information, Figure S30, for details).

The cascade reaction system shown in Figure 3 is somewhat unique because it consists of two possible reaction pathways involving BCA and HRP-catalyzed reaction steps. For both pathways, the added substrate DCFH_2_-DA (2′,7′-dichlorodihydrofluorescein diacetate) is initially hydrolyzed to DCFH_2_-MA (2′,7′-dichlorodihydrofluorescein monoacetate), which is catalyzed by BCA. In the case of *pathway 1*, a second BCA-catalyzed hydrolysis reaction occurs first and yields DCFH_2_ (2′,7′-dichlorodihydrofluorescein), which is then oxidized to DCF (2′,7′-dichlorofluorescein) in a reaction catalyzed by HRP in the presence of added H_2_O_2_. DCF has an absorption maximum at λ = 503 nm with ε_503_(DCF, pH = 7.2) = 109 000 M^–1^cm^–1^ (see Ghéczy et al.^42^). In the case of *pathway 2*, DCFH_2_-MA is first oxidized with HRP and H_2_O_2_ to DCF-MA (2′,7′-dichlorofluorescein monoacetate), which is then hydrolyzed to DCF (see Ghéczy et al.^42^ for details). For both pathways, the “final” product is DCF. The “beauty” of this model cascade reaction is that simple spectrophotometric measurements of the entire reaction mixture allow the quantitative determination of the concentrations of DCFH_2_-DA, DCFH_2_-MA, DCF-MA, DCFH_2_, and DCF during the course of the reaction.^42^ Please note that the possible oxidation of DCF by HRP/H_2_O_2_ is omitted in Figure 3, since this oxidation step can be prevented by avoiding the use of excess HRP.^42^ Moreover, in a bulk solution in which BCA, HRP, DCFH_2_-DA, and H_2_O_2_ are present from the beginning, the concentrations of HRP and BCA and the molar ratio of HRP to BCA determine the contributions of *pathway 1* and *pathway 2* to the overall cascade reaction system.^42^ With a very high ratio of [BCA] to [HRP], the reaction is expected to proceed mainly via *pathway 1*. With a low enough ratio of [BCA] to [HRP], the reaction can proceed predominantly via *pathway 2*. In that case, the oxidation of DCFH_2_-MA to DCF-MA by HRP/H_2_O_2_ is considerably faster than the BCA-catalyzed hydrolysis of DCFH_2_-MA to DCFH_2_. Under such conditions, considerable amounts of DCF-MA are expected to accumulate as the intermediate and then undergo relatively slow BCA-catalyzed hydrolysis to DCF until the reaction equilibrium is reached. This is the case for the bulk solution reaction conditions for which the time-dependent changes of the absorption spectrum are shown in Figure 8A and B ([DCFH_2_-DA]_0_ = 50 μM, [BCA] = 1.5 μM, [HRP] = 100 nM, and [H_2_O_2_]_0_ = 10 μM at pH = 7.2 (PBS)). Following the change in *A*_460_, i.e., the absorption at the isosbestic point for the DCF-MA/DCF pair (ε_460_ = 19 800 M^–1^cm^–1^),^42^ the increase in the total concentration of [DCF-MA] and [DCF] with time could be monitored and was shown to level off after *t* = 80 min (Figure 8A and B). After *t* = 90 min, oxidation no longer occurred because all added H_2_O_2_ molecules were consumed in the first 90 min. After *t* = 90 min, about 7 μM of the initial 50 μM DCFH_2_-DA was still present, which was then hydrolyzed to DCFH_2_-MA and DCFH_2_ over a period of 310 min (Figure 8B). The formation of the two intermediates DCFH_2_-MA and DCFH_2_ could be quantified by considering the mass balance ([DCFH_2_-DA] + [DCFH_2_-MA] + [DCF-MA] + [DCFH_2_] + [DCF] = 50 μM = [DCFH_2_-DA]_0_, see Figure 8B). The formation of DCF is indicated by the increase in *A*_503_, which clearly occurred beyond *t* = 90 min (due to the hydrolysis of DCF-MA, see Figure 8A). After *t* = 400 min, neither DCFH_2_-DA nor H_2_O_2_ was present, [DCF] = 17.5 μM, [DCF-MA] = 0.8 μM, and [DCFH_2_-MA] + [DCFH_2_] = 31.7 μM. For the same initial concentrations of DCFH_2_-DA and BCA, [DCFH_2_-DA]_0_ (50 μM), [BCA] (1.5 μM), and [H_2_O_2_]_0_ = 30 μM, the rate of DCF-MA and DCF formation was proportional to the concentration of HRP (for 25, 50, and 100 nM HRP; see Figure 8C). Moreover, for [DCFH_2_-DA]_0_ = 50 μM, [BCA] = 1.5 μM, and [HRP] = 100 nM, the amount of DCF-MA and DCF formed was linearly dependent on [H_2_O_2_]_0_ (for 1–10 μM H_2_O_2_, which was added as last component to the reaction mixture; see Figure 8D). Analysis of additional experimental data showed that the BCA-catalyzed hydrolysis of DCFH_2_-DA occurred faster (*k*_cat_/*K*_M_ = 0.130 μM^–1^ min^–1^) than the BCA-catalyzed hydrolysis of DCFH_2_-MA (*k*_cat_/*K*_M_ = 0.017 μM^–1^ min^–1^). See chapters 23–27 of the Supporting Information, Table S5, and Figures S24–S31. This is in agreement with the reaction proceeding mainly via *pathway 2* for the conditions used.

After some reference measurements were carried out in the bulk solution (Figure 8), the cascade reaction was then applied to the enzyme reactors for a flow-through analysis. The two enzymes BCA and HRP were immobilized using the denpol–enzyme conjugates *de*-PG2_1000_-BAH-BCA_89_ and *de*-PG2_1000_-BAH-HRP_40_. The conjugates were either coimmobilized in two identical monolith pieces (*l*_m_ = 5 mm, *d*_m_ ≈ 4 mm and *V*_L_ = 50 μL each) or immobilized individually in two sequentially connected monolith pieces of the same size, see Figure 2c or d, respetively. For both setups, the same amount of each enzyme was used for the enzyme reactor preparation. In the case of the BCA conjugate, [BCA] in the conjugate incubation solution was 5.1 μM for BCA immobilization in one monolith piece and 2.55 μM for coimmobilization in two monolith pieces. In the case of the HRP conjugate, the concentrations in the conjugate incubation solutions were 310 and 155 nM HRP, respectively (see sections 2.6.3 and 2.6.1). The two enzyme reactors with coimmobilized enzymes resembled the situation in the bulk solution measurements, since the reaction mixture was exposed to both enzymes simultaneously.

**Figure 9:**
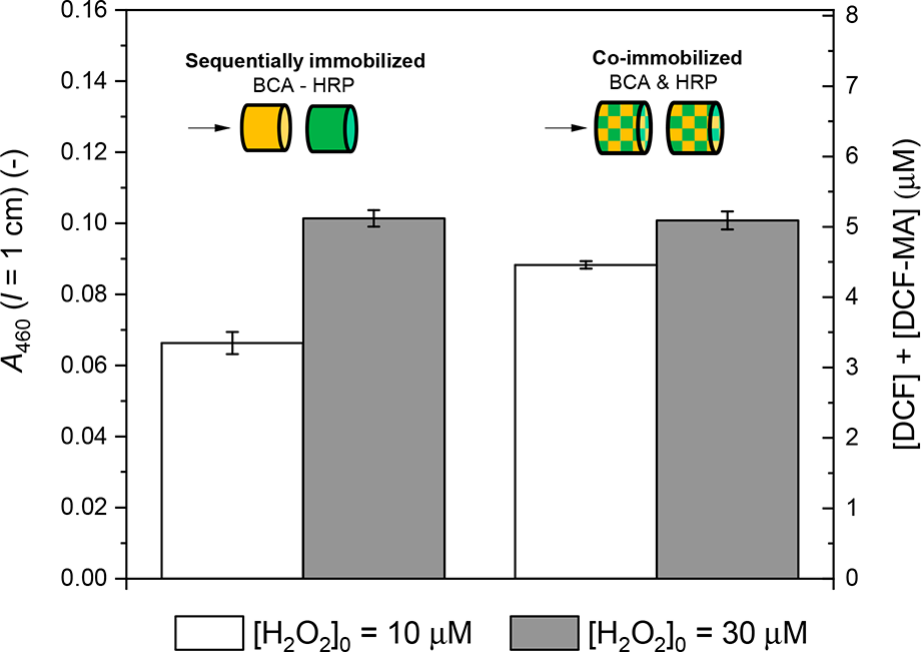
Analysis of the two-enzyme cascade reaction shown in Figure 3 run in the two enzyme reactor types shown in Figure 2c and d; BCA and HRP were either sequentially immobilized or coimmobilized. The denpol–enzyme conjugates used were *de*-PG2_1000_-BAH-BCA_89_ and *de*-PG2_1000_-BAH-HRP_40_ (see sections 2.6.3 and 2.6.4 and Figure 6). An aqueous substrate solution consisting of [DCFH_2_-DA]_0_ = 50 μM and [H_2_O_2_]_0_ = 30 (filled bar) or 10 μM (empty bar) in PBS (pH = 7.2) was pumped through the enzyme reactor systems at 5 μL min^–1^ for 5 h. The absorption spectrum of the outflow from the reactors was measured under steady-state conditions (after 4.0, 4.5, and 5.0 h), and *A*_460_ was taken as measurement of the reactor performance (formation of DCF-MA and DCF). At [H_2_O_2_]_0_ = 30 μM, the formation of DCF-MA and DCF was limited by the HRP exposure (amount of immobilized active HRP and residence time, τ). Thus, both reactor systems showed the same product formation. At [H_2_O_2_]_0_ = 10 μM, the formation of DCF-MA and DCF was limited by H_2_*O*_2_ (at least for the sequential setup, see Figure 10). Due to a more efficient use of the otherwise scarce H_2_O_2_, the coimmobilized enzymes showed higher product formation (see also Figure S33).

Two different substrate solutions were pumped through the enzyme reactors at a flow rate of 5 μL min^–1^. Both substrate solutions contained 50 μM DCFH_2_-DA and either 10 or 30 μM H_2_O_2_ in PBS at pH = 7.2 (see section 2.8.4). The outflows from the monolith pieces were collected every 30 min, and the UV–vis absorption spectrum of each pooled outflow sample was recorded (see chapter 28 of the Supporting Information, and Figure S32). The formation of DCF-MA and DCF was monitored by plotting *A*_460_ versus the time during which the substrate solution was pumped through the two monolith pieces. After about 4 h of continuous flow, *A*_460_ reached a stable value, indicating the beginning of steady-state product formation. In Figure 9, the product formation determined for the pooled outflow samples collected between 4 and 5 h is shown for sequentially immobilized and coimmobilized enzymes using [H_2_O_2_]_0_ = 30 or 10 μM. The steady-state formation of oxidation products was the same for the two immobilization setups when 30 μM H_2_O_2_ was used ([DCF-MA] + [DCF] = 5 μM), suggesting the continuous consumption of 2.5 μM H_2_O_2_ in the oxidation of DCFH_2_-MA and DCFH_2_ (one two-electron redox reaction vs two one-electron oxidation reactions).^50^ Using a 50 μM DCFH_2_-DA solution containing 10 μM H_2_O_2_, [DCF-MA] + [DCF] in the pooled outflow samples was lower than in the case of 30 μM H_2_O_2_; for the coimmobilized enzymes the decrease was ≈10%, while for the sequentially immobilized enzymes the decrease was ≈30% (see Figure 9). To exclude a variation from the enzyme reactor or the substrate solution preparation, the same experiments were repeated with a second set of enzyme reactor preparations, confirming the observations made (see Figure S33).

**Figure 10:**
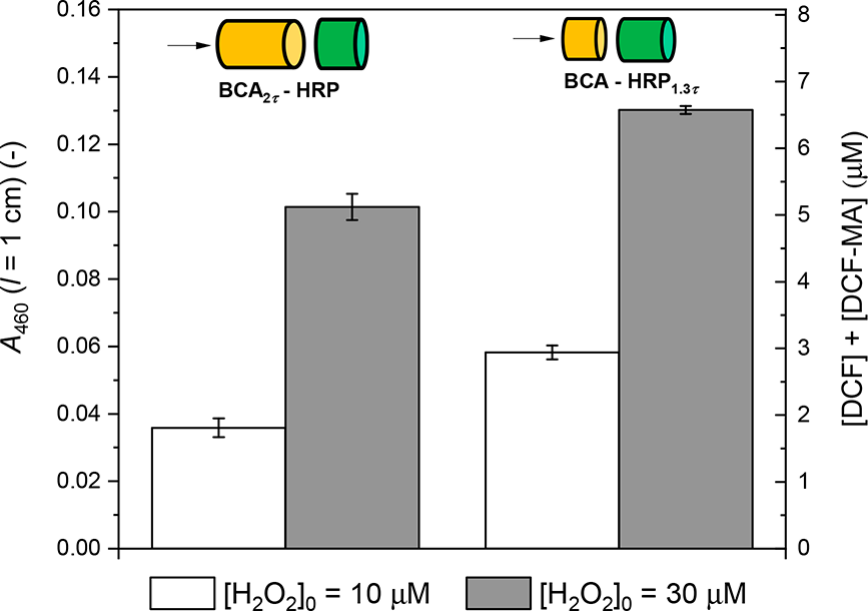
Analysis of the two-enzyme cascade reaction shown in Figure 3 run in two enzyme reactor systems where BCA and HRP were sequentially immobilized (see Figure 2c). The enzyme reactor length (i.e., the residence time, τ) was varied as indicated. The denpol–enzyme conjugates used were *de*-PG2_1000_-BAH-BCA_89_ and *de*-PG2_1000_-BAH-HRP_40_ (see section 2.6.3). An aqueous substrate solution consisting of [DCFH_2_-DA]_0_ = 50 μM and [H_2_O_2_]_0_ = 30 (filled bar) or 10 μM (empty bar) in PBS (pH = 7.2) was pumped through the enzyme reactor systems at 5 μL min^–1^ for 5 h. The absorption spectrum of the outflow from the reactors was measured under steady-state conditions (after 4.0, 4.5, and 5.0 h), and *A*_460_ was taken as measure of the reactor performance (formation of DCF-MA and DCF). At [H_2_O_2_]_0_ = 30 μM, the formation of DCF-MA and DCF was limited by the HRP exposure (amount of immobilized active HRP and residence time, τ). Thus, the longer HRP reactor showed a higher extent of product formation. At [H_2_O_2_]_0_= 10 μM, the product formation was limited by H_2_O_2_*.* Therefore, the longer HRP exposure no longer led to a higher extent of product formation. The observed decrease in the extent of product formation in the reactor system with the longer BCA reactor was likely due to nonenzymatic H_2_O_2_ decomposition within the BCA reactor *before* the substrate solution reached the HRP reactor (as observed for [H_2_O_2_]_0_ = 10 μM and low flow rate of 5 μL min^–1^ in control experiments, see the Supporting Information, Figure S36).

Additional measurements with monolith pieces of different lengths (Figures 10 and S34 and chapter 29 of the Supporting Information) clearly showed that for the chosen conditions of (i) amounts of immobilized active BCA and HRP, (ii) substrate solution composition, and (iii) flow rate the outflow composition depended on the H_2_O_2_ concentration added. This is not surprising, since H_2_O_2_ is a substrate of the cascade reaction and is used up during the course of the reaction. Using [H_2_O_2_]_0_ = 10 μM and the sequential immobilization systems, H_2_O_2_ was even the only limiting factor, which can be seen by the fact that a longer exposure to neither immobilized BCA nor immobilized HRP increased the product concentration in the outflow (compare Figure 10 to sequential immobilization in Figure 9). Moreover, for the conditions used with [H_2_O_2_]_0_ = 30 μM, the formation of DCF-MA + DCF is directly proportional to the amount of immobilized HRP, that is, HRP is rate-limiting. See also chapters 30–32 in the Supporting Information, Table S6, and Figures S35–S39.

With this set of experiments, we aimed to demonstrate the great potential of the immobilization method that we developed. It is relatively easy to load monolith pieces of a desired length with a defined amount of enzyme in a controlled way, to use a desired flow rate to pump a substrate solution of the desired composition through the monolith pieces, and to choose sequential enzyme immobilization or enzyme coimmobilization.

Depending on the conditions used, controlled enzyme coimmobilization might be more advantageous than sequential enzyme immobilization (see the data for [H_2_O_2_]_0_ = 10 μM in Figure 9). With the experiments carried out, however, there are no data that indicate an advantage of the coimmobilization of BCA and HRP in terms of a possible proximity effect (data for [H_2_O_2_]_0_ = 30 μM). In our case, the crucial H_2_O_2_ is not a reaction intermediate (which could under specific conditions profit from the molecular proximity of two enzymes)^65^ but instead an initially added cosubstrate.

## 4 Concluding Remarks and Outlook

Although we have been working for quite some time on the preparation of denpol–enzyme conjugates of the type *de*-PG2_*x*_-BAH-enzyme_*y*_ and have demonstrated their successful immobilization on silica supports,^35−39,42^ we consider the results obtained in this work to be a major step toward the use of the developed immobilization method as a potentially quite versatile^66^ and simple procedure for the preparation of highly defined enzymatic flow-through reactors, potentially for any enzyme type. Prerequisites are that it is possible to prepare a conjugate of the denpol and the enzyme of interest and that this conjugate is stable with respect to enzyme activity.

The whole concept for the controlled preparation of the enzyme reactors is based on two important “ingredients”: (i) a water-soluble polycationic polymer containing a large number of primary amines along the polymer chain, which in this work is the dendronized polymer *de*-PG2_1000_ (with an average of 4000 amines), and (ii) the macro- and mesoporous silica monolith MH1.

Concerning the monolith MH1, similar types of silica monoliths, possibly with different pore sizes,^67^ might work equally well and might be applied as well if for some reason MH1 would not satisfy the requirements for a given enzymatic reaction. In the work presented here, we always used MH1. It is the same type of silica monolith that we also used in our previous study on the immobilization of denpol–enzyme conjugates (see Hou et al.^35^). At no time was there a need to try another silica monolith. MH1 was also applied successfully to other enzyme immobilization techniques.^45,46,60^ Compared to the glass fiber filters that we also used in the past,^36,42^ MH1 has several clear advantages in terms of reproducibility, the control and efficiency (“activity recovery”) of the conjugate adsorption, the flexibility of enzyme reactor length, and the general ease of enzyme reactor handling. The disadvantage of using MH1 instead of glass fiber filters is that MH1 is currently not commercially available and must be synthesized according to the recipe described in the literature.^45^

Concerning *de*-PG2_1000_, among the denpols of a homologous series that we tested in the past,^35,55^*de*-PG2_1000_ was the denpol that was found to be ideal in terms of the average number of r.u.’s and number of branching points in each r.u. (two). Denpols of higher generations require more effort in terms of chemical synthesis.^43^ Longer denpols of the type *de*-PG2_*x*_ other than *de*-PG2_≈1000_ did not provide any obvious advantage.^35^ In theory, the noncovalent adsorption of even longer chains of the same polymer type on silica surfaces should be stronger than that in the case used because of the higher number of interactions between the polymer molecule and the surface. However, such potentially stronger adsorption was not necessary because the desorption of conjugates from the silica surface at pH = 7.2 was already insignificant for *de*-PG2_1000_ used in this work. Please note that details about the mode of interaction between the denpol–enzyme conjugates and the silica surface have not yet been explored in detail. It is quite possible that not only the many unmodified amino groups present along a single *de*-PG2_1000_-BAH-enzyme_*y*_ chain (estimated to be approximately 3700) contribute to surface adhesion but also the bound enzyme molecules (about 20–90, see section 2.2). In the latter case, enzyme molecules in direct contact with the silica support could serve as adhesion points and therefore exhibit reduced catalytic activity or be completely inactive. The denpol *de*-PG2_≈1000_ is currently not commercially available and needs to be synthesized. Protocols have been published in the literature.^39,43^

Whether *de*-PG2_1000_ could be satisfactorily replaced by a commercially available conventional polymer carrying primary amino groups that has approximately the same average degree of polymerization, for example, polylysine, is currently being investigated. Regardless of the type of polymer used, the conjugates must be prepared under sufficiently mild conditions to keep the enzymes in their active state. Fortunately, the “BAH chemistry” is not only well-established but also has the advantage of allowing the polymer molecule modification, enzyme molecule modification, and conjugate formation to be quantified spectrophotometrically. In the work presented, we have improved and simplified some of the quantifications, such as (i) the determination of the extent of HRP modification with S-4FB and (ii) the determination of the number of denpol-bound (fully active) HRP molecules. In the past there were uncertainties in the case of *de*-PG2_*x*_-BAH-HRP_*y*_ conjugates because (i) the absorption of the BAH bond at λ_max_ = 354 nm interfered with the Soret band absorption of HRP (with λ_max_ = 403 nm), (ii) HyNic may have reacted with unmodified HRP (which was discovered in this work), and (iii) the calculated BAH bond concentration (using ε_354_ (BAH) = 29 000 M^–1^cm^–1^)^39^ was only approximately consistent with the determined enzymatic activity (also observed in the case of BCA conjugates). This quantification problem has now been solved (see section 2.5).

Compared to previous studies that used flat silica surfaces to immobilize denpol–enzyme conjugates added in excess,^37,38^ the conjugate incubation solution that was added to the monolith pieces in the present work always contained conjugates at “subsaturating” concentrations. This means that essentially all conjugates present in the volume added to the monolith piece adsorbed onto the accessible inner silica surface (100% enzyme immobilization yield). Therefore, by knowing the amount of conjugate added to the monolith piece, i.e., the amount of (active) enzyme molecules in the conjugate incubation solution, the amount of immobilized enzyme molecules could be predetermined at will as long as the conjugate concentration in the conjugate incubation solution was below the saturation concentration (<80 μM r.u., as defensively estimated from the SEM analysis shown in Figure 4). In the present work, we did not push the conditions to reach the maximal enzyme loading of the monolith pieces, as this was not our aim. In previous work in which the monolith MH1 or glass micropipettes were loaded with an excess of the conjugate solution^35,37^ there was always an outflow of conjugates that did not adsorb. This required the time-consuming quantification of active enzymes in the outflow during the washing step and resulted in conjugate waste. Overall, the immobilization conditions used in the present work were basically waste-free and thus a significant improvement ecologically.

The control offered by the quantitative conjugate adsorption enabled us to investigate the efficiency of the immobilized enzymes in catalyzing the conversion of their respective substrates (in comparison to enzymes in the bulk solution, see Table 1). Applying initial reaction rate conditions within flow-through assays, not only the efficiency but also the inherent stability of the adsorbed enzymes was assessable. The stability of the immobilized enzymes was high, and leakage from the monolith pieces did not occur during operation at a relatively high flow rate (200 μL min^–1^ ≙ 1.6 mL min^–1^ cm^–2^).

Finally, the controllable enzyme immobilization inside the monolith pieces was applied to a model two-enzyme cascade reaction with DCFH_2_-DA and H_2_O_2_ as substrates. This flow-through cascade reaction is an excellent example with which to illustrate the importance of predetermining the amounts of the enzymes used for the reaction as catalysts (BCA and HRP). Only by controlling the amounts of active immobilized enzymes, a *fair* comparison can be made between individually and coimmobilized enzymes. Bulk solution experiments already indicated how the final reaction product distribution is determined by the amount of the two enzymes used and their molar ratios (see Figure 8 and Ghéczy et al.^42^). The elaborated favorable bulk solution conditions could be transferred directly to the enzyme reactor systems by means of the controlled (co)immobilization of BCA and HRP presented in this work.

Overall, the entire method is promising because it allows in a simple way—once the conjugates are prepared and tested for their stability in aqueous stock solutions—systematic studies about the behaviors of enzymes in enzyme reactors to hopefully increase our knowledge in this field further. If replacing the denpol with a simpler commercially available polymer turns out to be successful, the method could be accessed without the requirement of engaging in polymer synthesis. This would even further simplify the entire highly controllable immobilization method, hopefully making it a useful tool.

## Supporting Information Available

Experimental details, control experiments, additional considerations regarding the conjugate preparation and characterization, detailed results for the activities and stabilities of the purified conjugate stock solutions, assembly of the reactor scaffolds, additional SEM images, surface coverage calculations, raw data, determination of the activity recovery, and additional analysis (PDF)

## Abbreviations

HRP: horseradish peroxidase isoenzyme C
BCA: bovine carbonic anhydrase
PBS: 100 mM NaH_2_PO_4_ and 150 mM NaCl, pH = 7.2
PBS*: 100 mM NaH_2_PO_4_ and 1.15 M NaCl, pH = 7.2
PB: 10 mM NaH_2_PO_4_, pH = 7.2
denpol: dendronized polymer
*de*-PG2: deprotected second-generation denpol
r.u.: repeating units
S-4FB: *N*-succinimidyl 4-formylbezoate
S-HyNic: *N*-succinimidyl 6-hydrazinonicotinate acetone hydrazone
BAH: bisaryl hydrazone
MH1: the silica monolith used
ABTS^2–^(NH_4_^+^)_2_: 2,2′-azino-bis(3-ethylbenzothiazoline-6-sulfonic acid) diammonium salt
PNPA: *p*-nitrophenyl acetate
DCFH_2_-DA: 2′,7′-dichlorodihydrofluorescein diacetate
DCFH_2_-MA: 2′,7′-dichlorodihydrofluorescein monoacetate
DCFH_2_: 2′,7′-dichlorodihydrofluorescein
DCF-MA: 2′,7′-dichlorofluorescein monoacetate
DCF: 2′,7′-dichlorodihydrofluorescein
PP: polypropylene
PS: polystyrene
LDPE: low-density polyethylene
PTFE: poly(tetrafluoroethylene)
RT: room temperature
*A*_λ_: UV–vis absorbance at the wavelength λ (nm).
