# Supplementary material for: Controllable Enzyme Immobilization via Simple and Quantitative Adsorption of Dendronized Polymer–Enzyme Conjugates Inside a Silica Monolith for Enzymatic Flow-Through Reactor Applications
Source: ACS Omega. 2022 Jul 21;7(30):26610–31. doi: 10.1021/acsomega.2c02815 (PMC9352229; doi:10.1021/acsomega.2c02815)
Supplement: Supplementary file 1 — ao2c02815_si_001.pdf [file ao2c02815_si_001.pdf]

Supporting Information

Controllable Enzyme Immobilization *via* Simple and Quantitative Adsorption of Dendronized Polymer-Enzyme Conjugates Inside a Silica Monolith for Enzymatic Flow-Through Reactor Applications

Nicolas Ghéczy,[1] *Weina Xu,* [†1] *Katarzyna Szymańska,*[2] *Andrzej B. Jarzębski,*[3] *and Peter Walde*[*1]

[1] Laboratory for Multifunctional Materials, Department of Materials, ETH Zürich, Vladimir-Prelog-Weg 5, 8093 Zürich, Switzerland

[2] Department of Chemical Engineering and Process Design, Silesian University of Technology, Ks. M. Strzody 7, 44-100 Gliwice, Poland

[3] Institute of Chemical Engineering, Polish Academy of Sciences, Baltycka 5, 44-100 Gliwice, Poland

[†] Visiting student from the Key Laboratory of Industrial Biocatalysis, Ministry of Education, Department of Chemical Engineering, Tsinghua University, 100084, Beijing, China

Content

Chapter	Title	Figures, Tables	Page
1	HRP-Activity Assay in Bulk Solution with $ABTS^{2-}$ and H_2O_2 as Substrates	Fig. S1	4
2	BCA-Activity Assay in Bulk Solution with PNPA as Substrate		6
3	Visualization of the Conjugation Reaction and of the Side Reactions Between the HyNic Linker Group and Native Enzymes	Fig. S2<br>Fig. S3<br>Fig. S4<br>Fig. S5	7
4	Details about the Conjugate Preparation		12
5	Characterization of Purified Conjugate Stock Solutions	Table S1<br>Table S2	14
6	Effect of Storage and Aliquot Sampling on Enzyme Activity of Diluted Conjugate Stock Solutions	Fig. S6	16
7	Comparison of the Activity of *de*-$PG2_{1000}$-BAH-HRP_{20} to the Activity of Native HRP Using Different Substrates		18
8	Control Experiments with Mixtures of *de*-$PG2_{1000}$-$HyNic_{240}$ and Native HRP for Gaining Insights into Purified *de*-$PG2_{1000}$-BAH-HRP_y	Fig. S7	20
9	Determination of the Denpol Repeating Unit Concentration, [r.u.], in Solutions of Purified *de*-$PG2_{1000}$-BAH-HRP_y		22
10	Comments about the Conjugate Characterization		23
11	Considerations about the Yields upon Conjugate Formation and Purification		24
12	Assembly of Home-Made Flow-Through Reactor Scaffolds	Fig. S8<br>Fig. S9	25
13	Reactor Washing Protocols after Conjugate Incubation and Analysis of Enzymatic Activity Eluting from the Reactor During Washing	Fig. S10	27
14	Stability of Conjugate Stock Solutions Stored as Obtained After Purification	Fig. S11	29
15	Additional SEM Images	Fig. S12<br>Fig. S13<br>Fig. S14<br>Fig. S15<br>Fig. S16	30
16	Defensive Estimation of the Maximal Silica Surface Coverage by Conjugates *de*-$PG2_{1000}$-BAH-$enzyme_y$ as Non-Overlapping Monolayer		33
17	On the Detection of Enzyme Molecules That Leaked from the Enzyme-Reactor	Fig. S17	35
18	Flow-Through Assays of Enzyme Reactors Prepared from *de*-$PG2_{1000}$-BAH-HRP_y or *de*-$PG2_{1000}$-BAH-BCA_y	Fig. S18<br>Fig. S19<br>Fig. S20	37
19	Flow-Through Assays of Enzyme Reactors Depending on Residence Time or Enzyme Distribution	Fig. S21<br>Fig. S22	39

20	Determination of the HRP and BCA Activity Recovery upon Immobilization of *de*-PG2$_{1000}$-BAH-HRP$_y$ or *de*-PG2$_{1000}$-BAH-BCA$_y$ Conjugates in Monolith Pieces Under Flow-Through Conditions	Table S3<br>Table S4	41
21	Operational Stability of a Reactor Containing Immobilized *de*-PG2$_{1000}$-BAH-HRP$_{20}$	Fig. S23	44
22	The Applied Two-Enzymes Cascade Reaction Involving Two Reaction Pathways		45
23	BCA-Catalyzed Hydrolysis of DCFH$_2$-DA, DCFH$_2$-MA and DCF-MA at pH = 7.2	Table S5<br>Fig. S24<br>Fig. S25	46
24	Determination of the HRP-Catalyzed Rate of Oxidation of DCFH$_2$-MA in Bulk Solution by Analyzing the Cascade Reaction Proceeding Along *Pathway 2*	Fig. S26	51
25	*In Situ* Formation of DCFH$_2$ and Kinetics of the HRP-Catalyzed Oxidation of DCFH$_2$	Fig. S27<br>Fig. S28<br>Fig. S29	53
26	Effect of Light on the Quantification of H_2O_2 with the Cascade Reaction	Fig. S30<br>Fig. S31	58
27	Comparison of the HRP/H_2O_2-Catalyzed Oxidation of either DCFH$_2$-MA (Intermediate of the Cascade Reaction with BCA and DCFH$_2$-DA) or DCFH$_2$		62
28	Analysis of the Cascade Reaction Outflow from Two Types of Reactor Systems − Consisting of either Sequentially or Co-immobilized BCA and HRP − Through Which a Solution of DCFH$_2$-DA and H_2O_2 as Substrates was Pumped	Fig. S32<br>Fig. S33	64
29	Effect of Varying the Enzyme Reactor Length on the Outcome of the Cascade Reaction with DCFH$_2$-DA and H_2O_2 as Substrates Using Enzyme Reactor Systems with Sequentially Immobilized BCA and HRP	Fig. S34	66
30	Determination of the Steady-State Composition in the Outflows from Different Enzyme-Reactor Systems Through Which a Substrate Solution Consisting of DCFH$_2$-DA and H_2O_2 were Pumped	Table S6<br>Fig. S35	68
31	Stability of H_2O_2 in Bulk Solution in the Presence of BCA or in Enzymatic Flow-Through Reactors Containing either *de*-PG2$_{1000}$-BAH-BCA$_{89}$ or *de*-PG2$_{1000}$-BAH-HRP$_{20}$	Fig. S36<br>Fig. S37	70
32	Quantification of DCF in the Cascade Reaction Outflow from Two Types of Reactor Systems − Consisting of either Sequentially or Co-immobilized BCA and HRP − Through Which a Solution of DCFH$_2$-DA and H_2O_2 as Substrates was Pumped	Fig. S38<br>Fig. S39	73
33	References		75

1. HRP-Activity Assay in Bulk Solution with $ABTS^{2-}$ and H_2O_2 as Substrates

To a disposable polystyrene cuvette (l = 1 cm, 1.4 mL), PBS was added (100 mM phosphate, 150 mM NaCl, pH = 7.2). From an HRP-containing solution (in PBS), an amount was added so that the combined volume was 940 µL. After addition of $ABTS^{2-}$ (50 µL, 20 mM in PBS), the solution was mixed twice by withdrawing and releasing the entire volume with a 1 mL pipette tip. After addition of H_2O_2 (10 µL, 20 mM in H_2O), the reaction mixture was quickly mixed and spectra were recorded every 5 s for 2 min. The increase of A_{414} was linearly fitted against time for initial reaction rates (dA_{414}/dt, ε_{414} ($ABTS^{\bullet-}$) = 36 000 $M^{-1}cm^{-1}$).[S1]

For a calibration curve correlating known concentrations of native HRP (ε_{403} (HRP) = 102 000 $M^{-1}cm^{-1}$)[S2] to initial reaction rates (dA_{414}/dt), see **Figure S1a** ([HRP] = 0, 50 – 4000 pM).

When being required to quantify the activity of very low HRP concentrations, a second calibration curve was used, see **Figure S1b** below ([HRP] = 0, 2 – 200 pM, recording spectra every 10 s for 5 min). The observed rate constants (slope, $k_{obs} = v_{in}$ $[HRP]^{-1}$) determined from the two calibration curves were very similar. For very dilute analyte solutions, a detection limit of [HRP] = 5 pM applied.

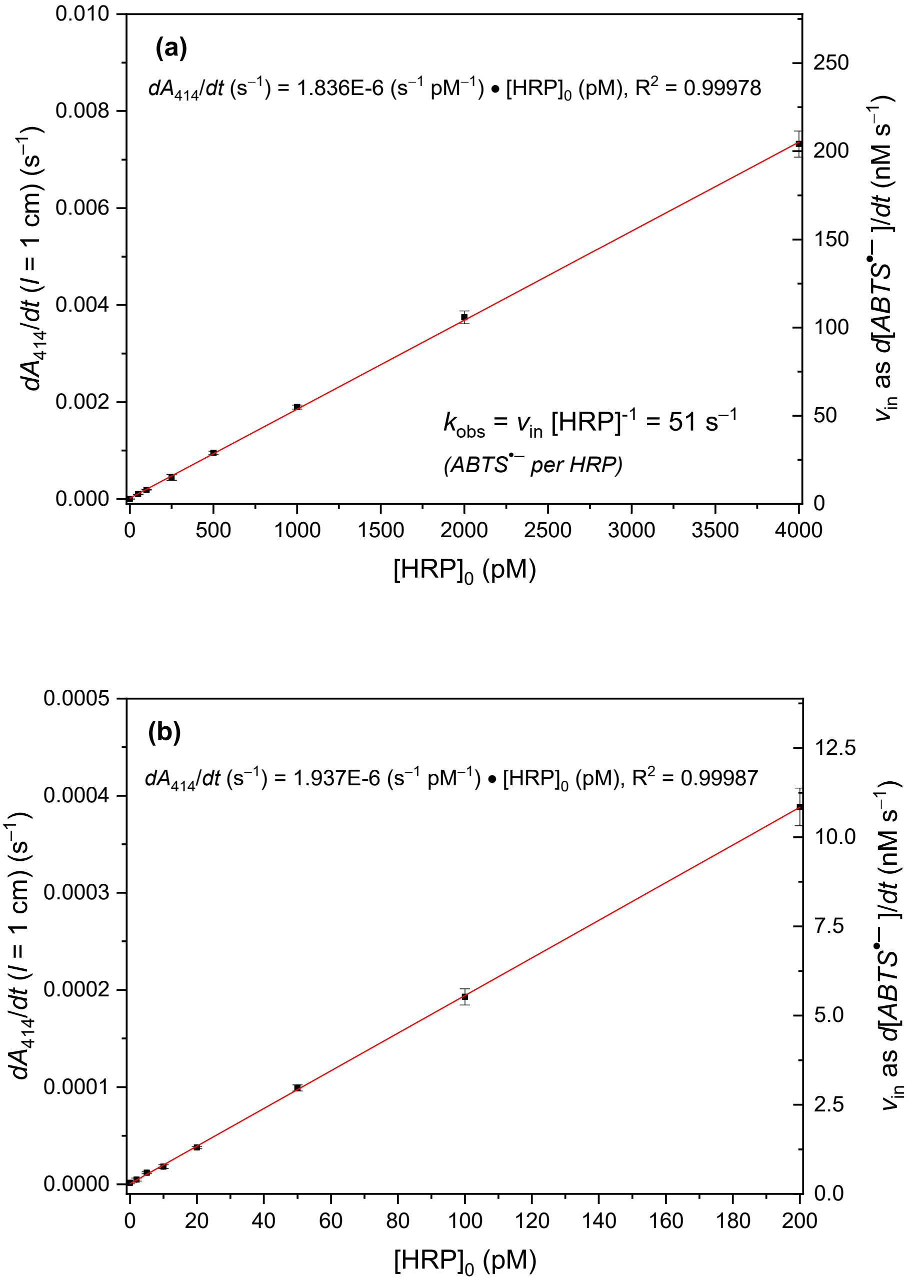

Figure S1. Calibration curves with native HRP. **(a)** 50-4000 pM or **(b)** 2-200 pM, in PBS (100 mM phosphate, 150 mM NaCl, pH = 7.2) using $ABTS^{2-}$ (1 mM) and H_2O_2 (0.2 mM). Spectra were recorded (a) every 5 s for 2 min or (b) every 10 s for 5 min and the obtained initial reaction rates (*dA_{414}/dt*) were fitted against the used HRP concentrations in the cuvette (red line). Error bars represent standard deviations from triplicates (fresh reaction mixtures). For calculating [$ABTS^{\bullet-}$], ε_{414} ($ABTS^{\bullet-}$) = 36 000 $M^{-1}cm^{-1}$ was used.[S1]

2. BCA-Activity Assay in Bulk Solution with PNPA as Substrate

To a disposable polystyrene cuvette (l = 1 cm, 1.4 mL), PB was added (10 mM phosphate, 0 mM NaCl, pH = 7.2). From a BCA-containing solution, an amount was added so that the combined volume was 990 µL. After addition of PNPA (10 µL, 100 mM in acetonitrile), the solution was mixed three times by withdrawing and releasing the whole volume with a 1 mL pipette tip. Spectra were recorded every 5 s for 3 min. The increase of A_{405} was linearly fitted against time.

From the resulting initial reaction rates (dA_{405}/dt, ε_{405} (*p*-nitrophenol/*p*-nitrophenolate) = 10 510 $M^{-1}cm^{-1}$ at pH = 7.2),[S3] the auto-hydrolysis rate for mixtures without added BCA had to be subtracted first (~ 2.4 nM/s; ≙ catalysis by ~ 3 nM BCA). The corrected reaction rates were compared to a calibration curve with known amounts of native BCA (ε_{280} (BCA) = 56 000 $M^{-1}cm^{-1}$),[S4] as obtained previously by Yoshimoto et al. (2018).[S5] The following equation resulted from the calibration curve: $\Delta A_{405} / \Delta t$ (s^{-1}) = 8.17×10^{-6} (s^{-1} nM^{-1}) · [BCA] (nM), corresponding to an observed rate constant ($k_{obs} = v_{in}$ $[BCA]^{-1}$) of 0.78 s^{-1}. For very dilute analyte solutions, a detection limit of [BCA] = 3 nM applied. In the presence of 3 nM BCA, the initial rate of PNPA hydrolysis was two times higher than the initial rate of PNPA hydrolysis in the presence of buffer only (non-enzymatic background hydrolysis).

3. Visualization of the Conjugation Reaction and of the Side Reactions Between the HyNic Linker Group and Native Enzymes

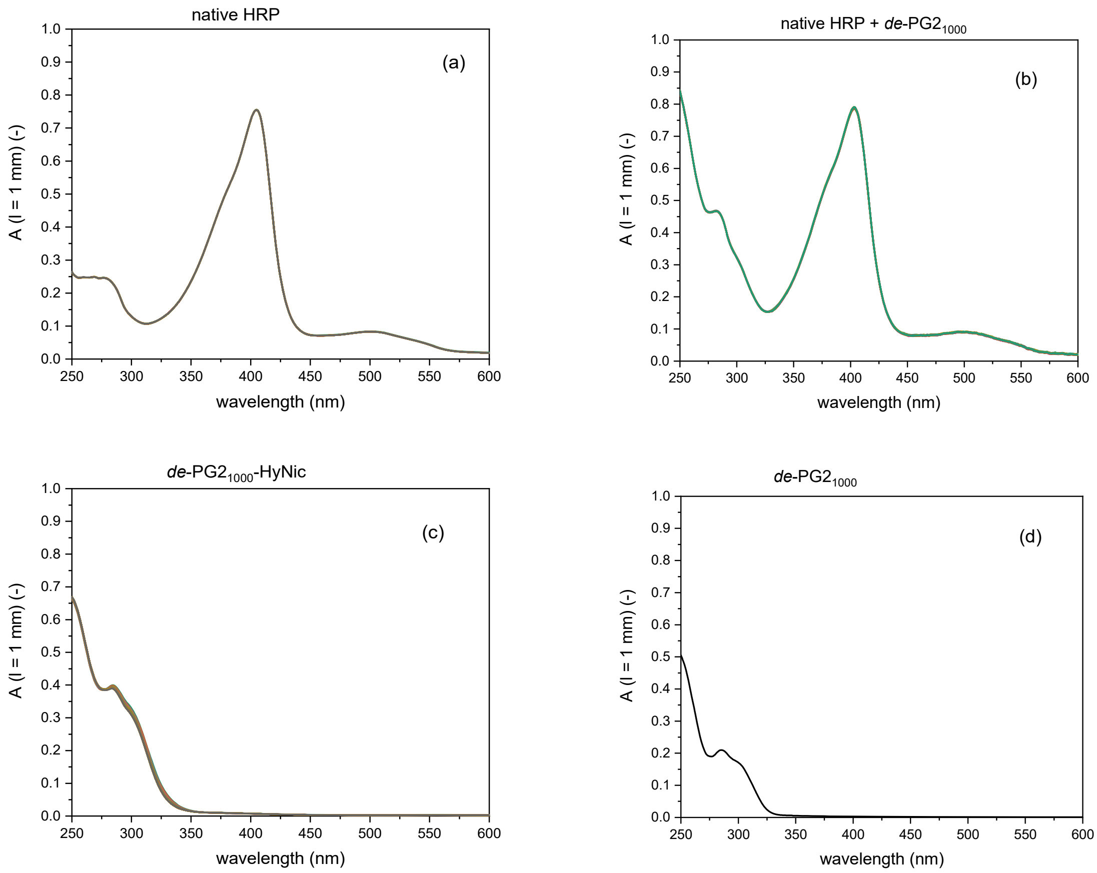

Figure S2. UV/vis absoprtion spectra of native HRP **(a)**, a mixture of native HRP and *de*-$PG2_{1000}$ **(b)**, *de*-$PG2_{1000}$-$HyNic_{240}$ **(c)**, and *de*-$PG2_{1000}$ at [r.u.] = 420 µM, (ε_{285} (r.u.) = 5000 $M^{-1}cm^{-1}$)[S6] **(d)**, dissolved in MES buffer (0.1 M MES, 0.15 M NaCl, pH = 4.7). Quartz cuvettes (l = 0.1 mm) were used. For (a) - (c), the solutions were incubated for 18 h at 25 °C wit recording of the UV/vis spectra every 15 min. [HRP] = 75 µM, denpol [r.u.] = 420 µM. All components were considered stable. The small change for *de*-$PG2_{1000}$-$HyNic_{240}$, (c), was negligible (possibly originating from adsorption of a fraction of the modified denpol on the cuvette wall) if compared to cases where HRP was present as well (see **Figure S3**). The modified denpol *de*-$PG2_{1000}$-$HyNic_{240}$ was already stored in the same buffer solution at higher concentration for > 24 h before the experiment.

During the conjugation reaction of HRP_1-$4FB_{0.73}$ ($[4FB]_0$ = 50 µM) with *de*-$PG2_{1000}$-$HyNic_{240}$ ($[HyNic]_0$ = 100 µM, 25 °C, pH = 4.7, 20 h), some spectral changes were observed that could not be assigned to BAH-bond formation, see **Figure S3a** (*e.g.*, decrease around λ = 403 nm, increase

around λ = 418 nm). Indeed, when performing the same reaction with the same amount of unmodified native HRP instead of HRP-4FB (using the same batch of *de*-PG2$_{1000}$-HyNic$_{240}$), all spectral changes from the conjugation reaction − except the dominant increase around λ = 354 nm from the BAH-bond − were found to be roughly the same; see **Figure S3b**. Therefore, these spectral changes must originate from a side reaction.

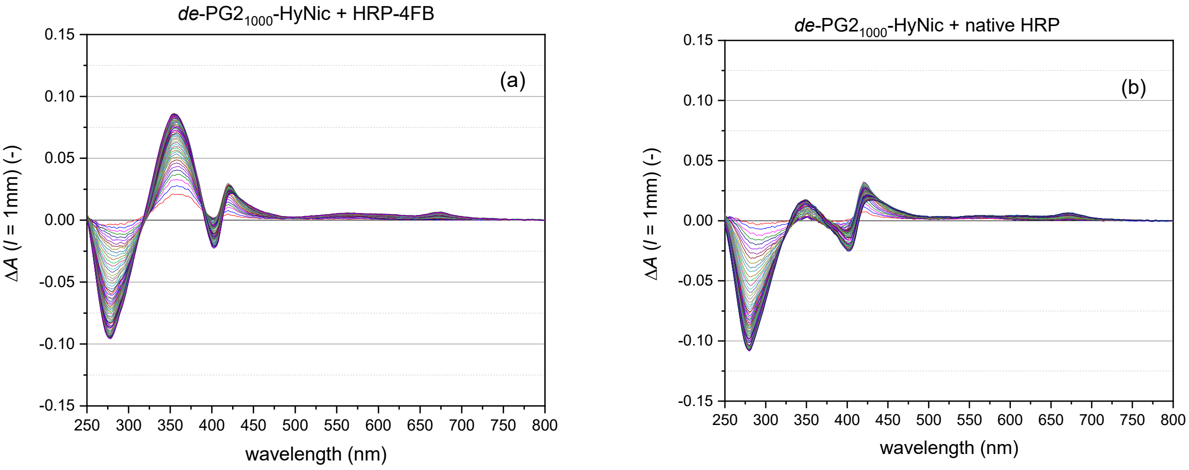

Figure S3. Differential UV/vis absorption spectra for mixtures of *de*-PG2$_{1000}$-HyNic$_{240}$ with **(a)** HRP-4FB to yield *de*-PG2$_{1000}$-BAH-HRP$_{17}$ or **(b)** native HRP. The components were dissolved in MES buffer (0.1 M MES, 0.15 M NaCl, pH = 4.7) and incubated in a 1 mm quartz cuvette for 18 h at 25 °C with recording of the UV/vis spectra every 15 min. [HRP] = 68 µM, denpol [r.u.] = 417 µM. The only significant difference between the reaction with 4FB-modified HRP and the reaction with unmodified HRP is around λ = 350 nm. Changes at λ ≈ 350 nm are due to BAH-bond formation (HyNic reacting with 4FB) (a). The comparatively small changes at λ ≈ 325-375 nm in the reaction with native HRP (b) originate either from changes of the enzyme's heme group peak or from new peaks originating from a side reactions of HyNic (*e.g.*, with a functional group of the enzyme).

The side reaction observed between *de*-PG2$_{1000}$-HyNic and HRP did not occur when either HRP or the HyNic group were missing and did thus not originate from an interaction between HRP and the unmodified dendronized polymer (see **Figure S2a – c**).

In a preliminary 4FB quantification reaction with 2-hydrazinopyridine (as used in our previous works), HRP-4FB was exposed to a large excess of 2-hydrazinopyridine at elevated temperature (500 µM 2-hydrazinopyridine, 6 µM HRP-4FB, 40 °C, see **Figure S4**.

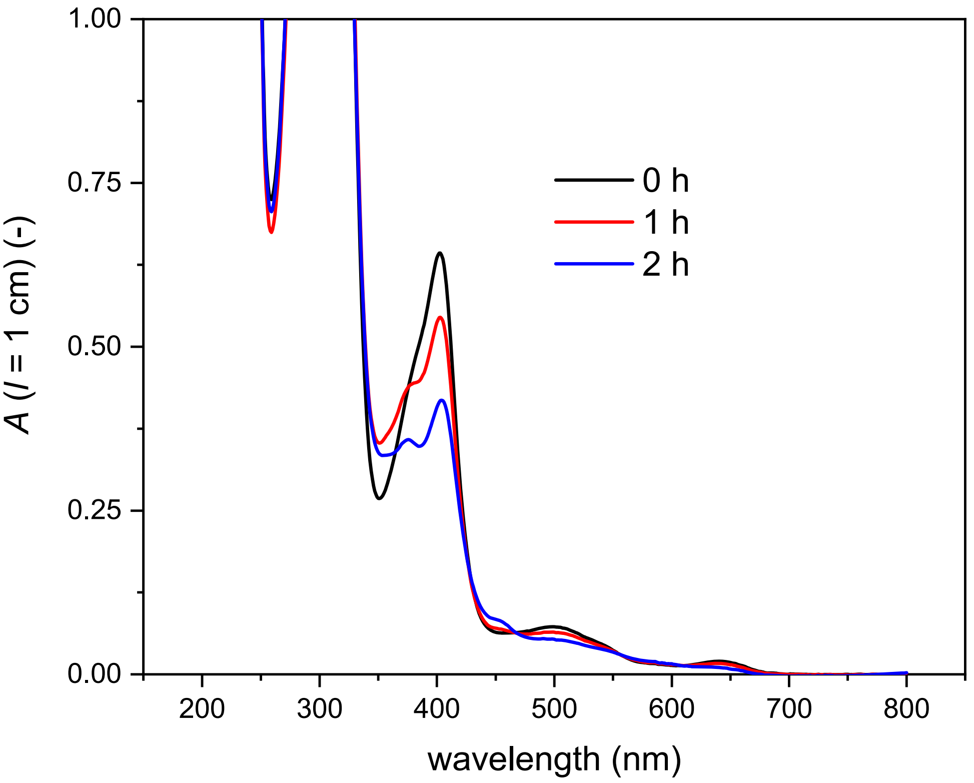

Figure S4. UV/vis absorption spectra of HRP-4FB (6 µM HRP) in reaction with 500 µM 2-hydazinopyridine dihydrochloride. All solutions were diluted with MES buffer (0.1 M MES, pH = 5.0), and the spectra were recorded against MES buffer at 40 °C. Heme peak changes (*e.g.*, decrease at λ = 403 nm and increase at λ = 420 nm) are visible together with concomitant BAH-bond formation (λ = 350 nm). The same side reaction (yet less pronounced) was also visible if the reaction was run at room temperature.

Besides the expected increase at $\lambda = 354$ nm used for 4FB quantification over the formed BAH bond, similar side reactions as in the conjugation reaction were observed (*e.g.*, decrease around $\lambda = 403$ nm, increase around $\lambda = 418$ nm, compare with **Figure S3**). At room temperature, these side reactions in the quantification reaction of 4FB were less pronounced, yet clearly visible. Since the decrease in the heme peak ($\lambda_{max} = 403$ nm) did also lower A_{354}, the determined [4FB] in solutions of HRP-4FB was probably a bit too low when using this quantification reaction. This was an additional reason to change to the spectral fitting for the determination of [4FB] in HRP-4FB (besides the smaller effort of the fitting). The observed side reaction could be due to covalent reactions between the hydrazine and the apoenzyme near or at the active site of HRP, as reported for several hydrazine species.[S7] Thereby, a concomitant loss of enzymatic activity and the heme peak ($\lambda_{max} = 403$ nm) was reported (see *"Fig. 1-3"* in Hidaka et al. (1970).[S7] During our conjugation reaction the loss of the heme peak during the conjugation reaction was only ≈ 3 % and did presumably not disturb the quantification of the BAH bond at $\lambda = 354$ nm significantly (see **Figure S3**). No activity measurements during conjugation reaction were carried out in this work but in a previous investigation,[S8] the enzymatic activity was found to be stable during a similar conjugation reaction of HRP-4FB and HyNic groups attached to polylysine.

When looking at the conjugation reaction of *de*-$PG2_{1000}$-$HyNic_{362}$ with BCA_1-$4FB_{0.84}$ (and the control experiment with native BCA) in **Figure S5**, the only *visible* side reaction was probably the auto-oxidation of the pyridine bound hydrazine group at pH > 7.[S9] A covalent attachment of HyNic to some residues of native BCA could not be excluded (since no easily visible spectral indicator like the heme group in HRP is present in BCA).

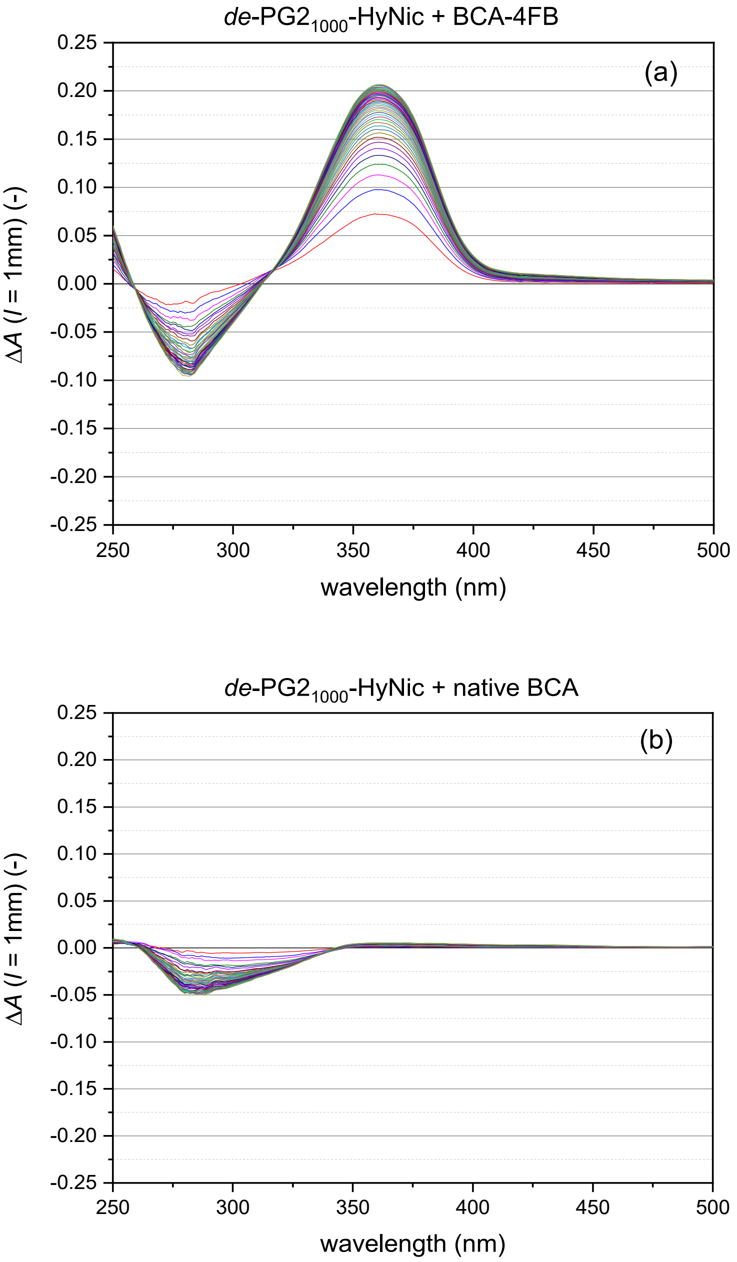

Figure S5. Differential UV/vis absorption spectra for mixtures of *de*-PG2$_{1000}$-HyNic$_{362}$ and BCA-4FB to yield *de*-PG2$_{1000}$-BAH-BCA$_{89}$ **(a)** or native BCA **(b)**. The components were dissolved in PBS* (0.1 M phosphate, 1.15 M NaCl, pH = 7.2) and incubated in a 2 mm quartz cuvette for 20 h at 25 °C, taking UV/vis spectra every 30 min (difference calculated and displayed for 1 mm path length to compare with **Figure S3**). [BCA] = 71 µM, denpol [r.u.] = 331 µM. For the reaction with native BCA **(b)**, the decrease at $\lambda \approx 280$ nm was about half the decrease observed for the reaction with BCA-4FB (a); at $\lambda \approx 350$ nm, there was no increase (no BAH-bond formation). The formation of this bond in **(a)** shows an isosbestic point at $\lambda = 317$ nm, establishing after 1 h of reaction time.

4. Details about the Conjugate Preparation

Except for some changes in the listed parameters below, the conjugates were prepared as reported before: Hou et al. (2019)[S10] for HRP (see *de*-$PG2_{1000}$-BAH-HRP_{17} in Chapter 4.1. below) and Yoshimoto et al. (2018)[S5] for BCA. For spectral changes during conjugate formation (BAH bond formation around λ = 354 nm), see **Figure S2a** (HRP) and **Figure S5a** (BCA) in Chapter 3.

4.1. Characteristic Data for *de*-$PG2_{1000}$-BAH-HRP Conjugate Preparation

de-$PG2_{1000}$-BAH-HRP_{20}:

- MSR (4FB/HRP) = 0.89 (spectral fitting only)
- *de*-$PG2_{1000}$-$HyNic_{238}$ (spectral fitting for [HyNic], [r.u.] with the trypan blue assay)
- conjugation reaction conditions: $[4FB]_0$ = 61 µM, $[HyNic]_0$ = 100 µM, pH = 4.7
- components added: V = 1 mL, $[HRP]_{added}$ = 69 µM, $[r.u.]_{added}$ = 420 µM
- observed in conjugation reaction: $[BAH]_{rxn}$ = 33.4 µM
- Purified by repetitive ultrafiltration using PBS* (100 mM NaH_2PO_4, 1.15 M NaCl, pH = 7.2) and a 4 mL, 100 kDa MWCO Amicon centrifugal filter (centrifuged at 2 500 g). Filtrates monitored at l = 1 cm (for A_{403}). Retentate collected in same volume as added for purification (1 mL).

de-$PG2_{1000}$-BAH-HRP_{40}:

- MSR (4FB/HRP) = 0.91 (spectral fitting only)
- *de*-$PG2_{1000}$-$HyNic_{227}$
- conjugation reaction conditions: $[4FB]_0$ = 65 µM, $[HyNic]_0$ = 100 µM, pH = 4.7
- components added: V = 0.35 mL, $[HRP]_{added}$ = 71 µM, $[r.u.]_{added}$ = 441 µM
- observed in conjugation reaction: $[BAH]_{rxn}$ = 31.6 µM
- Purified by repetitive ultrafiltration using PBS* (100 mM NaH_2PO_4, 1.15 M NaCl, pH = 7.2) and a 4 mL, 100 kDa MWCO Amicon centrifugal filter (centrifuged at 2 500 g and $T \approx$ 4 °C). Filtrates monitored at l = 1 cm (for A_{403}). Retentate collected in same volume as added for purification (0.35 mL).

de-$PG2_{1000}$-BAH-HRP_{17} (described in Hou et al. (2019)[S10] as *de*-$PG2_{1000}$-BAH-HRP_{71}):

The conjugate *de*-$PG2_{1000}$-BAH-HRP_{17} was used in our earlier work, where it was named *de*-$PG2_{1000}$-BAH-HRP_{71} (due to another conjugate quantification, see Section 2.4. and/or Section 3.1.3.). Some unpublished data measured previously with this HRP-conjugate were re-analyzed (according insights gained within this work) and the results compared to the new batches. For the preparation protocol of the HRP-conjugate, we followed mainly the protocol for this conjugate

(see Hou et al. (2019),[S10] Supporting Information, Chapter 11). The protocol for other conjugates within this earlier work differed.

- MSR (4FB/HRP) = 0.73
- *de*-PG2$_{1000}$-HyNic$_{240}$
- conjugation reaction conditions: [4FB]$_0$ = 50 µM, [HyNic]$_0$ = 100 µM, pH = 4.7
- components added: V = 0.35 mL, [HRP]$_{added}$ = 68 µM, [r.u.]$_{added}$ = 417 µM
- observed in conjugation reaction: [BAH]$_{rxn}$ = 29.5 µM
- Purified by repetitive ultrafiltration using phosphate buffer (10 mM NaH_2PO_4, 150 mM NaCl, pH = 5.0) and a 4 mL, 100 kDa MWCO Amicon centrifugal filter (centrifuged at 2 500 g). Filtrates monitored at l = 1 mm (for A_{403}). Retentate collected in same volume as added for purification (0.35 mL).

4.2. Characteristic Data for *de*-PG2$_{1000}$-BAH-BCA Conjugate Preparation

de-PG2$_{1000}$-BAH-BCA$_{54}$:

- MSR (4FB/BCA) = 1.0
- *de*-PG2$_{1000}$-HyNic$_{330}$ (spectral fitting for [HyNic], [r.u.] with the trypan blue assay)
- conjugation reaction conditions: [4FB]$_0$ = 60 µM, [HyNic]$_0$ = 120 µM, pH = 7.2
- components added: V = 1 mL, [BCA]$_{added}$ = 60 µM, [r.u.]$_{added}$ = 364 µM
- observed in conjugation reaction: [BAH]$_{rxn}$ = 57 µM
- Purified by repetitive ultrafiltration using PBS* (100 mM NaH_2PO_4, 1.15 M NaCl, pH 7.2) and two 0.5 mL, 50 kDa MWCO Amicon centrifugal filters (centrifuged at 16 100 g instead of 2 500 g). Filtrates monitored at l = 1 cm (for A_{280}). Retentate collected in same volume as added for purification (1 mL).

de-PG2$_{1000}$-BAH-BCA$_{89}$ (described in Ghéczy et al. (2020)[S11] as *de*-PG2$_{1000}$-BAH$_{207}$-BCA$_{152}$):

Note that the conjugate de-PG2$_{1000}$-BAH-BCA$_{89}$ was prepared for experiments in this current and our already published work. There, it was named *de*-PG2$_{1000}$-BAH$_{207}$-BCA$_{152}$ (due to another conjugate quantification, see Section 2.4 and/or Section 3.1.3.).

- MSR (4FB/BCA) = 0.84
- *de*-PG2$_{1000}$-HyNic$_{362}$
- conjugation reaction conditions: [4FB]$_0$ = 60 µM, [HyNic]$_0$ = 120 µM, pH = 7.2
- components added: V = 2.5 mL, [BCA]$_{added}$ = 71 µM, [r.u.]$_{added}$ = 331 µM
- observed in conjugation reaction: [BAH]$_{rxn}$ = 68 µM
- Purified by repetitive ultrafiltration using PBS* (100 mM NaH_2PO_4, 1.15 M NaCl, pH = 7.2) and a 4 mL, 50 kDa MWCO Amicon centrifugal filter (centrifuged at 2 500 g). Filtrates monitored at l = 0.2 cm (for A_{280}). Retentate collected in same volume as added for purification (2.5 mL).

5. Characterization of Purified Conjugate Stock Solutions

Table S1. Concentrations obtained in purified conjugate stock solutions and corresponding yields regarding the conjugation reaction before purification (see Chapter 4 for the conditions of the conjugation reactions and conjugate purifications and Section 2.5. for conjugate characterization).

Purified Conjugate[a] *de*-PG2$_{1000}$-BAH-enzyme$_y$	BCA$_{54}$	BCA$_{89}$	HRP$_{20}$	HRP$_{40}$	HRP$_{17}$[b]
Volume	1 mL	2.5 mL	1 mL	0.35 mL	0.35 mL
[r.u.] (µM)	138.0	290.0	109.8[c]	43.6	283.5
[BAH] (µM)	21.5	60.0	8.7	3.1	20.2
[Enzyme][d] (µM)	7.4 ± 2.0	25.7 ± 2.3	2.2 ± 1.0	1.8 ± 0.0	5.0 ± 4.0
Apparent Conjugation Yield (BAH_{rm} / $Enzyme_{rm}$)[e]	94.8 %	95.2 %	48.8 %	44.3 %	43.1 %
Denpol Recovery Yield (BAH_{pur} / BAH_{rm})[e]	38.0 %	87.5 %	26.1 %	9.9 %	68.5 %
Activity per Linker Yield ($Enzyme_{pur}$ / BAH_{pur})[e]	34.4 %	42.8 %	25.3 %	56.3 %	24.5 %
Overall Activity Yield ($Enzyme_{pur}$ / $Enzyme_{rm}$)[e]	12.4 %	35.6 %	3.2 %	2.5 %	7.2 %

[a] Subscripts for enzymes from the ratio of [Enzyme]/[r.u.].
[b] Conjugate prepared in previous work, listed for comparison, see Chapter 4.1.
[c] Same value (109.5 µM) obtained with the trypan blue assay (see Hou et al. (2019)[S10] for the trypan blue assay).
[d] Determined by activity assay in bulk solution, using $ABTS^{2-}/H_2O_2$ for HRP and PNPA for BCA as substrates (see Experimental Section). Standard deviation from n = 10, 6, 18, 3, 15 measurements within the first 12, 5, 3, 1, 1 months after conjugate preparation.
[e] The subscripts "rm" and "pur" refer to the reaction mixture (after completion of the reaction) and the purified conjugate stock solution, respectively. [BAH] is the molar concentration of bis-aryl hydrazone bonds as calculated using ε_{354} (BAH) = 29 000 M^{-1} cm^{-1}, see Küchler et al., (2017).[S12] The molar amount of enzyme was determined by activity assay in bulk solution, using $ABTS^{2-}/H_2O_2$ for HRP and PNPA for BCA as substrates (see Experimental Section). While the overall activity yield for the preparation of a conjugate stock solution could be simply determined by the amount of active enzyme in the stock solution compared to the amount added to the conjugation reaction, it is also represented as product of the yields of the three steps of the entire conjugate preparation: "Overall Activity Yield" = "Apparent Conjugation Yield" x "Denpol Recovery Yield" x "Activity per Linker Yield".

Table S2. The different conjugate batches were compared regarding the purification conditions, the denpol recovery yield upon purification and the enzyme leakage upon later adsorption of the purified conjugates in porous monoliths.

Purified Conjugate *de*-$PG2_{1000}$-BAH-$enzyme_y$	BCA_{54}	BCA_{89}	HRP_{40}	HRP_{20}	HRP_{17}[d]
Dilution Factor$^{\text{Dilution Cycles}}$ in Purification[a] (Repetitive dilution and centrifugation)	2^{22}	2^{20}	2.5^{19}	4^{8}	4^{6}
Denpol Recovery Yield[b] (before *vs.* after purification)	38.0 %	87.5 %	9.9 %	26.1 %	68.5 %
Eluting after Conjugate Adsoption[c] (1 – enzyme immobilization yield)	0 %	2.4 %	0 %	0.3 %	4.2 %

[a] The purification was separating non-conjugated enzymes from the conjugates by repetitive ultrafiltration. The reaction mixture (after completion of the reaction) was diluted with buffer solution and concentrated by centrifugation through a size-exclusive filter membrane repeatedly. See Chapter 4 for further purification conditions.
[b] The denpol recovery yield was determined by comparing the amount of r.u. before and after the purification of the conjugate, see **Table S1**.
[c] Note that all monolith pieces were exposed to conjugate incubation solutions at conjugate concentrations that did not result in internal monolith surface saturation (as defensively estimated). This means that all conjugates "would find" enough area if enough time was given and no desorption occurred (see Results and Discussion, Section 3.2.1.). The percentage value is for the ratio of active enzymes that were found upon buffer washing during reactor preparation (compared to the incubated amount of enzme). For the washing experiments, see later Chapter 13.
[d] Conjugate from a previous work for comparison, see Chapter 4.1.

6. Effect of Storage and Aliquot Sampling on Enzyme Activity of Diluted Conjugate Stock Solutions

Only 50 µL aliquots of the purified conjugate stock solutions were required for incubation in monolith pieces of length l_m = 5 mm for the preparation of enzymatic flow-through reactors (see Section 2.6. and 3.2.2.). The conjugate incubation solutions that were added to the monolith pieces were obtained by dilution of the conjugate stock solutions shortly before application. We already knew that stock solutions of *de*-$PG2_{1000}$-BAH-HRP_{20} and *de*-$PG2_{1000}$-BAH-BCA_{54} are stable if stored as obtained after purification. For testing the storage stability of different diluted stock solutions, the concentrated stock solutions (as obtained after purification) were diluted 10- or 100-fold and stored in polypropylene tubes for several days at 4 or 25 °C with intermediate aliquot sampling for activity measurements. The same was done with stock solutions of respective native enzymes. See **Figure S6**. In all four cases, there was some activity loss upon storage after dilution, while the activity remained relatively stable if stored undiluted (with some experimental variation). Note that the activity decrease was not continuous but rather the values for the enzyme activities were stabilizing over time. The reason for this equilibration on a lower level is not known. One possibility is that some conjugate/enzyme accumulated at the wall of the polypropylene tubes the conjugate solutions were stored in. For the activity assay, aliquots were withdrawn and the assay was performed in polystyrene cuvettes. Thereby, potentially wall-accumulated enzymes did not take part in the assay. While the BCA-conjugate showed a similar behavior as the native enzyme, the HRP-conjugate seemed particularly susceptible to the observed "dilution-effect", independent on the storage temperature. In both cases, preparing (by dilution) a conjugate incubation solution for immobilization already days in advance of the solid support exposure was expected to lead to losses in conjugate activity. Consequently, all conjugate incubation solutions used for

immobilizations were diluted just shortly before solid support exposure and were transferred in the support as quickly as possible. The conjugate incubation solution thereafter spent some time in the solid support to allow for conjugate adsorption on the silica surface (3 h); see **Figure 2**.

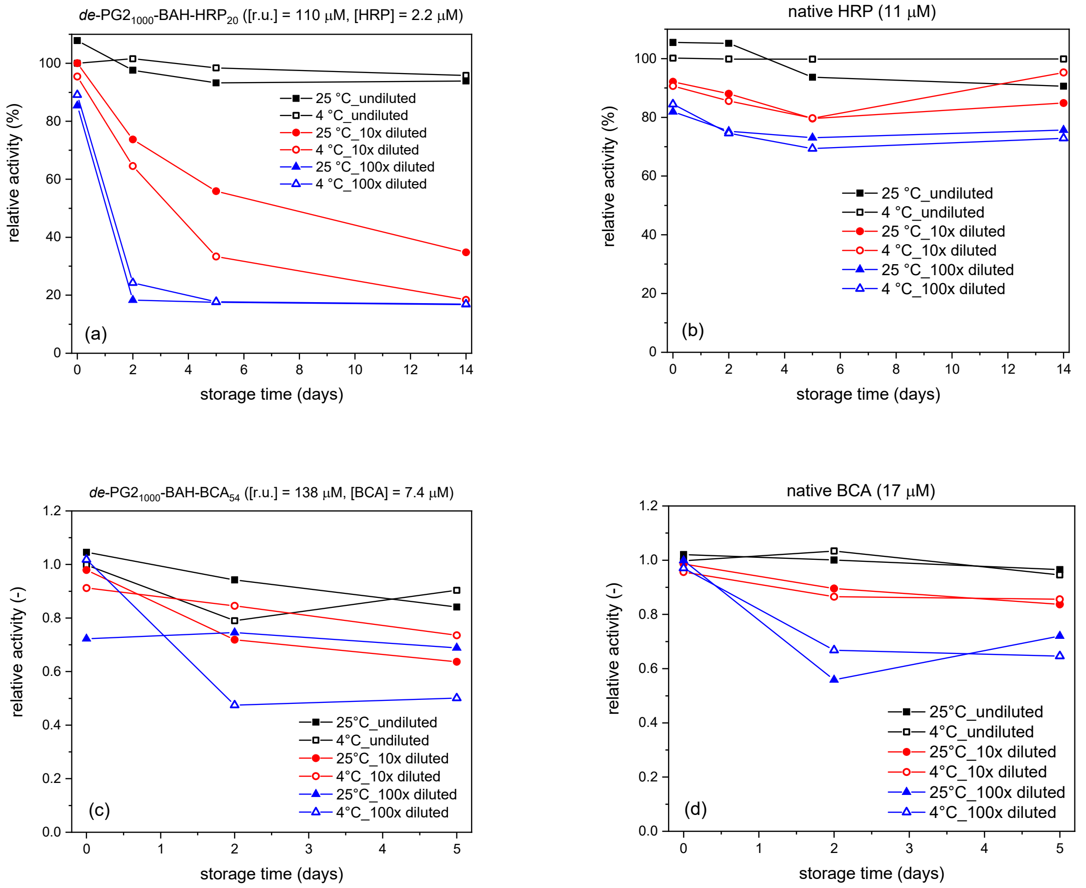

Figure S6. Relative activity of aqueous solutions of PBS* (100 mM phosphate, 1.15 M NaCl, pH = 7.2) of **(a)** *de*-PG2$_{1000}$-BAH-HRP$_{20}$, **(b)** native HRP, **(c)** *de*-PG2$_{1000}$-BAH-BCA$_{54}$, and **(d)** native BCA. The black data points represent the respective stock solutions, whereas the red data points refer to a 10-fold and the blue data points to a 100-fold dilution (in PBS). The resulting samples were measured directly after dilution (day 0) and after the indicated days of storage in polypropylene tubes at 25 °C (filled symbols) or 4 °C (empty symbols). The activity is given relative to the respective activity of the stock solution at day 0 that was then stored at 4 °C (black empty symbol). The HRP activity was measured with the $ABTS^{2-}/H_2O_2$ assay, the BCA-activity with the PNPA assay (see Section 2.3.1. and Section 2.3.2., respectively). For the activity measurements, aliquots of the respective solutions were diluted accordingly to always yield the same enzyme concentrations in the cuvette ([HRP] ≈ 1 nM, BCA ≈ 100 nM). Lines between data points are for guiding the eye only.

7. Comparison of the Activity of *de*-PG2$_{1000}$-BAH-HRP$_{20}$ to the Activity of Native HRP Using Different Substrates

The HRP concentration in stock solutions of *de*-PG2$_{1000}$-BAH-HRP$_{20}$ was determined by using the ABTS-assay and solutions of known concentrations of native HRP ("activity-based HRP concentration"): $[ABTS^{2-}]_0$ = 1.0 mM, $[H_2O_2]_0$ = 0.2 mM, PBS (100 mM phosphate, 150 mM NaCl, pH = 7.2), RT. Usually, the conjugate stock solution was diluted to [HRP] ≈ 1 nM inside the cuvette shortly before starting the reaction, detecting product formation for 2 min (see Section 2.3.1.).

With $DCFH_2$ as substrate, the conditions were $[DCFH_2]_0$ = 50 μM, $[H_2O_2]_0$ = 30 μM, PBS (100 mM phosphate, 150 mM NaCl, pH = 7.2), RT. The conjugate stock solution was diluted to [HRP] ≈ 100 nM (based on the ABTS-assay) inside the cuvette shortly before starting the reaction, detecting product formation for 60 min (see later Chapter 25 for the assay of the HRP-catalyzed oxidation of $DCFH_2$ with H_2O_2 as terminal oxidant).

The third substrate used was $DCFH_2$-MA, which was *in situ* formed from $DCFH_2$-DA with BCA: $[DCFH_2\text{-DA}]_0$ = 50 μM, $[H_2O_2]_0$ = 30 or 10 μM and [BCA] = 1.5 μM, PBS (100 mM phosphate, 150 mM NaCl, pH = 7.2), RT. The conjugate stock solution was diluted to [HRP] ≈ 100 nM (based on the ABTS-assay) inside the cuvette shortly before starting the reaction, detecting the progress of the reaction for up to 60 min (see Section 2.3.3. and later Chapter 24 for the cascade assay). Under the chosen conditions, HRP predominantly catalyzed the oxidation of the intermediate $DCFH_2$-MA (see Results and Discussion, 3.4.1.).

The performance of the conjugate was correlated to the respective observed rate constants (k_{obs} = v_{in} $[HRP]^{-1}$) obtained for native HRP in bulk solution: 51 s^{-1} in the ABTS-assay (**Figure S1a**); 0.3 s^{-1} in the $DCFH_2$-assay (later **Figure S27**); 4.5 or 3.5 × 10^{-2} s^{-1} for $DCFH_2$-MA using 30 or

10 µM H_2O_2, respectively (later **Figure S26**). Thereby, the HRP concentrations determined in one and the same conjugate stock solution with the three substrates were compared to each other and found to be very similar: 2.5 µM (ABTS-assay), 2.7 µM ($DCFH_2$-assay), and 2.5 or 2.6 µM (for $DCFH_2$-MA within the cascade reaction using 30 or 10 µM H_2O_2, respectively). Based on the similarity of these values (*i.e.*, their independency on the substrate used), it is reasonable to conclude that the activity-based determination of the HRP concentration in denpol-HRP conjugate stock solutions using the ABTS-assay yields reasonable values that are not biased by the substrate used; thus indicating the concentration of *fully active* HRP that was conjugated.

While quite similar K_M values were determined for conjugated and native enzymes in our previous works with HRP[S13] and BCA,[S5] the catalytic efficiency (k_{cat}/K_M) was estimated to decrease upon conjugation in these previous works. Since values of rate constants that are enzyme-specific, $k_{obs} = v_{in}$/[Enzyme] or $k_{cat} = v_{max}$/[Enzyme], require knowledge of the concentration of active enzyme molecules, determination of their correct concentration is essential. In previous determinations of the enzyme concentration in solutions of denpol-BAH-enzyme conjugates, the BAH bond concentration was determined − either in solution after the conjugation reaction was completed, or in solutions of purified conjugates − and this concentration was then taken as enzyme concentration (assuming that each enzyme molecule is linked to the denpol *via* a single BAH bond). This may have resulted in an incorrect value of the enzyme concentration (see later Chapter 10). In this present work, the catalytic efficiency (k_{cat}/K_M) of the enzymes that remained active was expected to not have decreased upon conjugation but instead their concentration was found to be lower than the one of the BAH bonds in the purified conjugate solutions (see **Table S1**).

8. Control Experiments with Mixtures of *de*-PG2$_{1000}$-HyNic$_{240}$ and Native HRP for Gaining Insights into Purified *de*-PG2$_{1000}$-BAH-HRP$_y$

In relation to the conjugate *de*-PG2$_{1000}$-BAH-HRP$_{17}$ a control experiment was carried out by using the same amounts of *de*-PG2$_{1000}$-HyNic$_{240}$ and *native HRP* (instead of HRP-4FB), see **Figure S3b**. After incubation for 18 h at RT, the mixture was purified the same way as in the case of the reaction mixture containing HRP-4FB (the same repetitive ultrafiltration protocol using the same volumes, see Chapter 4.1.). See **Figure S7** for the UV/vis absorption spectra of the two purified products and the difference spectrum (spectrum of the purified product obtained with HRP-4FB minus spectrum of the "product" obtained with native HRP). In the control experiment, it was expected to recover only the HyNic-modified dendronized polymer, without any bound HRP (purple spectrum). Indeed, neither a significant HRP activity nor a specific peak at $\lambda = 403$ nm was present in the spectrum. The solution of the processed *de*-PG2$_{1000}$-HyNic$_{240}$ was, however, slightly yellow (while the fresh stock solution was colorless without any absorbance above $\lambda = 300$ nm; see Figure S-4 in Hou et al. (2019)[S10]). The absorbance observed between $\lambda = 350$ and 700 nm could originate from modified HyNic groups since similar spectra (and a yellow color) were also observed for too long stored *de*-PG2$_{1000}$-HyNic stock solutions. Additionally, side reactions between native HRP and *de*-PG2$_{1000}$-HyNic$_{240}$ were clearly detected before (**Figure S3b**). Although the solution appeared transparent, some contribution from scattering could not be excluded. In the purified denpol-BAH-HRP conjugate, the apparent, activity-based HRP concentration was found to be lower than A_{403} would suggest ([HRP] $\approx 0.7 \times$ [HRP]$_{\text{based on } A403}$). When considering the difference spectrum (dashed blue spectrum in **Figure S7**), the obtained A_{403} value correlated very well with the measured HRP activity. This was the case for all HRP-conjugate batches analyzed in this work, using the very same "control experiment spectrum"

obtained with processed *de*-PG2$_{1000}$-HyNic$_{240}$ (similar *de*-PG2$_{1000}$-HyNic$_{x}$ solutions used in all HRP-conjugate batches, see Chapter 4.1.).

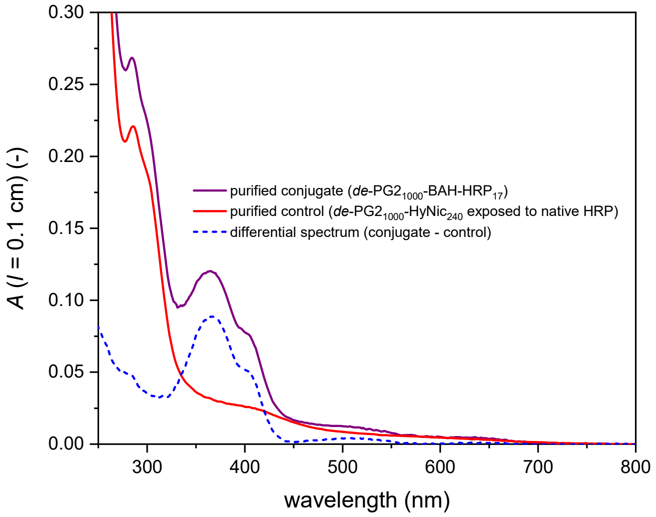

Figure S7. UV/vis absorption spectra of purified conjugate (*de*-PG2$_{1000}$-BAH-HRP$_{17}$, purple), of a purified reaction mixture originally consisting of *de*-PG2$_{1000}$-HyNic$_{240}$ and native HRP (red), and of the difference spectrum (blue). The two experiments were carried out by using identical *de*-PG2$_{1000}$-HyNic and HRP amounts and then purified in the same way (and with the same volumes). The resulting absoprtion at λ = 403 nm of the difference spectrum (A_{403} = 0.051) reflected a HRP concentration as expected from the ABTS assay with the purified conjugate solution, [HRP] = 5 µM, see **Table S1**). No HRP activity was found in the purified reaction mixture initially containing *de*-PG2$_{1000}$-HyNic$_{240}$ and native HRP (red spectrum), indicating (i) that native HRP did not bind to the HyNic-modifed denpol and (ii) that all native HRP was removed during purification. Note that the A_{403}/A_{354} ratio for the purified conjugate spectrum and the difference spectrum were about the same (0.67 and 0.63, respectively). This is relevant for the analysis described in Chapter 9.

As mentioned, neither a significant HRP activity nor a peak at λ = 403 nm was found in the retentate of the control experiment (recovering upon purification the HyNic-modified dendronized polymer without enzymes). In the filtrates, however, only ≈ 60 % of the initially added HRP amount was found (by assessing A_{403} in the filtrates at l = 1 cm). The amount of unbound HRP-4FB quantified during purification was used in our previous work for quantifying the amount of conjugated HRP in the conjugate (by mass balance).[S11] Thereby, potentially missed HRP-4FB might have led to a too high estimation of conjugated HRP.

9. Determination of the Denpol Repeating Unit Concentration, [r.u.], in Solutions of Purified *de*-PG2$_{1000}$-BAH-HRP$_y$

Firstly, the [HRP]/[BAH] ratio was calculated from the measured ratio of A_{403}/A_{354} in aqueous solutions containing purified denpol-BAH-HRP conjugates, see **Equation S1**. The molar absorptions used were ε_{403} (HRP) = 102 000 $M^{-1}cm^{-1}$, see Dunford and Stillman (1976),[S2] ε_{354} (HRP) = 35 000 $M^{-1}cm^{-1}$ (as determined experimentally), and ε_{354} (BAH) = 29 000 $M^{-1}cm^{-1}$, ε_{403} (BAH) ≈ 0 $M^{-1}cm^{-1}$, see Küchler et al. (2017).[S12] Then, with the determined ratio of [HRP]/[BAH], [BAH] was calculated by taking into account the measured HRP concentration in the conjugate solution (activity-based HRP concentration using the ABTS assay). Comparing the obtained [BAH] with the concentration of BAH bonds formed during the conjugation reaction (see **Figure S3a**), the denpol recovery yield was obtained. Note that this latter determination is independent of the correct value for ε_{354} (BAH), since ε_{354} (BAH) is expected to be the same before and after purification. A potentially too high/low value calculated for [BAH] in the purified solution, would be equally falsified for [BAH] in the reaction mixture before purification, and any potential deviation in ε_{354} (BAH) from the real value would cancel out for determining the denpol recovery yield.

For a representation of the same calculations for obtaining [r.u.] in the purified conjugate solution, not using ε_{354} (BAH) at all, see **Equation S2**.

$$[HRP]/[BAH] = \frac{\varepsilon_{354}(BAH)\cdot\frac{A_{403}}{A_{354}}}{\varepsilon_{403}(HRP) - \varepsilon_{354}(HRP)\cdot\frac{A_{403}}{A_{354}}}$$ **Equation S1**

$$[r.u.]_{purified} = \frac{A_{354}(from\ BAH_{purified}) * [r.u.]_{conjugation\ reaction}}{\Delta A_{354}(BAH_{conjugation\ reaction})}$$ **Equation S2**

$$with\ A_{354}(from\ BAH_{purified}) = (\varepsilon_{403}(HRP)\frac{A_{354}}{A_{403}} - \varepsilon_{354}(HRP)) * [HRP]_{activity\ assay}$$

10. Comments about the Conjugate Characterization

In our previous investigations, the number of conjugated enzyme molecules per denpol chain was quantified in different ways than in the present work. The previous quantification was either *via* the amount of BAH-bonds[S10] (calculated by taking into account ε_{354} (BAH) = 29 000 $M^{-1}cm^{-1}$, see Küchler et al. (2017)[S12] and assuming a 1:1 molar ratio of BAH to enzyme, or *via* mass balance considerations during conjugate purification.[S5, S11] In these previous works, the amount of covalently attached enzymes during the conjugation reaction (*before* purification) was estimated, aiming to provide an idea about the molecular structure of the conjugates and the spatial feasibility, see Fig.1 in Ghéczy et al. (2020).[S11] While the spectral quantification of unbound enzyme molecules that were separated during conjugate purification by repetitive ultrafiltration was found to be prone to an underestimation (see Chapter 8), more BAH-bonds than denpol-bound *active* enzymes (as determined by activity assays) were calculated within this work *after* purification ([HRP]:[BAH] = 0.25 − 0.55, see **Table S1**). There are different possibilities to explain imprecise values of number of bound enzyme molecules per denpol chain in our previous investigations. Excluding the presence of only "partially" active enzymes, the remaining reasons are either i) the presence of completely inactivated enzymes, ii) the presence of several BAH-bonds per denpol-bound enzyme; or iii) an overestimation of the actual BAH-bond concentration (determined *via* the molar absorption ε_{354}(BAH) = 29 000 $M^{-1}cm^{-1}$).[S12] We considered the presence of inactivated enzymes unlikely, due to the fact that A_{403} correlated well with the activity-based HRP concentration, [HRP], if the "spectral background" originating from other functional groups present in the conjugate and possibly light scattering was taken into account, see **Figure S7**.

11. Considerations about the Yields upon Conjugate Formation and Purification

While the conjugate preparations according to the developed protocols were found to be very reproducible (see Chapter 4), the determined number of active enzymes molecules per denpol chain and the denpol recovery yield upon conjugate purification showed higher variation (see **Table S1**). The reason for this can be seen in the conditions used for the conjugate purification. **Table S2** shows that not surprisingly a more extensive purification (*i.e.*, higher dilution or more centrifugation cycles) led to less remaining free enzymes but also to lower denpol recovery yields. Apparently, an almost complete removal of unbound enzyme molecules by repetitive ultrafiltration came at relatively high costs of conjugate yield. The recovery yield of active enzymes over the whole conjugation process varied from batch to batch as well (2 – 36%). This yield could still be improved in future by less extensive purification. Remaining amounts of free enzymes could then still be washed out (with relative ease compared to the ultrafiltration process) *during the enzyme-reactor preparation* (see **Table S2** and Chapter 13). In addition, a smaller molar ratio of HRP-4FB to *de*-$PG2_{1000}$-HyNic used for the conjugation reaction could probably increase the enzyme yield as well. Besides, HRP-4FB was added in excess over *de*-$PG2_{1000}$-HyNic in this work, while in the previous investigations of Grotzky et al. (2012),[S14] no further conjugation reaction occurred upon addition of excess amounts of HRP-4FB once the conjugation reaction reached an equilibrated state (at which still ≈ 50% unreacted HyNic groups were present in the modified denpol). Due to the main goal of the present work of achieving a *controllable* enzyme immobilization, we did not try to optimize the material consumption during conjugate preparation. This should, however, be possible if required.

12. Assembly of Home-Made Flow-Through Reactor Scaffolds

The assembly of the flow-through reactor scaffolds that were used for the immobilization of the enzymes HRP and BCA was done in a similar way as in our previous work,[S10] with some modifications to make the flow-through more homogeneous and reproducible. In brief, the modifications were (i) the use of a narrower LDPE holder tube (for holding the monolith piece), (ii) the shaping of the monolith piece cross-section after cutting, and (iii) the use of a shrink tube fitting the LDPE tube tighter to the monolith piece.

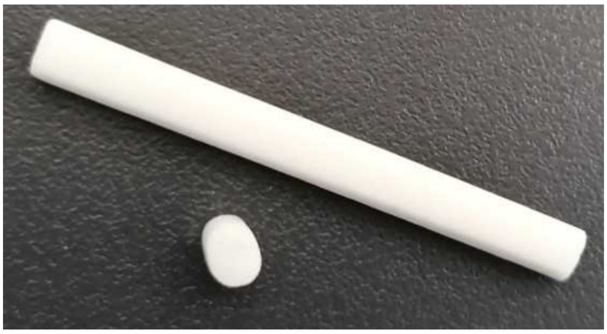

Figure S8. Photograph of a macro- and meso-porous silica monolith of the type MH1. Rod ($l_m \approx 40$ mm, $d_m \approx 4$ mm) and a cut piece thereof ($l_m = 5$ mm).

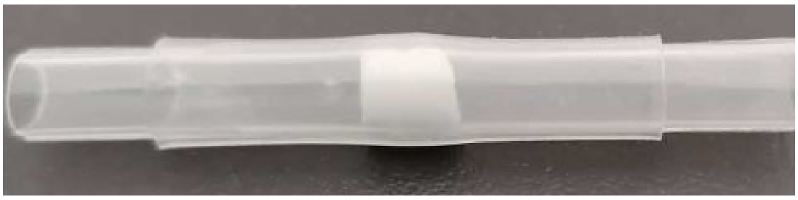

Figure S9. Photograph of a home-made flow-through reactor scaffold consisting of a piece of monolith MH1 ($l_m = 5$ mm, $d_m \approx 4$ mm) inside a LDPE tube and a shrink tube wrapped around.

Detailed reactor assembly protocol

Before use, the monolith rods of type MH1 ($l_m \approx 40$ mm, $d_m \approx 4$ mm, see Section 2.1.5.) were immersed three times in fresh ethanol for 10 min (including vortexing for the first minute to remove occasionally formed air bubbles). The monolith rods were dried with a nitrogen gun and

kept overnight at RT in a fume hood (for further drying). The dry rods were stored in a Falcon tube until further use. From the dried rods, pieces of around 7 mm length were cut by using a razor blade (for a typical final length of l_m = 5 mm), see **Figure S8**. For reactor scaffolds made of longer monolith pieces, the monolith rod was cut to an initial length of about 2 mm longer than the aimed final length. The monolith pieces were inserted carefully into the center of soft LDPE tubes (l = 4.2 cm, $d_{inner} \approx$ 4 mm, prepared from cuts of from Pasteur plast pipetes obtained from Semadeni, Switzerland, Art. Nr. 11166). As the fitting was rather tight, the monolith pieces had to be pushed to the center of the LDPE tube by a metal wrench ($d \approx$ 3 mm). With a thin metal spatula, the monolith was then shaped from both sides to achieve intersections that were as flat as possible and the monolith pieces had a desired length of l_m = 5 mm inside the tubes. Since the monolith pieces produced brittle debris upon shaping, the tube was then flushed thoroughly with the nitrogen gun. The debris need to be caught and removed carefully in a controlled way since small particles might form (work in the fume hood for that step, optimally wearing a dust mask). A transparent shrink tube (l = 2.5 cm, $d_{initial}$ = 6.4 mm) was fitted using the heat gun until no diameter decrease was observed anymore (a too close positioning of the heat gun to the shrink tube should be avoided to prevent significant deformation of the LDPE tube by melting), see **Figure S9** for an assembled reactor scaffold. After equilibriation at RT, the reactor scaffold was flushed once more with the nitrogen gun. A flow-through direction was set by drawing an arrow on the tube. The flow direction was kept the same whenever an aqueous solution was pumped through the monolith piece. Several tubes containing monolith pieces were prepared and stored in a Falcon tube until use for enzyme immobilization. Before exposure to conjugate incubation solutions, the flow-through reactor units were washed with water (see Experimental Section, 2.6.1.).

13. Reactor Washing Protocols after Conjugate Incubation and Analysis of Enzymatic Activity Eluting from the Reactor During Washing

A reactor with immobilized de-PG2$_{1000}$-BAH-HRP$_{20}$ (V_L = 50 µL, [HRP] = 500 nM) was washed with PBS at 200 µL min^{-1}. The eluate was pooled inside PP tubes in fractions of 3 mL (15 min at 200 µL min^{-1}). From eached pooled fraction 940 µL were transferred into PS cuvettes and the HRP activity was measured upon addition of 50 µL ABTS^{2-} stock solution (20 mM in PBS) and 10 µL H_2O_2 solution (20 mM in water). This analysis was done for fractions collected during 2 h, before continuing to wash the enzyme reactor overnight without further monitoring (since no activity was found in the last two pooled fractions anymore). Only 0.3% of the incubated active HRP was found in the pooled eluates. The same experiment was carried out for another monolith piece that was incubated with *de*-PG2$_{1000}$-BAH-HRP$_{40}$ (V_L = 50 µL, [HRP]$_{incubated}$ = 310 nM) and monitored for 1 h of washing, showing no HRP activity in the eluate at all. For the change in absorbance in the assays of the fractioned eluates, see **Figure S10.**

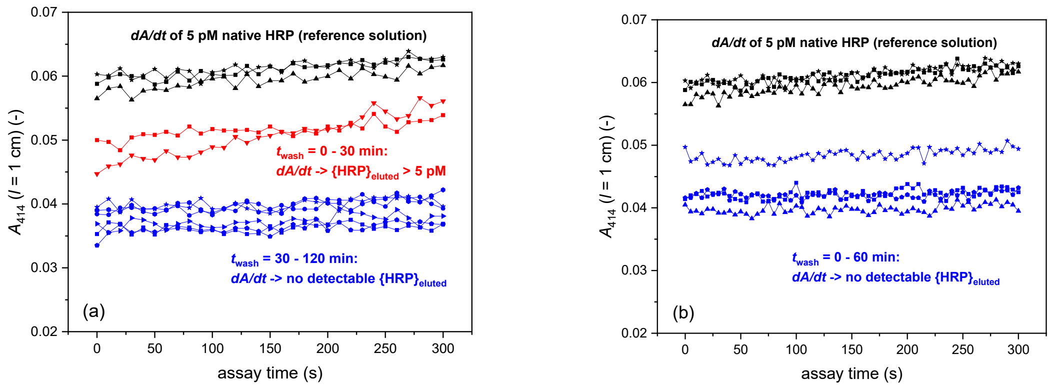

Figure S10. HRP-activity assay using ABTS^{2-}/H_2O_2 for assessing the possible presence of HRP in pooled fractions that eluted from the enzyme reactor during washing with PBS at 200 µL min^{-1} after denpol-HRP conjugate incubation for 3 h at RT. The enzyme reactors were prepared by using conjugate incubation solution consisiting of either *de*-PG2$_{1000}$-BAH-HRP$_{20}$ (V_L = 50 µL, [HRP] = 500 nM) **(a)** or *de*-PG2$_{1000}$-BAH-HRP$_{40}$ (V_L = 50 µL, [HRP] = 310 nM) **(b)**. Increase in absorbance at A_{414} indicates formation of ABTS$^{\bullet-}$. The HRP concentrations in the eluates were determined by using the calibration curve of **Figure S1b**.

A reactor with immobilized de-PG2$_{1000}$-BAH-BCA$_{54}$ (V_L = 50 µL, [BCA] = 4.1 µM) was washed with PB at 200 µL min^{-1}. PB was used instead of PBS due to a lower auto-hydrolysis of the BCA substrate PNPA, and thus to a lower detection limit for BCA in the eluate. The eluate was pooled inside PS cuvettes in fractions of 1 mL (5 min at 200 µL min^{-1}). From each pooled fraction 10 µL were removed, followed by addition of 10 µL PNPA (100 mM in acetonitrile). The PNPA auto-hydrolysis was determined by using PB that did not pass the enzyme reactor instead of the eluate. When analyzing the resulting initial rate of *p*-nitrophenolate formation (as dA_{405}/dt) − and subtracting the contribution from the auto-hydrolysis –, no significant BCA-activity was found (detection limit of 3 nM, see Chapter 2). The same experiment was carried out with another monolith piece that was incubated with *de*-PG2$_{1000}$-BAH-BCA$_{89}$ (V_L = 50 µL, [BCA] = 1.4 µM), yielding 2.4% of the incubated active BCA within the first collected fraction of the eluate but no significant activity thereafter.

14. Stability of Conjugate Stock Solutions Stored as Obtained After Purification

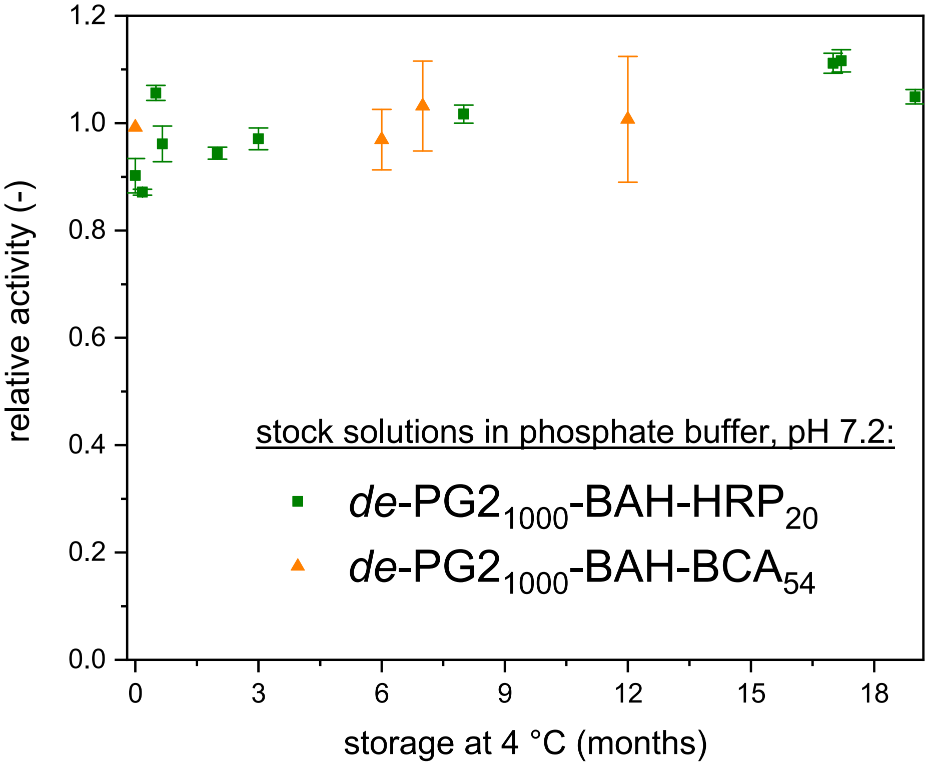

Figure S11. Relative activity of the conjugate stock solutions *de*-PG2$_{1000}$-BAH-HRP$_{20}$ (green) or *de*-PG2$_{1000}$-BAH-BCA$_{54}$ (orange), stored at 4 °C as obtained after purification at [HRP] = 2.2 µM and [BCA] = 7.4 µM in PBS* (100 mM phosphate, 1.15 M NaCl, pH = 7.2). The HRP-activity was measured using $ABTS^{2-}$ (1 mM) and H_2O_2 (0.2 mM) in PBS (100 mM phosphate, 0.15 M NaCl, pH = 7.2) by diluting aliquots to [HRP] ≈ 1 nM shortly before measurement (see 2.3.1. for the ABTS assay). The BCA-activity was measured using PNPA (1 mM) in PB (10 mM phosphate, 0 M NaCl, pH = 7.2) by diluting aliquots to [BCA] ≈ 100 nM shortly before measurement (see 2.3.2. for the PNPA assay). The error bars represent standard deviations from triplicates of three aliquots measured. Unity on the y-axis corresponded to the average activity observed for the respective conjugate ([HRP] = 2.2 ± 1 µM and [BCA] = 7.4 ± 2 µM, both as compared to a calibration curve made with native enzyme.

15. Additional SEM Images

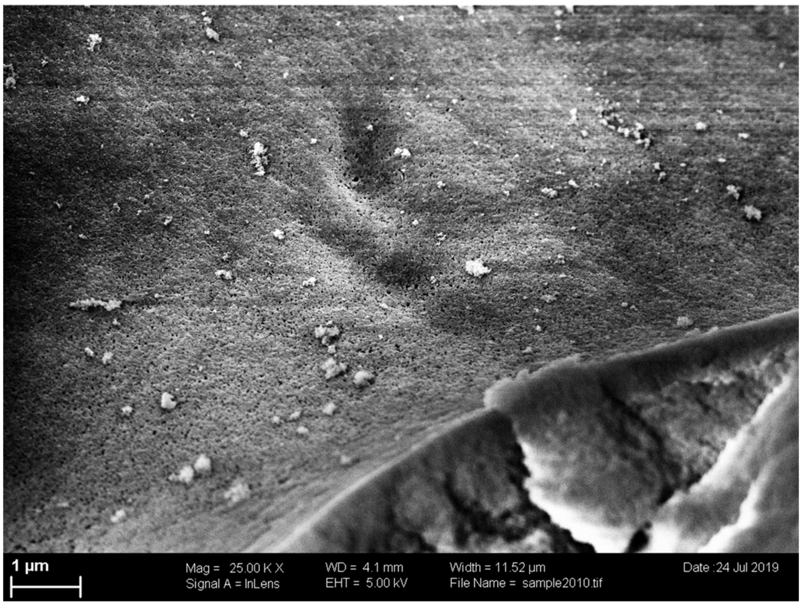

Figure S12. Surface spanned by the *macropores* of the silica monolith of type MH1. Some *mesopores* are visible on the surface as black dots. On the bottom-right, a cutting edge from the cross section of the monolith can be seen (see **Figure 4A** for a lower magnification image). The particles that are spread over the surface probably are debris produced by cutting the monolith for the SEM analysis.

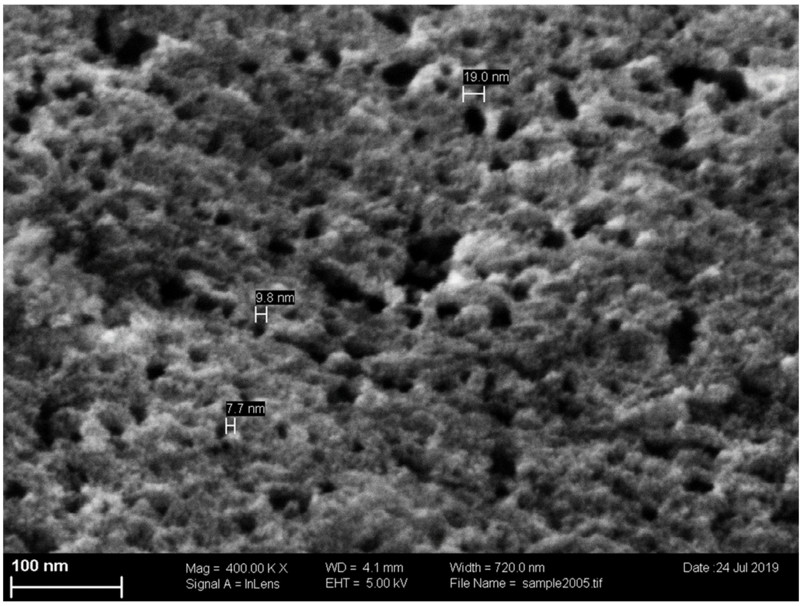

Figure S13. Zoom-in onto the surface spanned by the *macropores* of the silica monolith, type MH1 (see also **Figure S12**). The *mesopores* are clearly visible on the surface.

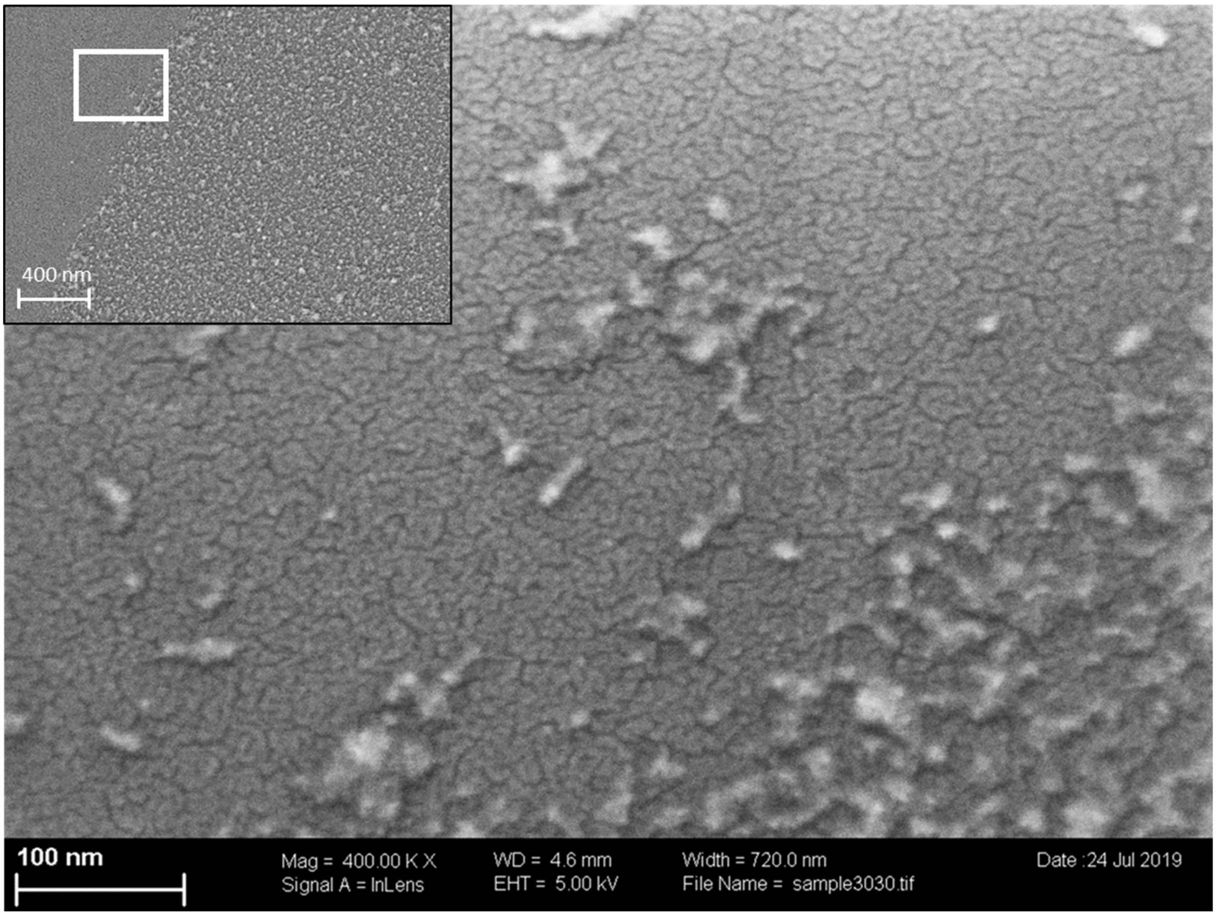

Figure S14. Flat silica cover slip surface with stably adsorbed conjugate *de*-$PG2_{1000}$-BAH-HRP_{20}. The inset figure (top-left) shows a "monolayer" formed by the conjugate (only a small fraction of overlapping conjugates) and a conjugate-free region as a reference (the surface was exposed to an aqueous conjugate solution to reach surface saturation by the conjugate; the area on the upper-left corner of the inset image is due to a scratch that occurred accidently, see **Figure 4C**). The large image is a zoom-in into the border between conjugate monolayer and free coverslip. Worm-like conjugates are visible (with an expected stretched out length of on average ≈ 250 nm). Comparison of the border region with the inner of the "monolayer" region shows that partial conjugate overlapping exists, but no large clumps of conjugates are present. The black lines visible on the cover slip surface are from small cracks in the layer of the 3 nm Pt-coating applied before measurement.

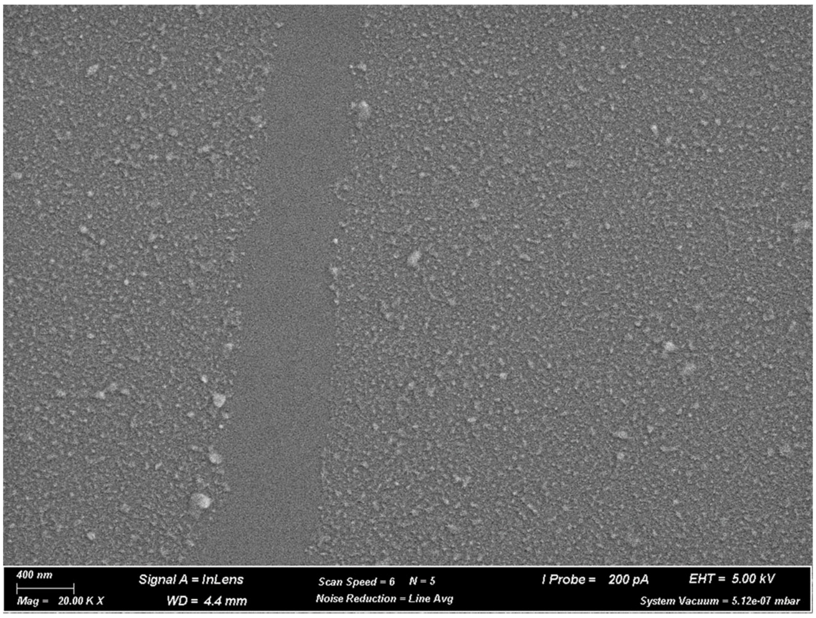

Figure S15. Flat silicate cover slip surface saturated with stably adsorbed conjugate *de*-$PG2_{1000}$-BAH-BCA_{54}. A conjugate-free region intermitting the adsorbed "monolayer" shows the same cover slip surface as observed for plain reference cover slips. Comparison of the border region with the inner of the "monolayer" shows that some partial conjugate overlapping exists, but conjugate clumps are not present.

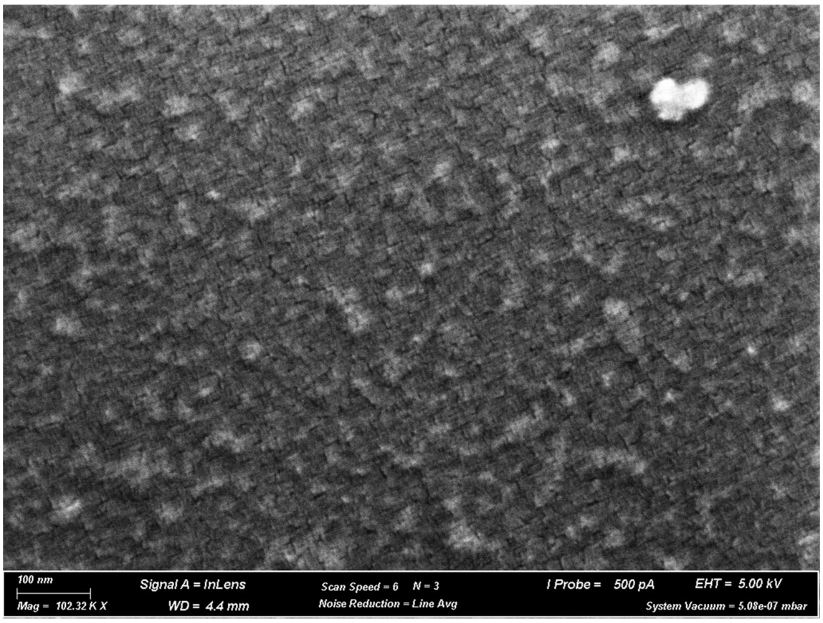

Figure S16. Zoom-in onto the adsorbed "monolayer" of the conjugate *de*-$PG2_{1000}$-BAH-BCA_{54} on a saturated cover slip (see **Figure S15**). In comparison to the corresponding image made with *de*-$PG2_{1000}$-BAH-HRP_{20} (**Figure 4D)**, it was more difficult to focus the zoomed-in image and the conjugate peripheries were less confined (maybe due to the smaller size of the enzyme BCA as compared to HRP).

16. Defensive Estimation of the Maximal Silica Surface Coverage by Conjugates *de*-PG2$_{1000}$-BAH-enzyme$_y$ as Non-Overlapping Monolayer

The (two-dimensional) area occupancy of adsoprbed denpol-enzyme conjugates of the type *de*-PG2$_x$-BAH-enzyme$_y$ forming an ideal monolayer on a flat silica surface was estimated. Assuming complete absence of conjugate overlapping, the area occupancy by the conjugates was calculated generously to later on obtain a defensively estimated maximal conjugate repeating unit (r.u.) concentration in the conjugate incubation solution to achieve complete conjugate adsorption on the surface. This estimation is specific for the r.u. of any type of *de*-PG2$_x$-BAH-enzyme$_y$, *i.e.*, independent of the number of enzymes per chain and the type of enzyme (HRP or BCA in this case).

The area occupancy of a 100 r.u. cutout of a conjugate was approximated as a rectangle of 25 nm × 15 nm for *de*-PG2$_x$-BAH-HRP$_y$ (and 25 nm × 14 nm for *de*-PG2$_x$-BAH-BCA$_y$). Schematic conjugate representations are given below, see the spatial simulations reported in *Fig. 1* of Ghéczy et al. (2020).[S11] Considering the (larger) HRP conjugate, the space requirement was estimated to 3.75×10^{-18} m^2 r.u.$^{-1}$ (25 nm × 14 nm / 100 r.u.), corresponding to a maximal surface coverage of **[r.u.]$_{max}$ = 44 pmol r.u. cm^{-2}**.

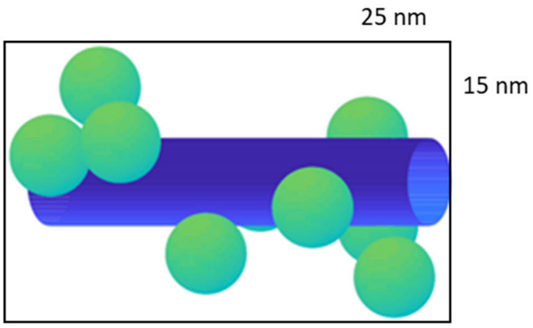

100 r.u. of *de*-PG2-BAH-HRP$_x$

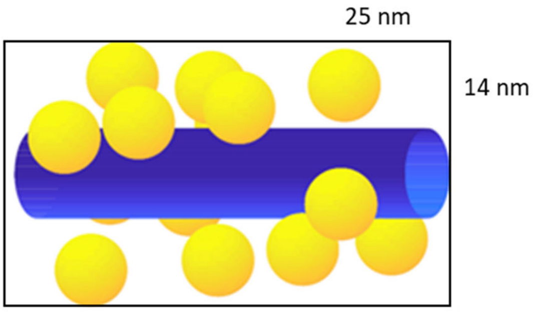

100 r.u. of *de*-PG2-BAH-BCA$_x$

In these simulations, HRP and BCA were approximated as spheres with r_{HRP} = 2.3 nm and r_{BCA} = 2.0 nm, *de*-PG2$_x$ as a cylinder with r_{denpol} = 2.5 nm and the BAH bond as two bonds of 0.5 nm length each with some rotational freedom.[S11] Note that the maximal width of the conjugate was considered independent of the number of conjugated enzymes per chain. It was taken as r.u. with two bound enzymes that are placed exactly in opposite positions to each other. For estimating the length of an extended, *i.e.*, uncoiled *de*-PG2$_x$ chain, a value of 0.25 nm per repeating unit (r.u.) was taken into account. See Electronic Supporting Information (Section 6) of Ghéczy et al. (2020)[S11] for details.

Estimated maximal conjugate r.u. concentration that can be used in conjugate incubation solutions for maximal conjugate coverage inside the silica monolith MH1. Taking into account the internal surface and volume of the monolith MH1 that is accessible for the conjugate solutions (determined by the macropores), $S_{macropores}$ = 0.72 m^2 g^{-1} (= 1.8 × 10^5 m^{-1} monolith length) and V_L = 4 cm^3 g^{-1} (see Section, 2.1.5.), the maximal surface coverage of 44 pmol r.u. cm^{-2} (as estimated above), corresponds to a defensively estimated maximal conjugate r.u. concentration in the conjugate incubation solution of [r.u.] = 79.2 μM. This concentration was never exceeded when exposing monolith pieces of length l_m, diameter d_m, and volume V_L to conjugate incubation solutions for immobilization of the conjugates in the preparation of the enzyme reactors for flow-through applications ($[r.u.]_{incubated} < [r.u.]_{max}$ = 80 μM, see Section 3.2.1.).

17. On the Detection of Enzyme Molecules that Leaked from the Enzyme-Reactor

For a determination of the enzyme immobilization yield based on the activity measurements of enzyme molecules that leaked from the enzyme reactor − after incubation with a conjugate solution − and would appear in the eluent after washing with buffer solution, it is assumed that these leaked enzyme molecules are catalytically active (either as part of leaked conjugates or as free enzymes). This assumption is reasonable and was supported by the following experiment.

In our previous investigation,[S11] in which the enzyme immobilization inside the silica monolith MH1 was not yet optimized, active enzyme molecules that leaked from a monolith piece which was exposed to an aqueous solution of a denpol-HRP conjugate, could clearly be detected, see **Figure S17**. The conjugate prepared and used was *de*-PG2_{1000}-BAH-HRP$_{17}$, see also Chapter 4.2.[S11] This conjugate was incubated in the previous work in a monolith piece for 1 h at pH = 5 and then washed with a buffer solution of pH = 7 (see Hou et al., 2019, parts using *de*-PG2_{1000}-BAH-HRP$_{71}$).[S11] **Figure S17** clearly shows that continuously decreasing amounts of active enzyme molecules are present in the outflow from the enzyme reactor, yielding 4.2 % of the incubated activity eluted after 240 min (4 h) of washing. Despite the lower incubation time (1 h instead of 3 h), the higher activity eluted from the monolith piece probably came from free HRP molecules that were not completely removed during conjugate purification. See **Table S2**. To achieve high enzyme immobilization yields (close to 100%), the use of incubation solutions containing highly purified conjugates is important, see also **Figure 5**.

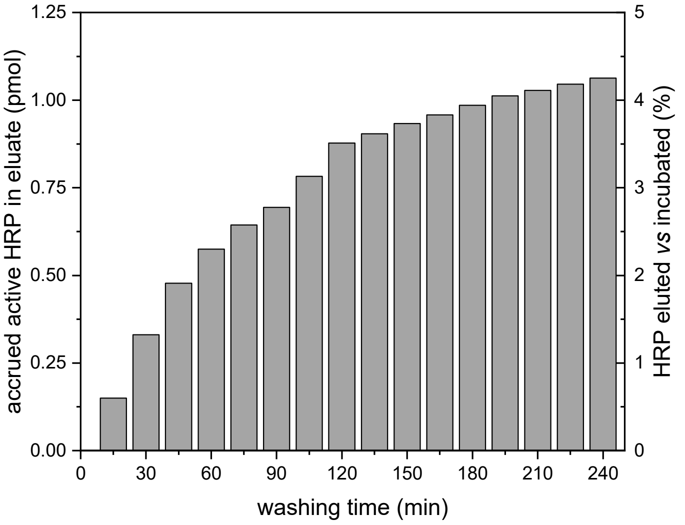

Figure S17. Accrued amounts of active HRP molecules eluted from a monolith piece during flow-through washing with buffer solution (pH = 7.0) (at 200 μL min^{-1}). The monolith piece was loaded with an aqueous solution of *de*-$PG2_{1000}$-BAH-HRP_{17} (V_L = 50 μL, [HRP] = 500 nM, pH = 5.0). The amount of active HRP in the outflow was determined with the ABTS-assay (see Section 2.3.1.).

That the reason for the prevailing absence of eluting active enzyme in this present work was not simply inactivation within the pooled fractions before substrate addition, was shown with the following experiment.

For a monolith piece that was incubated with a solution of *de*-$PG2_{1000}$-BAH-HRP_{40} (V_L = 50 μL, [HRP] = 310 nM), after 1 h of washing with PBS (at 200 μL min^{-1}), PBS was replaced by the assay solution containing 1.0 mM $ABTS^{2-}$ and 0.2 mM H_2O_2 (PBS). After a few minutes, *the flow was stopped* and the UV/vis absorption spectrum of the eluant was measured. The absorbance at λ = 414 nm (due to formed $ABTS^{\bullet -}$) remained stable (*i.e.*, no increase with time). While the reaction product concentration increased *inside the HRP reactor*, stability in that concentration in the stored outflow (*after having left the reactor*) indicated no active HRP molecules leaking out from the HRP reactor.

18. Flow-Through Assays of Enzyme Reactors Prepared From *de*-PG2$_{1000}$-BAH-HRP$_y$ or *de*-PG2$_{1000}$-BAH-BCA$_y$

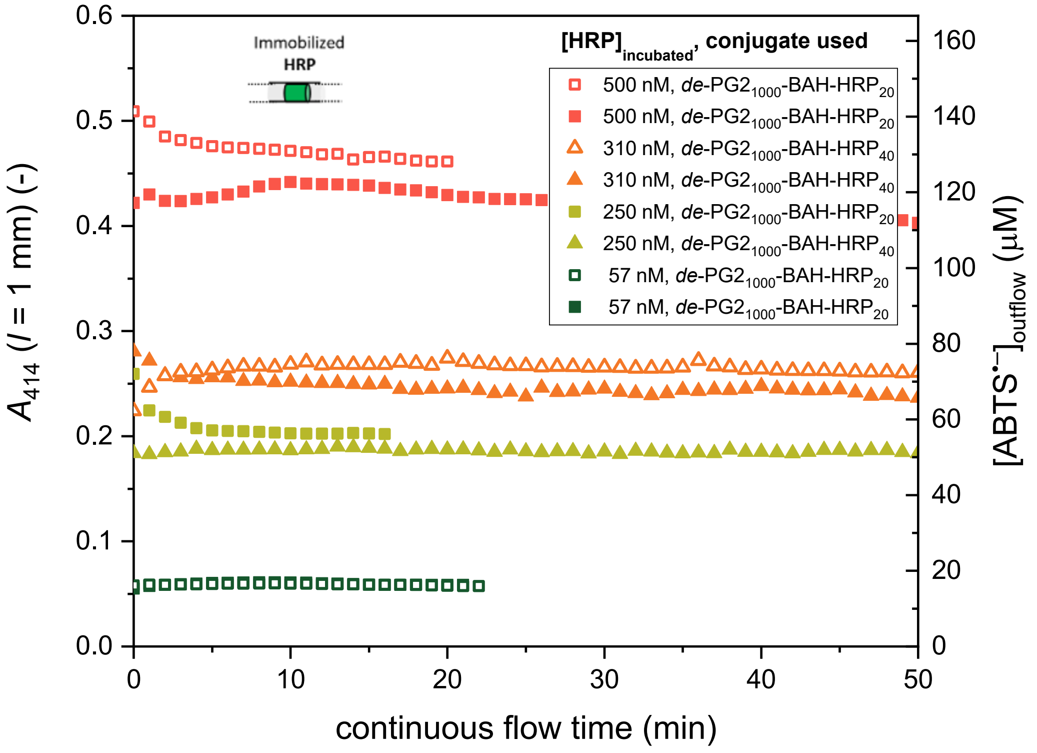

Figure S18. Online activity measurements for enzymatic flow-through reactors containing immobilized *de*-PG2$_{1000}$-BAH-HRP$_y$. Several enzyme reactors of the same size (l_m = 5 mm, $d_m \approx$ 4 mm, V_L = 50 µL) were prepared using incubation solutions of the conjugates *de*-PG2$_{1000}$-BAH-HRP$_{20}$ (squares) or *de*-PG2$_{1000}$-BAH-HRP$_{40}$ (triangles) at different concentrations ([HRP] = 57 – 500 nM). Empty symbols represent data for a second enzyme reactor prepared in the same way. The substrate solution ([ABTS^{2-}]$_0$ = 1.0 mM; [H_2O_2]$_0$ = 0.2 mM; in PBS (100 mM phosphate, 150 mM NaCl, pH = 7.2) was passed through the enzyme reactors at a flow rate of 200 µL min^{-1} (1.6 mL min^{-1} cm^{-2}). Using ε_{414} (ABTS$^{\bullet-}$) = 36 000 M^{-1}cm^{-1} (Childs and Bardsley (1975)),[S1] the product concentration in the outflow was calculated, [ABTS$^{\bullet-}$]$_{outflow}$. For details about the ABTS flow-through assay, see Experimental Section 2.8.2.

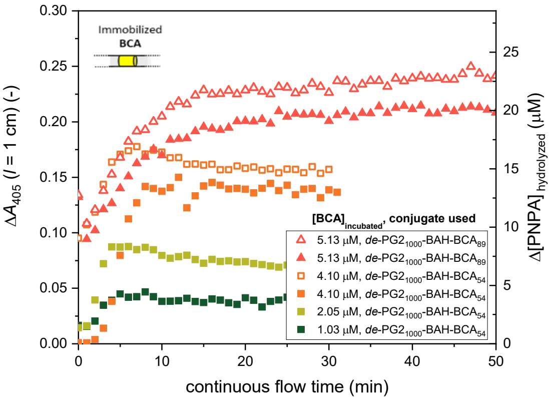

Figure S19. Online activity measurements for enzymatic flow-through reactors containing immobilized *de*-PG2$_{1000}$-BAH-BCAy. Several reactors of the same size ((l_m = 5 mm, $d_m \approx$ 4 mm, V_L = 50 μL) were prepared using incubation solutions of the conjugates *de*-PG2$_{1000}$-BAH-HRP$_{54}$ (squares) or *de*-PG2$_{1000}$-BAH-HRP$_{89}$ (triangles) at different concentrations ([BCA] = 1.03 – 5.13 μM). Empty symbols represent data for a second enzyme reactor prepared in the same way. The substrate solution ([PNPA]$_0$ = 1.0 mM; in PB (10 mM phosphate, pH = 7.2) was passed through the enzyme reactors at a flow rate of 200 μL min^{-1} (1.6 mL min^{-1} cm^{-2}). Changes in the absorbance at λ = 405 nm were assigned to an increase in product concentration caused by enzymatic catalysis (ΔA_{405}, as compared to a control experiment using a reactor without immobilized enzymes to subtract the contribution from non-enzymatic hydrolysis, see **Figure S20**). Using ε_{405} (*p*-nitrophenol/*p*-nitrophenolate at pH = 7.2) = 10 510 M^{-1} cm^{-1} (Innocenti et al. (2008)),[S3] the product formation was also expressed in terms of extent of substrate hydrolysis as Δ[PNPA]$_{hydrolyzed}$. For details about the PNPA flow-through assay, see Experimental Section 2.8.3.

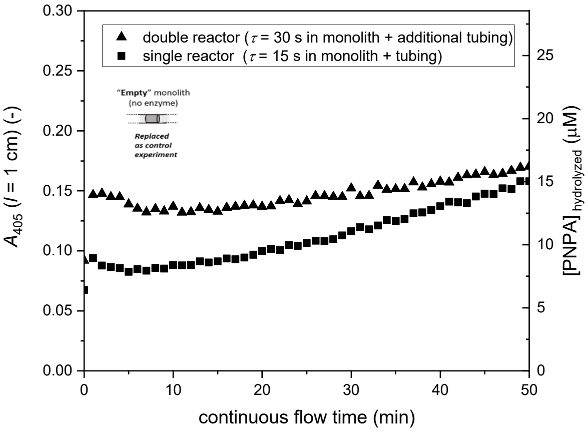

Figure S20. PNPA auto-hydrolysis when passing a PNPA solution through a piece of the silica monolith MH1. The same type of experiment as described in **Figure S19** was carried out, with the exception that the monolith piece did not contain enzymes. To align for the time the PNPA substrate solution stayed in the reservoir form which the solution was passed through the enzyme reactors of **Figure S19**, the first spectrum in the flow-through cell was always taken 5 or 8 min after preparation of the substrate solution (in the case of a single reactor or two sequentially connected reactors, respectively). For details about the PNPA flow-through assay, see Experimental Section 2.8.3.

19. Flow-Through Assays of Enzyme Reactors Depending on Residence Time or Enzyme Distribution

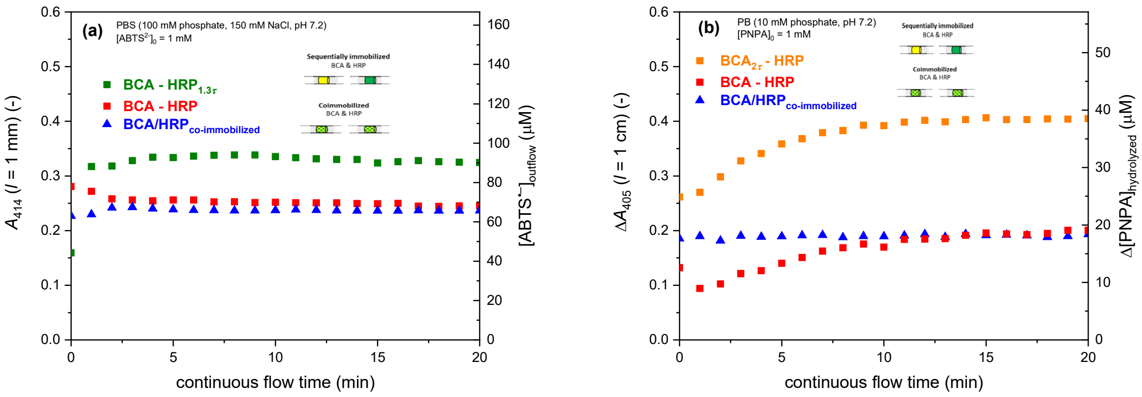

Figure S21. Online activity measurements for enzymatic flow-through reactor systems containing immobilized *de*-PG2_{1000}-BAH-BCA$_{89}$ and *de*-PG2_{1000}-BAH-HRP$_{40}$. For the conditions used for the preparation of the reactors systems, either with *sequentially* (red) or *co-immobilized* (blue) conjugates, see Experimental Sections 2.6.3 or 2.6.4, respectively. The activity of the immobilized enzymes was measured by using substrate solutions that were pumped through the enzyme reactor systems at a flow rate of 200 µL min^{-1} (1.6 mL min^{-1} cm^{-2}). The product formation was found to linearly increase with increase in residence time and with increasing amounts of immobilized enzymes.

(a) Substrate solution pumped through the enzyme reactor systems at 200 µL min^{-1}: $[ABTS^{2-}]_0$ = 1.0 mM; $[H_2O_2]_0$ = 0.2 mM; in PBS (100 mM phosphate, 150 mM NaCl, pH = 7.2). Using ε_{414} ($ABTS^{\bullet-}$) = 36 000 M^{-1}cm^{-1} (Childs and Bardsley (1975)),[S1] the product concentration in the outflow was calculated, $[ABTS^{\bullet-}]_{outflow}$. "HRP$_{1.3\tau}$" means that the enzyme reactor with immobilized HRP was 1.3-times longer than usual. For details about the ABTS flow-through assay, see Experimental Section 2.8.2.

(b) Substrate solution pumped through the enzyme reactor systems at 200 µL min^{-1}: $[PNPA]_0$ = 1.0 mM; in PB (10 mM phosphate, pH = 7.2). Changes in the absorbance at λ = 405 nm were assigned to an increase in product concentration caused by enzymatic catalysis (ΔA_{405}, as compared to a control experiment using a reactor without immobilized enzymes to subtract the contribution from non-enzymatic hydrolysis, see **Figure S20**). Using ε_{405} (*p*-nitrophenol/*p*-nitrophenolate at pH = 7.2) = 10 510 M^{-1} cm^{-1} (Innocenti et al. (2008)),[S3] the product formation was also expressed in terms of extent of substrate hydrolysis as $\Delta[PNPA]_{hydrolyzed}$. "BCA$_{2\tau}$" means that the enzyme reactor with immobilized BCA was 2-times longer than usual. For details about the PNPA flow-through assay, see Experimental Section 2.8.3.

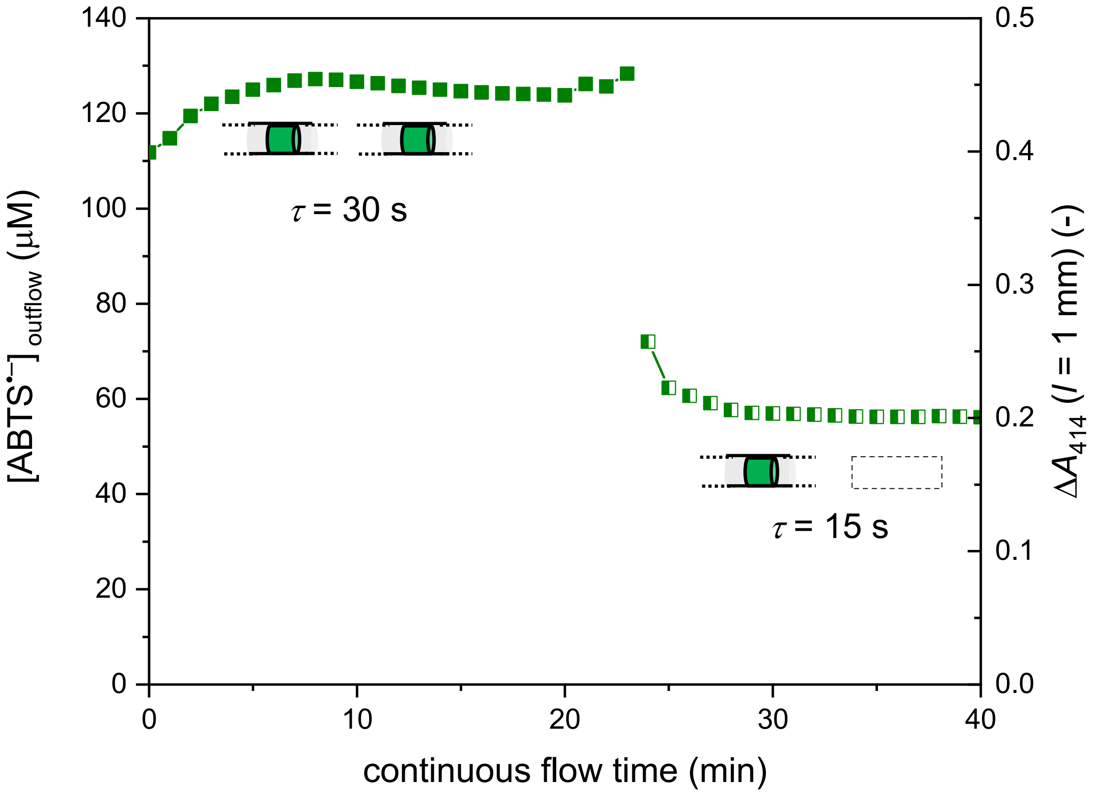

Figure S22. Online activity measurements for two connected flow-through HRP reactors containing immobilized *de*-PG2$_{1000}$-BAH-HRP$_{20}$ (each l_m = 5 mm, d_m ≈ 4 mm, V_L = 50 µL, immobilized at [HRP]$_{incubated}$ = 250 nM). The substrate solution that was pumped through the two connected enzyme reactors at 200 µL min^{-1} (1.6 mL min^{-1} cm^{-2}) consisited of [ABTS^{2-}]$_0$ = 1.0 mM; [H_2O_2]$_0$ = 0.2 mM; in PBS (100 mM phosphate, 150 mM NaCl, pH = 7.2). Using ε_{414} (ABTS$^{\bullet-}$) = 36 000 M^{-1}cm^{-1} (Childs and Bardsley (1975),[S1] the product concentration in the outflow was calculated, [ABTS$^{\bullet-}$]$_{outflow}$. For details about the ABTS flow-through assay, see Experimental Section 2.8.2. For the data points shown on the right hand side, the two initially connected HRP reactors - through which the substrate solution was pumped through - were separated after about 20 min of flow. The second HRP reactor was removed and replaced by respective tube. Then substrate solution was pumped though the remaining HRP reactor again at 200 µL min^{-1}. As a result, the product concentration in the outflow was approximately halved.

20. Determination of the HRP and BCA Activity Recovery upon Immobilization of *de*-PG2$_{1000}$-BAH-HRP$_y$ or *de*-PG2$_{1000}$-BAH-BCA$_y$ Conjugates in Monolith Pieces Under Flow-Through Conditions

With the immobilization method used in this work, the amount of enzyme molecules immobilized inside a monolith piece of defined length (usually l_m = 5 mm, $d_m \approx$ 4 mm, V_L = 50 µL) is given by the amount of enzyme molecules present in the conjugate incubation solution which was added to the monolith piece (V_L = 50 µL for the usual monolith piece), *i.e.*, the *enzyme immobilization yield* is ≈ 100%. In other words, almost all denpol-enzyme conjugates added to the monolith piece remained immobilized inside the monolith piece. For determining the *activity recovery*, the activity of the immobilized enzyme molecules is compared with the activity of the enzyme molecules in bulk solution by using one and the same substrate solution and determining initial rates of substrate conversion (v_{in}). This can be done, for example, by comparing observed rate constants, k_{obs} (s^{-1}). *For bulk solution measurements,* k_{obs} (bulk solution) is obtained by dividing v_{in} (M s^{-1}) by the molar enzyme concentration [E] (M), *i.e.*, k_{obs} (bulk solution) = v_{in} $[E]^{-1}$. *For enzymatic flow-through reactor measurements*, the time the substrate solution needs to pass through the monolith, the residence time, τ (s), needs to be taken into account. For the standard monolith (l_m = 5 mm, $d_m \approx$ 4 mm, V_L = 50 µL) and a flow rate of 200 µL min^{-1}, τ = 15 s. Regarding τ and the product concentration of the outflow from the enzyme reactor under steady-state (*and initial reaction rate*) conditions, $[P]_s$, the activity per immobilized enzyme (≈ enzyme used for immobilization in our case) was calculated as rate constant, *i.e.*, k_{obs} (immobilized) = $[P]_s$ τ^{-1} $[E]^{-1}$.

HRP and BCA activity recoveries determined with the ABTS- and PNPA-assay, respectively, are given in **Table 1**, as calculated from data shown in **Figure 5**. HRP activity recovery values determined with the cascade reaction using DCFH$_2$-DA (50 µM) and H_2O_2 (30 µM) as substrates

are listed in **Table S3**; and BCA activity recovery values determined with $DCFH_2$-DA as substrate are shown in **Table S4.**

For the case of the cascade reaction, the HRP-catalyzed oxidation was analyzed by either following the increase in A_{460} with time (reaction in bulk solution), or by determining A_{460} in the outflow from the enzyme reactors between 4 and 5 h after starting to pump the substrate solution through the enzyme reactors (flow-through analysis, as described in Section 2.8.4.). The absorption at λ_{iso} = 460 nm reflects the formation of DCF-MA + DCF (ε_{460} (DCF-MA/DCF, pH = 7.2) = 19800 $M^{-1}cm^{-1}$).[S11] Due to the low flow rate used (5 µL min^{-1}), the residence time, τ, was 10 - 20 min, see **Table S3** for details.

Table S3. Activity recovery upon immobilization of *de*-$PG2_{1000}$-BAH-HRP_{40}, measured with different enzyme reactor systems using the cascade flow-through reaction ($[DCFH_2\text{-}DA]_0$ = 50 µM, $[H_2O_2]_0$ = 30 µM, PBS, pH = 7.2).

Enzyme Reactor Systems	Residence Time (τ)[a] *(min)*	A_{460}[b] (l = 1 cm) *(-)*	[HRP] of conjugate incubation solution *(nM)*	k_{obs}[d] (immobilized) *(s^{-1})*	Activity Recovery[e] *(%)*
BCA & $HRP_{co\text{-}immobilized}$	20	0.101±0.003	155	0.027±0.001	**61±2**
BCA - HRP	10	0.101±0.002	310	0.028±0.001	**61±1**
BCA - $HRP_{1.3\tau}$[c]	13	0.130±0.001	310	0.027±0.000	**61±1**
$BCA_{2\tau}$ - HRP[c]	10	0.101±0.004	310	0.028±0.001	**61±2**

[a] Only considering monolith pieces containing HRP.
[b] Standard deviation from three collected outflows.
[c] Longer residence time for longer HRP reactors (same enzyme concentration in conjugate incubation solution used)
[d] Specific activity per immobilized enzyme as observed rate constant, k_{obs} (immobilized) = $[P]_s \tau^{-1} [E]^{-1}$, see text.
[e] Calculated by comparing the obtained k_{obs} (immobilized) to k_{obs} (bulk solution). Conditions for bulk solution measurements to which the HRP activity recovery is related: $[H_2O_2]_0$ = 30 µM, [HRP] = 100 nM, [BCA] = 1.5 µM, PBS, pH = 7.2. k_{obs} (bulk solution) = 0.045 s^{-1}, see later Chapter 24 and **Figure S26**.

For determining the BCA activity recovery by using the cascade reaction, the BCA-catalyzed hydrolysis of $DCFH_2$-DA was analyzed by following the decrease in A_{260} and increase in A_{287} (in

bulk solution measurements), or by quantifying the remaining $DCFH_2$-DA concentration in the outflow collected 5 h after starting to pump the substrate solution through the enzyme reactor systems (enzymatic flow-through reactor measurements). From the bulk solution measurements, the catalytic efficiency was determined for native BCA (k_{cat}/K_M = 0.0130 μM^{-1} min^{-1}, see later **Figure S24**). With this value, an *apparent concentration of active BCA* inside the reactor during flow-through, $[BCA]_{app}$, was estimated by using first-order kinetics with respect to the decreasing substrate concentration ($[DCFH_2\text{-}DA]_\tau = [DCFH_2\text{-}DA]_0 \exp(k_{cat}/K_M\ \tau\ [BCA]_{app})$). $[BCA]_{app}$ was only used for numerical comparison to [BCA] in the conjugate incubation solution used for reactor preparation and *did not represent an actual BCA concentration*. The concentration of BCA in the conjugate incubation solution used in the immobilization step, [BCA], was determined with the PNPA-assay, see Seecion 2.3.2. All data are given in **Table S4**.

Table S4. Activity recovery upon immobilization of *de*-$PG2_{1000}$-BAH-BCA_{89}, measured with different enzyme reactor systems using the cascade flow-through reaction ($[DCFH_2\text{-}DA]_0$ = 50 µM, $[H_2O_2]_0$ = 30 µM, PBS, pH = 7.2).

Reactor Setup	Residence Time (τ)[a] (min)	Remaining $DCFH_2$-DA (µM)	$[BCA]_{app}$[b] (µM)	[BCA] of conjugate incubation solution (µM)	Activity Recovery[d] (%)
BCA + $HRP_{co\text{-}immobilized}$	20	29.3	1.33	2.57	**52**
BCA - HRP	10	29.7	2.56	5.13	**50**
BCA - $HRP_{1.3\tau}$[c]	10	29.2	2.69	5.13	**53**
$BCA_{2\tau}$ - HRP[c]	20	20.5	2.71	5.13	**53**

[a] Only considering monolith pieces containing BCA.
[b] Calculated from $[DCFH_2\text{-}DA]_\tau = [DCFH_2\text{-}DA]_0 \exp(k_{cat}/K_M\ \tau\ [BCA]_{app})$, for comparison to [BCA] of the conjugate incubation solution, not an actual concentration of BCA, see text. The calculation was started with $[DCFH_2\text{-}DA]_0$ = 41.5 µM (instead of 50 µM) as obtained from the outflow of empty monoliths in the same type of experiment (see later **Table S5** for the auto-hydrolysis fo $DCFH_2$-DA in PBS). Conditions for bulk solution measurements for determining k_{cat}/K_M: [BCA] = 1 µM, $[DCFH_2\text{-}DA]_0$ = 50 µM, PBS, pH = 7.2.
[c] Longer residence time for longer BCA reactors (same enzyme concentration in conjugate incubation solution used)
[d] Calculated as $[BCA]_{app}$ (reactor) / [BCA] (incubation solution). Same effective volume during incubation and assay.

21. Operational Stability of a Reactor Containing Immobilized *de*-PG2$_{1000}$-BAH-HRP$_{20}$

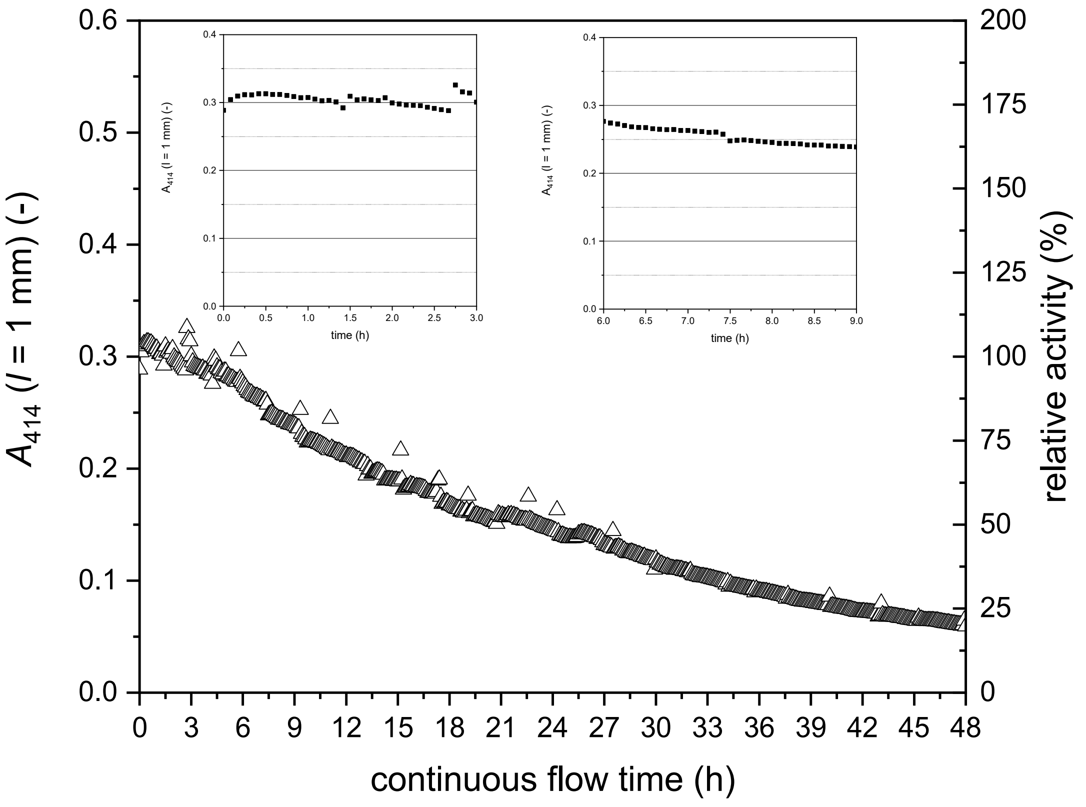

Figure S23. Online monitoring of the performance of a flow-through HRP reactor containing immobilized *de*-PG2$_{1000}$-BAH-HRP$_{20}$ (l_m = 5 mm, $d_m \approx$ 4 mm, V_L = 50 µL, [HRP] = 500 nM). After preparing the HRP reactor, it was first stored for 2 weeks at 4 °C (immersed in PBS), with concomitant loss of about 30% of its HRP activity, see **Figure 7A**. The reactor was then exposed for 48 h to a continuous flow of substrate solution ($[ABTS^{2-}]_0$ = 1.0 mM; $[H_2O_2]_0$ = 0.2 mM; PBS, pH = 7.2) at a flow rate of 200 µL min^{-1} (1.6 mL min^{-1} cm^{-2}). A_{414} reflects the HRP-catalyzed formation of $ABTS^{\bullet-}$ (ε_{414} ($ABTS^{\bullet-}$) = 36 000 M^{-1}cm^{-1}, see Childs and Bardsley (1975)).[S1] The insets are zoom-ins of the outflow during the first 3 hours and after 6 - 9 hours after starting to pump the substrate solution through the HRP reactor.

22. The Applied Two-Enzymes Cascade Reaction Involving Two Reaction Pathways

The cascade reaction applied in this work is summarized in **Figure 3**, see also Ghéczy et al. (2020).[S11] DCFH$_2$-DA is first hydrolyzed to DCFH$_2$-MA (catalyzed by BCA, *Hyd_1*). When all components are present from the very beginning, the reaction can proceed along two possible pathways; either *via* a further BCA-catalyzed hydrolysis to DCFH$_2$ (*Hyd_2*) or *via* a HRP-catalyzed oxidation by H_2O_2 to DCF-MA (*Oxi_2*), following *Pathway 1* or *2*, respectively. In both cases, subsequent reaction steps result in the formation of DCF, the final component. Which pathway the reaction follows predominantly depends on the relative activities of the two enzymes. This includes not only their specific activity for catalyzing the reaction steps and the initially added amounts of enzymes and substrates (*i.e.*, their concentrations), but also depends on the reaction time (due to changes of the substrate concentrations as the reaction proceeds). The BCA-catalyzed hydrolysis of DCFH$_2$-MA was found to be slower than the BCA-catalyzed hydrolysis of DCFH$_2$-DA (*Hyd_1* > *Hyd_2*, according to the determined k_{cat}/K_M values). Consequently, *pathway 2* will always be predominant in a first phase of the cascade reaction. If HRP is used at a rate limiting concentration, DCFH$_2$-MA accumulates, which results not only its HRP-catalyzed oxidation to DCF-MA, but also in its BCA-catalyzed hydrolysis to DCFH$_2$. If enough time is given, *pathway 1* becomes dominant since DCFH$_2$ was found to be oxidized easier by HRP/H_2O_2 than DCFH$_2$-MA (*Oxi_1* > *Oxi_2* given similar concentrations of the two oxidizable intermediates). Therefore, *pathway 1* could eventually become dominant in a second phase of the cascade reaction. For quantitative relations between the reaction steps, see Section 3.4.1. and the previous work of Ghéczy et al. (2020),[S11] where details about the reaction mechanism are also discussed.

23. BCA-Catalyzed Hydrolysis of $DCFH_2$-DA, $DCFH_2$-MA and DCF-MA at pH = 7.2

The BCA-catalyzed hydrolyses of $DCFH_2$-DA, $DCFH_2$-MA, and DCF-MA were investigated in PBS (100 mM NaH_2PO_4, 150 mM NaCl, pH = 7.2). Note that in our previous work, the same hydrolysis reactions were investigated in PB (10 mM NaH_2PO_4, 0 mM NaCl, pH = 7.2), see Ghéczy et al. (2020).[S11] All reactions followed first-order kinetics, $[S]_t = [S]_0 \cdot e^{-kt}$, and they were independent from each other (due to $[S]_0 \ll K_M$, see Table S-2 in Ghéczy et al. (2020)).[S11] Assuming a negligible contribution of the non-enzymatic, buffer-mediated background reaction ("auto-hydrolysis"), the relation $k = (k_{cat}/K_M) \cdot [BCA]_0$ applies. In this work, we considered a contribution from the non-enzymatic hydrolysis of the different substrates by PBS due to the higher phosphate concentration than in the case of PB, expressed as rate constant k_{buffer}. In this case, the relation $k = (k_{cat}/K_M) \cdot [BCA]_0 + k_{buffer}$ was applied. The catalytic efficiencies (k_{cat}/K_M) and the "auto-hydrolysis" rate constants (k_{buffer}) are summarized in **Table S5**.

Table S5. Catalytic activities (k_{cat}/K_M) and buffer-induced auto-hydrolysis rate constants (k_{buffer}) observed for the BCA-catalyzed hydrolysis reaction in PBS (100 mM NaH_2PO_4, 150 mM NaCl, pH = 7.2) and comparison to values obtained in PB (10 mM NaH_2PO_4, 0 mM NaCl, pH = 7.2).

Hydrolyzed BCA-Substrate	$DCFH_2$-DA in PBS (Hyd_1)	$DCFH_2$-MA in PBS (Hyd_2)	DCF-MA in PBS (Hyd_3)	$DCFH_2$-DA in PB[c] (Hyd_1)	$DCFH_2$-MA in PB[c] (Hyd_2)	DCF-MA in PB[c] (Hyd_3)
k_{cat}/K_M (μM^{-1} min^{-1})	0.0130	0.0017	0.0033 (±0.0003)	0.0198	0.0012	0.0047
k_{buffer}[a] (min^{-1})	0.00046	-	0.0056[b]	0.00014	-	-

[a] When the phosphate-mediated "auto-hydrolysis" was not determined, it was assumed to be negligibly small compared to the enzymatic hydrolysis using $[BCA]_0 \approx 1$ µM. Upon calculating k_{cat}/K_M it became clear whether the contribution by the phosphate ions (k_{buffer}) had to be taken into account.

[b] Quantified in an enzyme-free DCF-MA/DCF mixture, as obtained from flow-through experiments with immobilized enzymes, see text.

[c] Data from Ghéczy et al. (2020)[S11]

For the BCA-catalyzed hydrolysis of $DCFH_2$-DA in PBS, **Figure S24** shows time-dependent UV/vis absorption spectra of the reaction mixture and calculated changes of the concentrations of $DCFH_2$-DA, $DCFH_2$-MA and $DCFH_2$. By exponential fitting, the catalytic efficiencies for the three BCA-catalyzed hydrolysis reactions were calculated. This was done in the same way as in our previous work,[S11] with the exception that we now also resolved the concentrations of $DCFH_2$-MA and $DCFH_2$ during the initial reaction phase in which $DCFH_2$-DA was still present. This allowed to predict along which pathway the cascade reaction is expected to proceeded if HRP and H_2O_2 are present as well (*i.e.*, oxidation of $DCFH_2$-MA and/or $DCFH_2$).

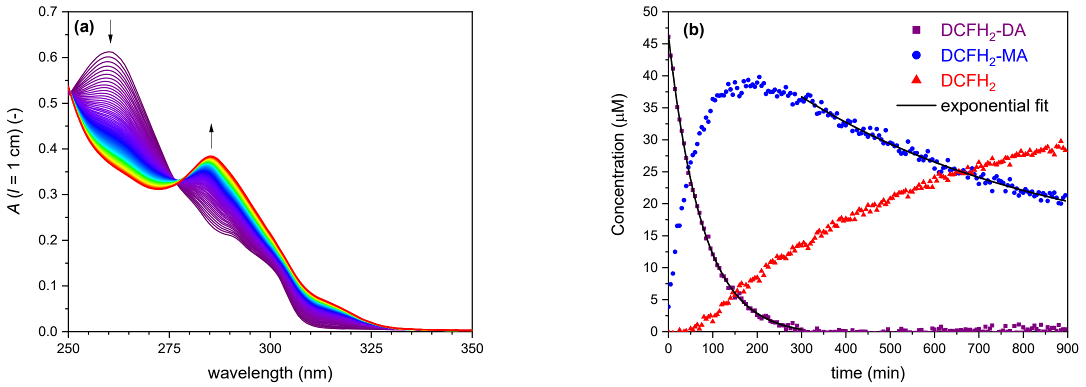

Figure S24. BCA-catalyzed hydrolysis of $DCFH_2$-DA to $DCFH_2$-MA and the follow-up hydrolysis of $DCFH_2$-MA to $DCFH_2$. BCA (1 µM) and $DCFH_2$-DA (50 µM, 1 vol% DMSO) were mixed in PBS (100 mM NaH_2PO_4, 150 mM NaCl, pH = 7.2) and UV/vis absorption spectra were recorded every 5 min for a duration of 15 h at 25 °C.
(a) The full spectra recorded are shown. The decrease of A_{260} and the increase of A_{287} indicate the hydrolysis of $DCFH_2$-DA to $DCFH_2$-MA and further to $DCFH_2$. At the beginning of the reaction (purple lines), the isosbestic point between $DCFH_2$-DA and $DCFH_2$-MA is located at λ_{iso} = 276 nm, while in the later stage (blue to red lines), the isosbestic point for $DCFH_2$-MA and $DCFH_2$ is seen at λ_{iso} = 279 nm. For the molar absorptions of the three components, see Ghéczy et al. (2020).[S11]
(b) The concentrations were calculated based on the molar absorptions at the characteristic wavelengths and isosbestic points mentioned in **(a)**. From the shape of the three curves, it is evident that the initial hydrolysis reaction was much faster than the follow-up hydrolysis reaction. Within the first 60 min, barely any $DCFH_2$ formed. The catalytic efficiency was fit using first-order kinetics for the hydrolysis of $DCFH_2$-DA within t = 0 – 300 min (k_{cat}/K_M = 0.0130 μM^{-1} min^{-1}, R^2 = 0.9992) and for the hydrolysis of $DCFH_2$-MA within t = 300 – 900 min (k_{cat}/K_M = 0.0017 μM^{-1} min^{-1}, R^2 = 0.9816). In both cases, any potential non-enzymatic contribution to the measured hydrolysis rate was considered neglectable. The phosphate-mediated "auto-hydrolysis" of $DCFH_2$-DA in PBS was determined by fitting of experimental data in the same way with a $DCFH_2$-DA solution that did not contain BCA (k_{buffer} = 0.00046 min^{-1}, R^2 = 0.9937).

For the BCA-catalyzed hydrolysis of DCF-MA, the reaction conditions were the same as for the cascade reaction (see Section 2.3.3.), except that (i) DCF-DA (10 μM in PBS, 1 vol% DMSO; prepared form a 1 mM stock solution in DMSO) was used instead of $DCFH_2$-DA, (ii) HRP and H_2O_2 were absent, and (iii) the concentration of BCA was varied (0, 1 or 3 μM). The course of reaction for [BCA] = 1 or 3 μM is shown in **Figure S25**. Note that the rate constant (k) that was obtained by fitting of the experimental data was *not* proportional to the BCA concentration used. This indicates that the "auto-hydrolysis" of DCF-MA in PBS (k_{buffer}) could not be ignored for the determination of k_{cat}/K_M. Compared to the non-enzymatic hydrolysis of $DCFH_2$-DA, the non-enzymatic hydrolysis of DCF-MA is much faster. Since DCF-MA and $DCFH_2$-MA are neither commercially available nor can they be obtained in a stable form *in situ* (in contrast to $DCFH_2$-DA or $DCFH_2$, respectively), k_{buffer} for the hydrolysis of DCF-MA in PBS was determined from measurements with an enzyme reactor system through which a substrate solution containing $DCFH_2$-DA and H_2O_2 was pumped (at 5 μL min^{-1}). The outflow contained DCF-MA and DCF only (see **Figure 2**). The immobilized enzymes used (BCA and HRP) did not leak from the enzyme reactor (see the enzyme reactor washing step, Chapter 13). Following the time-dependent changes of the UV/vis absorption spectrum of the outflow kept inside a cuvette and placed into a spectrophotometer, k_{buffer} for the non-enzymatic hydrolysis of DCF-MA could be determined by fitting the exponential decay of [DCF-MA] (as obtained by following λ = 503 and 460 nm)[S11], yielding $k_{buffer} = 0.0056\ min^{-1}$. The contribution of this "auto-hydrolysis" could then be subtracted from the rate constant that was obtained by fitting of the experimental data with BCA added to DCF-DA in bulk solution. This led to similar catalytic efficiencies determined with the two BCA concentrations used (k_{cat}/K_M determined using 1 or 3 μM BCA = 0.0030 or 0.0036 $\mu M^{-1}\ min^{-1}$, respectively), confirming the assumed first-order kinetic model ($k = (k_{cat}/K_M) \cdot [BCA]_0 + k_{buffer}$).

Surprisingly, the calculated contribution to the hydrolysis rate of 1 μM BCA (0.0030 min^{-1}) was in the same region as k_{buffer} (PBS) = 0.0056 min^{-1}, indicating that DCF-MA is a bad substrate for BCA in PBS. Therefore, *Hyd_3* of the cascade reaction (see **Figure 3**) appears to occur also un-catalyzed in PBS, while it is accelerated in the presence of BCA.

While we were aware of this, the hydrolysis of DCF-MA was generally not relevant for the information we aimed to extract from the cascade assay. Essential was to monitor the HRP-catalyzed oxidation of $DCFH_2$-MA at the isosbestic point λ_{iso} (DCF-MA/DCF) = 460 nm for gaining information about the performance of *immobilized HRP* in the HRP reactors. For assessing the performance of *immobilized BCA* in the BCA reactors quantitatively, the rate of hydrolysis of $DCFH_2$-DA was taken as indicator.

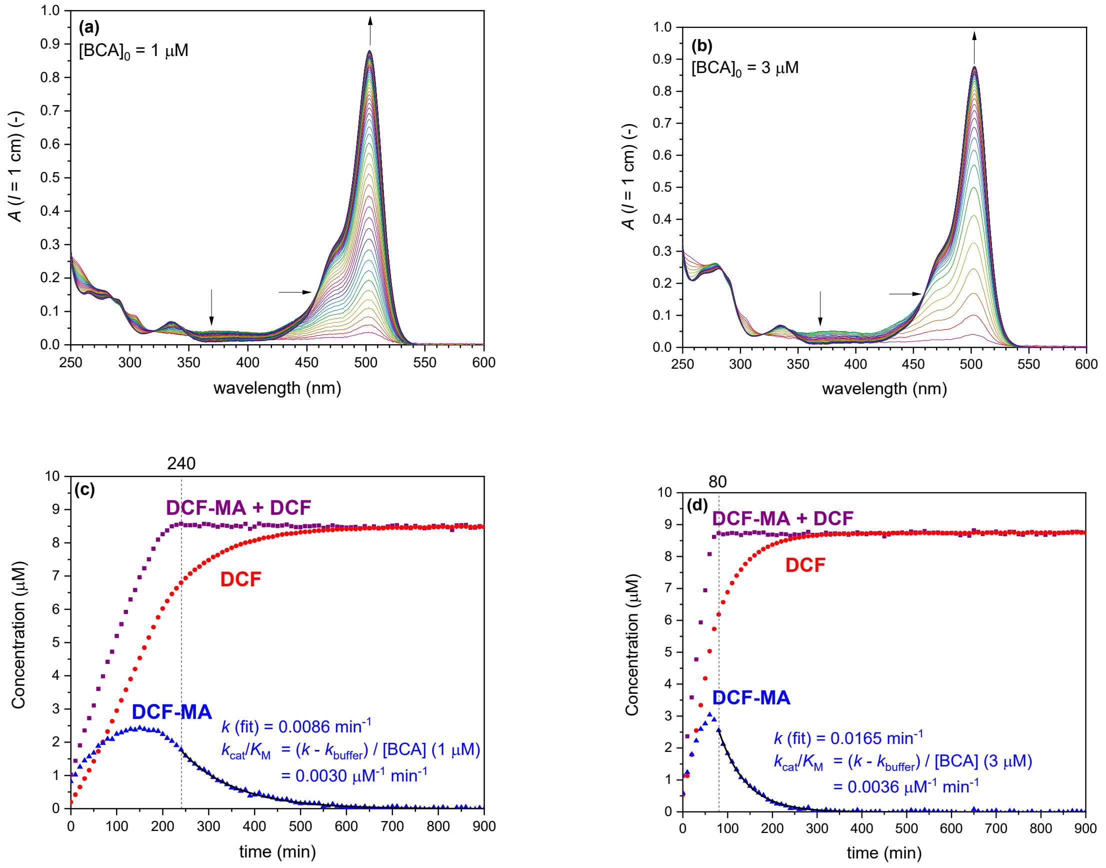

Figure S25. BCA-catalyzed hydrolysis of DCF-DA to DCF-MA and the follow-up hydrolysis of DCF-MA to DCF in bulk solution. BCA − 1 µM **(a)** and **(c)** or 3 µM **(b)** and **(d)** − and DCF-DA (10 µM, 1 vol% DMSO) were mixed in PBS (100 mM NaH_2PO_4, 150 mM NaCl, pH = 7.2) and the UV/vis absorption spectra were recorded every 5 min for 15 h at 25 °C.

(a) and **(b)**: The full spectra are shown. As soon as A_{460} became stable, all DCF-DA was used up since λ = 460 nm is the isosbestic point of DCF-MA and DCF (ε_{460} (DCF-MA/DCF, pH = 7.2) = 19 800 $M^{-1}cm^{-1}$).[S11] The decrease of A_{370} and the simultaneous increase of A_{503} indicated the hydrolysis of DCF-MA to DCF. For the reference spectra of the different components, see Ghéczy et al. (2020).[S11]

(c) and **(d)**: The change in the concentrations of DCF-MA and DCF during the curse of the reaction were calculated based on the molar absorptions at the characteristic wavelengths of these compounds and the isosbestic point mentioned above. The time needed for the rapid initial hydrolysis of DCF-DA (indicated by the formation of a constant total concentration of DCF-MA + DCF in purple) was inverse proportional to the BCA concentration used, 1 µM **(c)** or 3 µM **(d)**. Therefore, the contribution from the non-enzymatic "auto-hydrolysis" of DCF-DA to DCF-MA in PBS was negligible under these conditions. This was not the case anymore for the hydrolysis of DCF-MA to DCF, see text. The determined contribution from PBS was k_{buffer} = 0.0056 min^{-1}. Subtracting this PBS contribution from the experimental rate constants obtained in the presence of BCA, the catalytic efficiencies was calculated. For [BCA] = 1 µM, **(c)**: k_{cat}/K_M = 0.0030 μM^{-1} min^{-1}, R^2 = 0.9947, t_{fit} = 240 – 900 min; for [BCA] = 3 µM, **(d)**: k_{cat}/K_M = 0.0036 μM^{-1} min^{-1}, R^2 = 0.9953, t_{fit} = 80 – 900 min.

24. Determination of the HRP-Catalyzed Rate of Oxidation of $DCFH_2$-MA in Bulk Solution by Analyzing the Cascade Reaction Proceeding Along *Pathway 2*

For the BCA- and HRP-catalyzed cascade reaction system shown in **Figure 3**, with $DCFH_2$-DA and H_2O_2 as added substrates, the dependency of the extent of oxidation of $DCFH_2$-MA and $DCFH_2$ was determined by running a reaction in bulk solution and following the increase in absorbance at λ_{iso} (DCF-MA/DCF) = 460 nm, A_{460}, with time during the first 60 min of reaction (t_{obs} = 0 – 60 min). The starting reaction conditions were: $[DCFH_2\text{-DA}]_0$ = 50 µM, $[H_2O_2]_0$ = 0, 10 or 30 µM; [BCA] = 1.5 or 3.5 µM, [HRP] = 0, 25, 50, or 100 nM, see Experimental Section 2.3.3. For [BCA] = 1.5 µM and t_{obs} = 0 – 60 min, the rates of hydrolysis of $DCFH_2$-MA to $DCFH_2$ and DCF-MA to DCF were low and could be neglected. Therefore, HRP was mainly exposed to $DCFH_2$-MA as oxidizable substrate (which continuously formed *in situ* due to the BCA-catalyzed hydrolysis of $DCFH_2$-DA), see **Figure S24**. The cascade reaction thus proceeded predominantly along *pathway 2* (hardly any change in A_{460} originated from the oxidation of $DCFH_2$). Overall, for the conditions used, the initial oxidation rate during the cascade reaction was a measure for the HRP-activity in the presence of H_2O_2 as terminal oxidant towards $DCFH_2$-MA as reducing substrate.

As shown in **Figure S26a**, the oxidation rate was proportional to the used HRP concentration ([HRP] = 0, 25, 50 or 100 nM; using [BCA] = 1.5 µM and $[H_2O_2]$ = 30 µM). The thereby observed rate constants for native HRP in solution (k_{obs} = 3.5 and 4.5 s^{-1} for $[H_2O_2]_0$ = 10 and 30 µM, respectively) were used for comparison to the activity of HRP in denpol-BAH-HRP conjugates in bulk solution (see Chapter 7) or immobilized inside monolith pieces (see Chapter 20). Varying the initial H_2O_2 or BCA concentrations had only a marginal effect on the rate of oxidation, see **Figure**

S26b. Furthermore, the extent of "auto-oxidation" of DCFH2-MA, in the absence of either HRP or H_2O_2, was low (see black data in **Figure S26**).

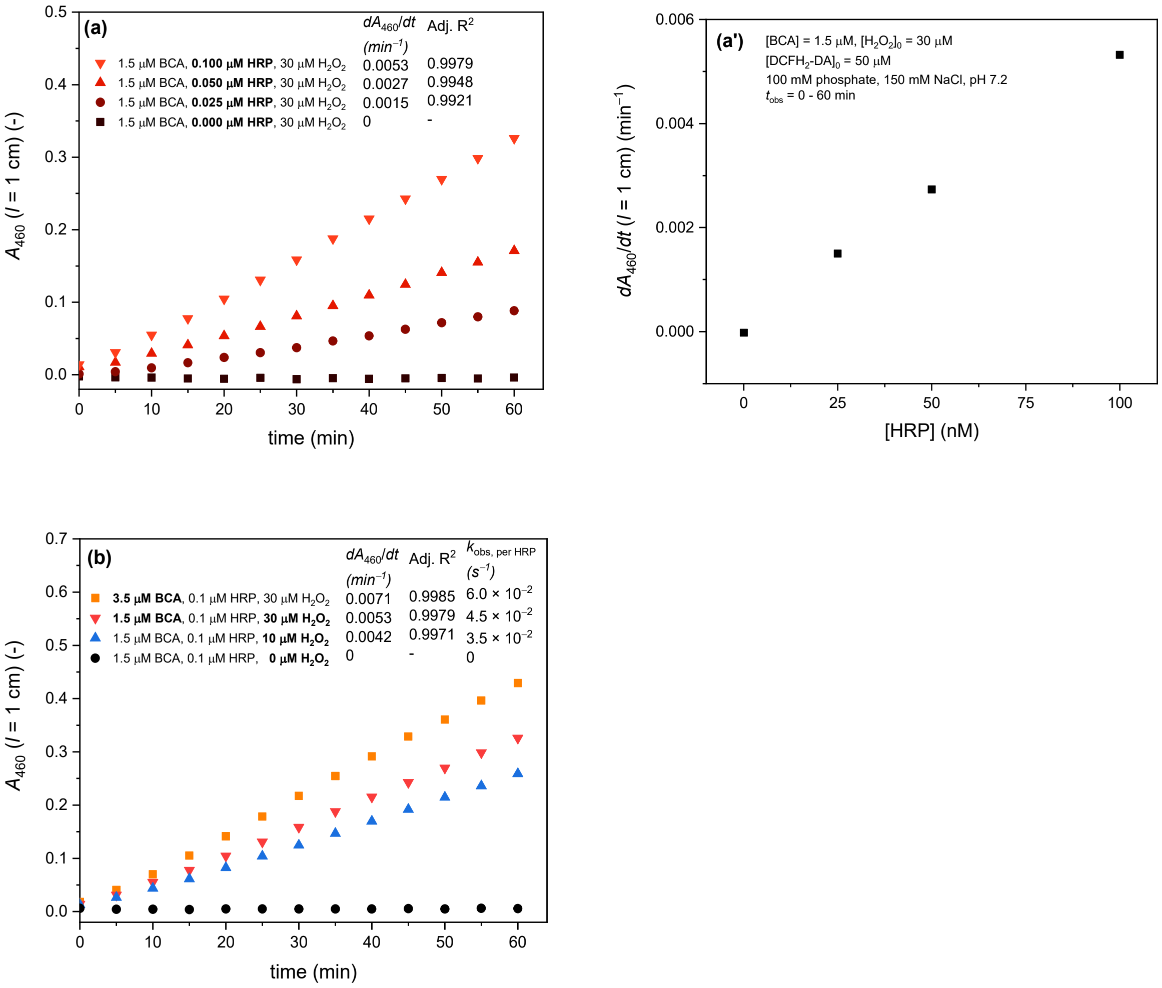

Figure S26. Change of A_{460} with reaction time for the cascade reaction run in bulk solution using [DCFH2-DA]$_0$ = 50 µM, H_2O_2 as well as native BCA and HRP as catalysts (PBS: 100 mM phosphate, 150 mM NaCl, pH = 7.2). The initial concentrations of HRP **(a, a')** or BCA and H_2O_2 **(b)** were varied and the change in A_{460} with time (dA_{460}/dt) was fitted for t_{obs} = 0 – 60 min. Given are the adjusted R^2 values of the fits and the observed rate constants (k_{obs}), describing DCF-MA + DCF formed per s and per HRP molecule (using the molar absorption of the reaction products at their isosbestic point, ε_{460} (DCF/DCF-MA, pH = 7.2) = 19 800 $M^{-1}cm^{-1}$, see Ghéczy et al. (2020)).[S11]

25. *In Situ* Formation of $DCFH_2$ and Kinetics of the HRP-Catalyzed Oxidation of $DCFH_2$

Preparation of $DCFH_2$ by hydrolysis of $DCFH_2$-DA with aqueous sodium hydroxide. For the following investigation of the HRP-catalyzed oxidation of $DCFH_2$, a $DCFH_2$ stock solution (500 µM in H_2O, 10 vol% DMSO) was prepared on the same day it was used by hydrolysis of $DCFH_2$-DA at alkaline conditions.[S11] For the preparation of 3 mL of a $DCFH_2$ stock solution, 60 µL of NaOH 2 M was added to 2.52 mL H_2O in a light-protected 5 mL PP tube and mixed. Then, 300 µL of $DCFH_2$-DA (5 mM in DMSO) was added, the tube was closed with a stopper and then repeatedly inverted for about one minute. After resting for 10 min (safely long enough for completed $DCFH_2$-DA hydrolysis), 120 µL HCl 1 M was added and the solution was mixed (to neutralize the added NaOH). Note that the stock solution finally also contained 40 mM NaCl. The UV/vis absorption spectrum of 2 µL of the obtained $DCFH_2$ stock solution was measured by using the NanoDrop spectrophotometer and compared to the spectrum of $DCFH_2$ obtained previously, see Fig. 2 in Ghéczy et al. (2020)[S11] and also Reiniers et al. (2017).[S15] The prepared aqueous $DCFH_2$ solution was stored at 4 °C and the UV/vis absorption spectrum was recorded every few hours. No significant changes of the spectrum were observed for at least 12 h of storage.

HRP-catalyzed oxidation of $DCFH_2$ to DCF in bulk solution with H_2O_2 as teminal oxidant. The HRP-catalyzed oxidation of $DCFH_2$ to DCF (ε_{503} (DCF, pH = 7.2) = 101 900 M^{-1} cm^{-1})[S11] with H_2O_2 as oxidizing substrate was investigated using native HRP in bulk solution inside a 1 mL PS cuvette (l = 1 cm) at RT. The concentrations used were [HRP] = 100 nM, $[DCFH_2]_0$ = 50 µM and $[H_2O_2]_0$ = 30 µM in PBS, pH = 7.2 (1 vol% DMSO). For this, 860 µL PBS, 10 µL HRP stock solution (10 µM in PBS) and 100 µL $DCFH_2$ stock solution (500 µM in H_2O, 10 vol% DMSO) were combined inside the cuvette and mixed. After quickly adding 30 µL H_2O_2 solution (1 mM in PBS, freshly prepared on the day used from a 2 M H_2O_2 stock solution in H_2O), the solution was

mixed and immediately afterwards the UV/vis absorption spectrum was measured at intervals of 5 s for 2 min. The initial increase in A_{503} with time was linearly fitted. For investigating the dependency of the initial reaction rate, dA_{503}/dt, on the initial concentrations of the reaction components, the concentration of only one component was varied at a time, while the others remained the same. For varying either the concentration of HRP (0 or 5 – 200 nM), $DCFH_2$ (0 or 1 – 100 μM), or H_2O_2 (0 or 0.2 – 30.0 μM) in the cuvette, the same stock solutions were used. The volumes of the added stock solutions were adjusted accordingly while balancing the total volume to 1 mL with PBS. With this procedure, the amount of DMSO in the cuvette varied between 0 and 2.0 vol%.

Figure S27 shows a linear dependence of the initial rate of DCF formation from $DCFH_2$, dA_{503}/dt, on the concentration of HRP. The thereby observed rate constant was $k_{obs} = 3.0 \times 10^{-1}$ s^{-1} ($[DCFH_2]_0$ = 50 μM and $[H_2O_2]$ = 30 μM).

For the reaction rate dependency on the concentration of $DCFH_2$, see **Figure S28a**. Assuming Michaelis-Menten kinetics, the Michaelis contstant determined by fitting was K_M ($DCFH_2$) = 9.5 μM (at $[H_2O_2]_0$ = 30 μM and [HRP] = 100 nM).

Figure S28b shows a saturation of the reaction rate with increasing initial H_2O_2 concentration at $[H_2O_2]_0 \approx 1$ μM (at $[DCFH_2]_0$ = 50 μM and [HRP] = 100 nM). Although in this case a fit of the experimental data to the Michaelis-Menten equation was not possible (adjusted R^2 = 0.90487 only), the data show that half the maximal initial rate of DCF formation was reached at $[H_2O_2]_0 \approx$ 0.2 μM. The line drawn in **Figure S28b** is for guiding the eye to show the saturation behavior.

Control experiments are shown in **Figure S29**. *DCF did not form* from $DCFH_2$ under the conditions used *without added HRP*. However, some *DCF-formation occurred* from $DCFH_2$ in the presence of HRP *even in the absence of* H_2O_2. This HRP-mediated oxidation without added H_2O_2

was found to be significant when the reaction mixtures were exposed to light, see Chapter 26, resulting in a higher H_2O_2 consumption for the formation of oxidation products than stoichiometrically possible on the basis of the added amounts of H_2O_2 (likely involvement of photochemical reactions on $DCFH_2$ in the presence of HRP).

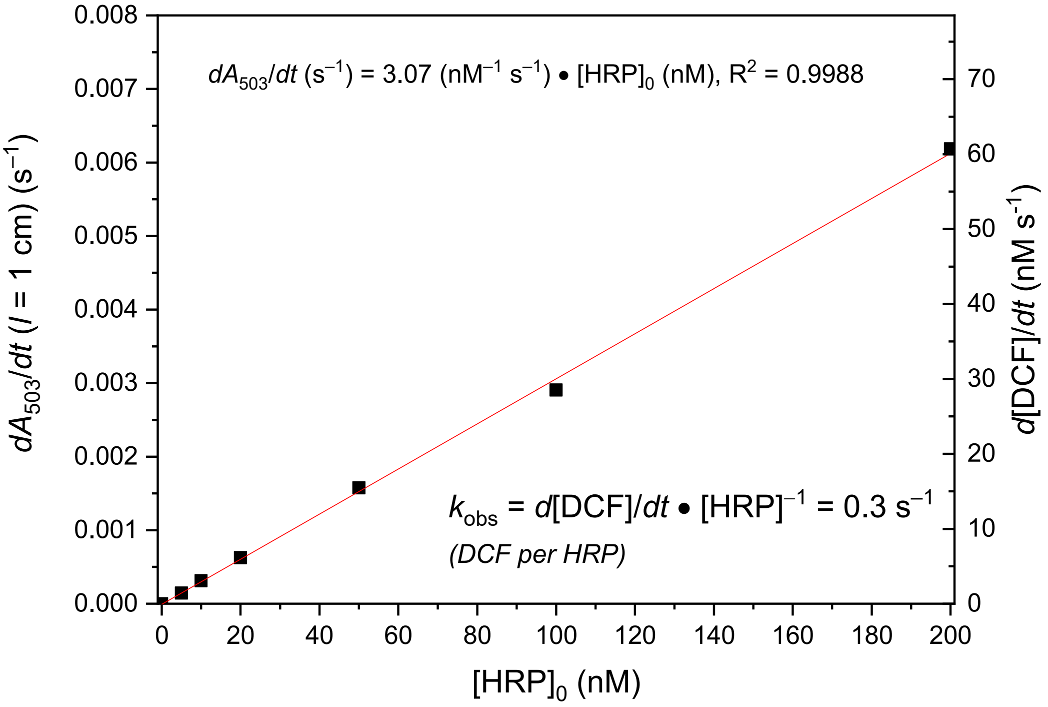

Figure S27. Oxidation of $DCFH_2$ in bulk solution at pH = 7.2 and RT, catalyzed by HRP in the presence of H_2O_2 as terminal oxidant. The initial rate of DCF formation, d[DCF]/dt (vertical axis on the right-hand side), is plotted as a function of the native HRP concentration; ε_{503} (DCF, pH = 7.2) = 101 900 $M^{-1}cm^{-1}$, see Ghéczy et al. (2020).[S11] $[DCFH_2]_0$ = 50 µM, $[H_2O_2]_0$ = 30 µM, [HRP] = 0, 5 – 200 nM, PBS (100 mM phosphate, 150 mM NaCl, pH = 7.2);. Linear dependence of the rate of DCF formation on [HRP], yielding an observed rate constant of $k_{obs} = d[DCF]/dt\ [HRP]^{-1} = 0.3\ s^{-1}$.

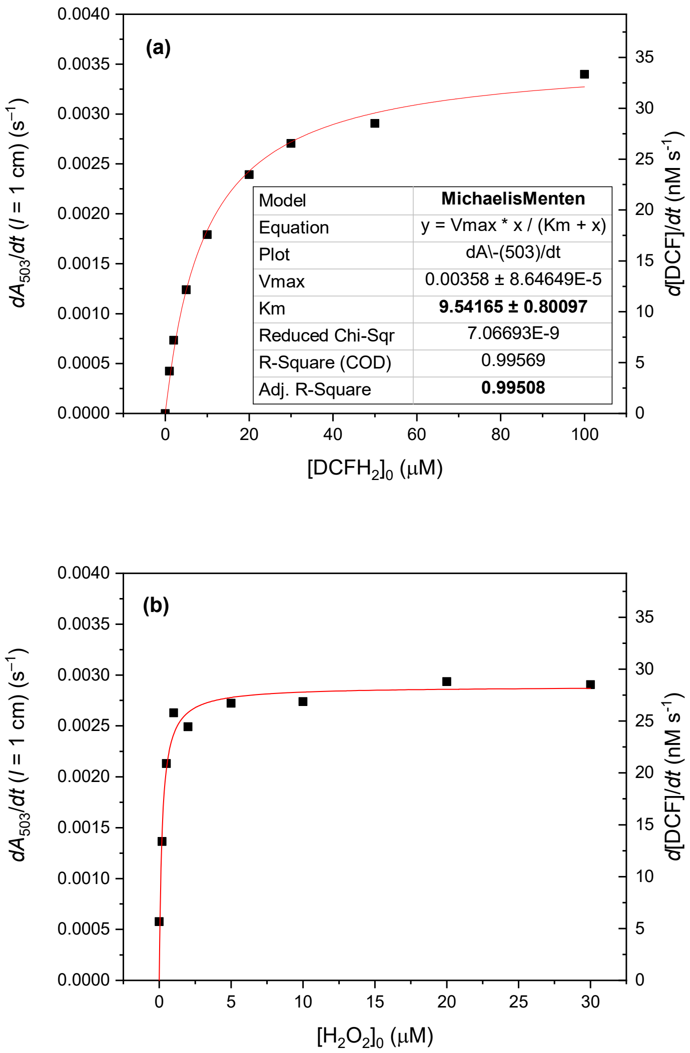

Figure S28. Oxidation of $DCFH_2$ in bulk solution, catalyzed by HRP in the presence of H_2O_2 as terminal oxidant. PBS (100 mM phosphate, 150 mM NaCl, pH = 7.2), RT; ε_{503} (DCF, pH = 7.2) = 101 900 $M^{-1}cm^{-1}$, see Ghéczy et al. (2020).[S11]

(a): The initial rate of DCF formation, *d*[DCF]/*dt* (vertical axis on the right hand side), is plotted as a function of the $DCFH_2$ concentration. [HRP] = 100 nM, $[H_2O_2]_0$ = 30 µM, [$DCFH_2$] = 0, 1 – 100 µM. The experimental data points were fitted with the Michaelis-Menten equation, yielding $K_M(DCFH_2)$ = 9.5 µM.

(b): The initial rate of DCF formation, *d*[DCF]/*dt* (vertical axis on the right hand side), is plotted as a function of the H_2O_2 concentration. [HRP] = 100 nM, [$DCFH_2$] = 50 µM, $[H_2O_2]_0$ = 0, 0.2 – 30 µM. Note that the initial reaction rate without added H_2O_2 was not negligible. For more details about the initial reaction rate observed at $[H_2O_2]_0$ = 0 µM, see **Figure S29**. The red line is for guiding the eye.

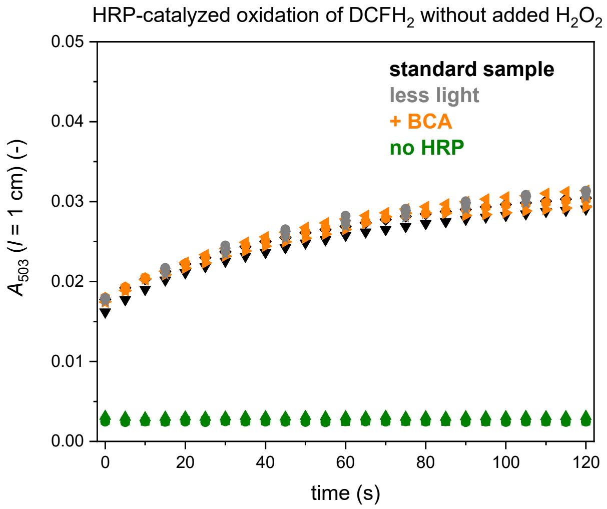

Figure S29. HRP-catalyzed oxidation of $DCFH_2$ in PBS (100 mM phosphate, 150 mM NaCl, pH = 7.2), RT, *without added* H_2O_2, using $[HRP]_0$ = 100 nM and $[DCFH_2]_0$ = 50 µM. The only light source present was the beam of the spectrometer (spectrum recorded every 5 s). Reducing the light beam frequency (to 15 s) and adding BCA (1.5 µM) did not change the formation rate of DCF (indicated by the change in A_{503}, ε_{503} (DCF, pH = 7.2) = 101 900 $M^{-1}cm^{-1}$, see Ghéczy et al. (2020)).[S11] No reaction was observed without HRP (green data points). All measurements in triplicates.

26. Effect of Light on the Quantification of H_2O_2 with the Cascade Reaction

Based on the observation that the HRP-catalyzed oxidation of $DCFH_2$ occurs without added H_2O_2 (see Chapter 25), the influence of light on the cascade reaction was investigated in bulk solution. This was done by using cascade reaction conditions for which the reaction proceeded *via pathway 2* (see **Figure 3**) and for which H_2O_2 was limiting, with a clear dependence of the value of A_{460} obtained once the added H_2O_2 was consumed. A_{460} is the absorption at λ_{iso} (DCF-MA/DCF) = 460 nm and is proportional to the concentration of DCF-MA + DCF.

The cascade reaction was carried out in PBS (100 mM phosphate, 150 mM NaCl, pH = 7.2) at RT either inside dark, light-protected PP tubes or inside transparent PP tubes. The reactions were carried out at total volumes of 1 mL as follows (see Experimental Section 2.3.3.): To a PBS solution containing native BCA (1.5 μM) and native HRP (100 nM), 10 μL of a 5 mM $DCFH_2$-DA (in DMSO; $[DCFH_2\text{-DA}]_0$ = 50 μM) and finally a small volume of a 100 μM H_2O_2 solution (freshly prepared from a 2 M H_2O_2 stock in water) were added ($[H_2O_2]_0$ = 0, 1 – 10 μM) in quick succession. H_2O_2 was added from a 100 μM stock solution (freshly prepared from a 2M stock in water). The tubes were closed and mixed. A_{460} was measured after 2 and 4 h of incubation at RT. Under these conditions, HRP was mainly exposed to $DCFH_2$-MA as oxidizable substrate (*pathway 2*) and for $[BCA]_0$ = 1.5 μM, H_2O_2 was completely consumed after about 90 min for $[H_2O_2]_0$ = 10 μM (see **Figure 8B**). There was hardly any contribution to A_{460} from the oxidation of $DCFH_2$.

The results obtained are shown in **Figure S30**. **Figure S30a** is the same as **Figure 8B**. It shows the situation for the cascade reaction run *in light-protected PP reaction tubes*, analyzed after 2 h. In **Figure S30b**, the same data points for measurements in light-protected tubes after 2 h are also shown (filled black symbols), together with data points of measurements after 4 h (empty black

data points). The orange data points in **Figure S30b** refer to measurements of reaction mixtures that were run *in transparent, light-exposed PP reaction tubes*.

The difference between these two types of measurements is clear. For the reaction run *in light-protected PP reaction tubes*, there is a linear dependency of A_{460}, *i.e.*, [DCF-MA] + [DCF], on $[H_2O_2]_0$, and the measured amounts of consumed H_2O_2 correlates with the expected, maximal amount of oxidized $DCFH_2$-MA. For each equivalent H_2O_2, two equivalents reducing substrates are oxidized (according to the peroxidase mechanism of HRP).[S2] For the reaction run *in transparent, light-exposed PP reaction tubes*, considerably more $DCFH_2$-MA was oxidizd than expected. Therefore, in the presence of HRP and H_2O_2, a photochemical reaction must have taken place which led to an additional oxidation of $DCFH_2$-MA, through a mechanism which is different from the peroxidase mechanism.

For the oxidation of $DCFH_2$, the effect of light exposure was already shown and discussed, see Chapter 25, **Figure S29**. In that case, however, the oxidation of $DCFH_2$ *occurred in the presence of HRP without added* H_2O_2 (**Figure S29**) In contrast, for the oxidation of $DCFH_2$-MA *in the presence of HRP, light did not have an effect without added* H_2O_2 (**Figure S30b**). Further experiments were carried out with $DCFH_2$ as substrate, as summarized in the following.

A PBS solution containing 50 μM $DCFH_2$, 100 nM HRP and 3 μM H_2O_2 was kept either inside black, light-protected PP reaction tubes or inside transparent, light-exposed PP reaction tubes for up to 4 h. The amount of DCF produced in both cases was higher than the expected 6 μM, even for the light-protected samples, see **Figure S31**. For the light-exposed reaction mixture, however, the amount of DCF formed was much higher than for the light-protected reaction mixture. As a consequence of these observations, all further experiments involving the cascade reaction were

carried out light-protected. Please note, that the samples had to be exposed to light temporarily for measuring of the UV/vis absorption spectrum of the reaction mixtures.

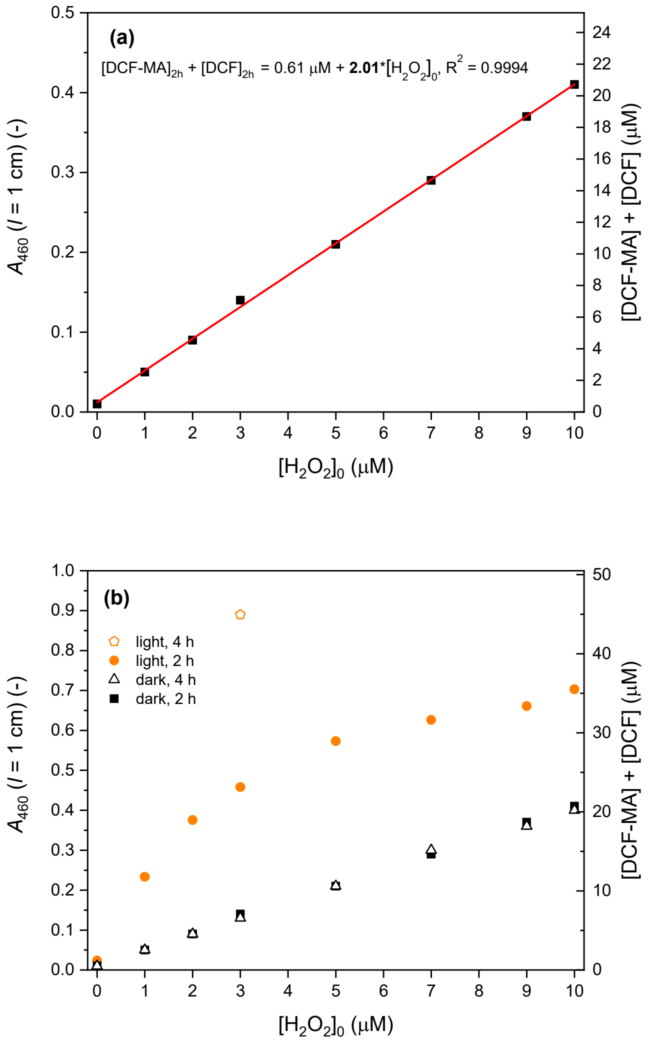

Figure S30. Cascade reaction in bulk solution with $[DCFH_2\text{-}DA]_0$ = 50 µM, [BCA] = 1.5 µM, [HRP] = 100 nM, and $[H_2O_2]_0$ = 0, 1 – 10 µM in PBS (100 mM phosphate, 150 mM NaCl, pH = 7.2).
(a = Figure 8D) The reaction was run inside *light-protected* PP reaction tubes for 2 h and A_{460} was measured and calculated to [DCF-MA] + [DCF] (using ε_{460} (DCF-MA/DCF) = 19 800 $M^{-1}cm^{-1}$).[S11] The red line represents a linear fit of $[DCF\text{-}MA]_{2h} + [DCF]_{2h}$ against $[H_2O_2]_0$.
(b) A_{460} of the *light-protected* samples shown in (a) are the filled black data points in (b). The samples were remeasured after 4 h (empty black data points), without showing a significant change. The same experiment was repeated inside *light-exposed* PP reaction tubes (orange data points).

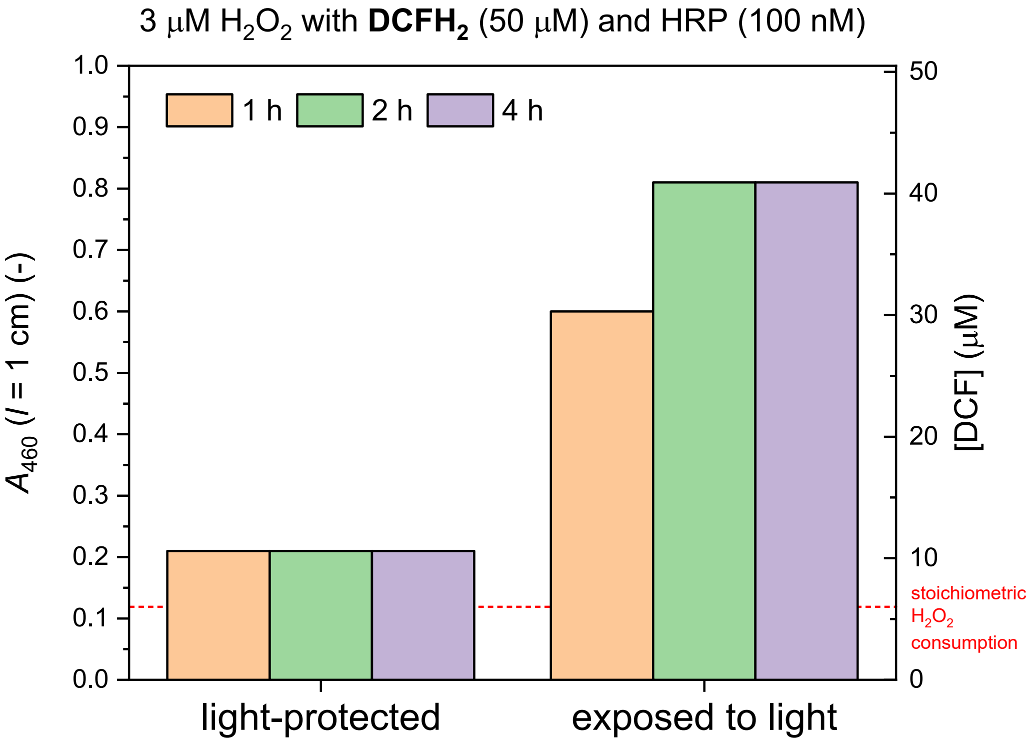

Figure S31. Comparison of the effect of light exposure on the HRP-catalyzed oxidation of $DCFH_2$ in bulk solution. $[DCFH_2]_0$ = 50 µM, [HRP] = 100 nM, $[H_2O_2]_0$ = 3 µM, in PBS bulk solution (100 mM phosphate, 150 mM NaCl, pH = 7.2). The reaction was run either light-protected or light-exposed for up to 4 h and A_{460} was measured and converted to [DCF] (using ε_{460} (DCF-MA/DCF) = 19 800 $M^{-1}cm^{-1}$).[S11] When repeating the same experiments with BCA present in addition ([BCA] = 1.5 µM), the very same values resulted. The red dashed line indicates the stoichiometric production of DCF as expected considering the added amount of H_2O_2 (2 equivalents DCF produced for 1 equivalent H_2O_2 consumed)[S2] if H_2O_2 is not reformed in the reaction mixture. The used $DCFH_2$ stock solution was checked after 4 h to not contain any DCF. For the initial oxidation rate of $DCFH_2$ without any added H_2O_2, see **Figure S29**.

27. Comparison of the HRP/H_2O_2-Catalyzed Oxidation of either $DCFH_2$-MA (Intermediate of the Cascade Reaction with BCA and $DCFH_2$-DA) or $DCFH_2$

The data shown and discussed in Chapters 24 - 26 on the HRP/H_2O_2-catalyzed oxidation in bulk solution of $DCFH_2$-MA (formed as intermediate of the cascde reaction) or $DCFH_2$ allow to draw conclusions that are summarized in the following.

Observed reaction rate constants. Both substrates allowed the determination of initial oxidation rates that were proportional to the amount of active HRP. With $[H_2O_2]_0$ = 30 μM (and similar $DCFH_2$-MA and $DCFH_2$ concentrations), the oxidation of $DCFH_2$ was about one order of magnitude faster than the one of $DCFH_2$-MA ($k_{obs} = 0.3\ s^{-1}$ *vs.* $k_{obs} = 0.045\ s^{-1}$), in agreement with our previous findings, see Chapter 15 of the Electronic Supporting Information of Ghéczy et al. (2020).[S11]

H_2O_2 consumption during the reactions. Both substrates, $DCFH_2$-MA and $DCFH_2$, showed no significant oxidation without the presence of HRP. When protected from light, $DCFH_2$-MA showed no significant "auto-oxidation" without added H_2O_2. $DCFH_2$ on the other hand showed some oxidation even without light-exposure or without added H_2O_2. Consequently, a stoichiometric H_2O_2 consumption was only seen for the light-protected oxidation of $DCFH_2$-MA (as part of the cascade reaction proceeding *via pathway 2*, see **Figure 3**). When the reactions were exposed to light, the "auto-oxidation" became either stronger (in the case of $DCFH_2$) or it became relevant at all (for $DCFH_2$-MA). Interestingly, this additional oxidation beyond stoichiometric H_2O_2 consumption became much stronger if H_2O_2 was added (at 1 μM at the conditions used), but did not linearly increase if more H_2O_2 was added (up to 10 μM tested), see **Figure 30b**. This strongly suggested a H_2O_2 reformation mechanism as mentioned in our previous work on the basis of literature data by other research groups, see Scheme 1 in Ghéczy et al. (2020).[S11] If the "auto-

oxidation" stems from a postulated H_2O_2-reforming mechanism, this would mean that the formation of H_2O_2 from $O_2^{\bullet-}$ is light-dependent.

28. Analysis of the Cascade Reaction Outflow from Two Types of Reactor Systems − Consisting of either Sequentially or Co-immobilized BCA and HRP − Through Which a Solution of $DCFH_2$-DA and H_2O_2 as Substrates was Pumped

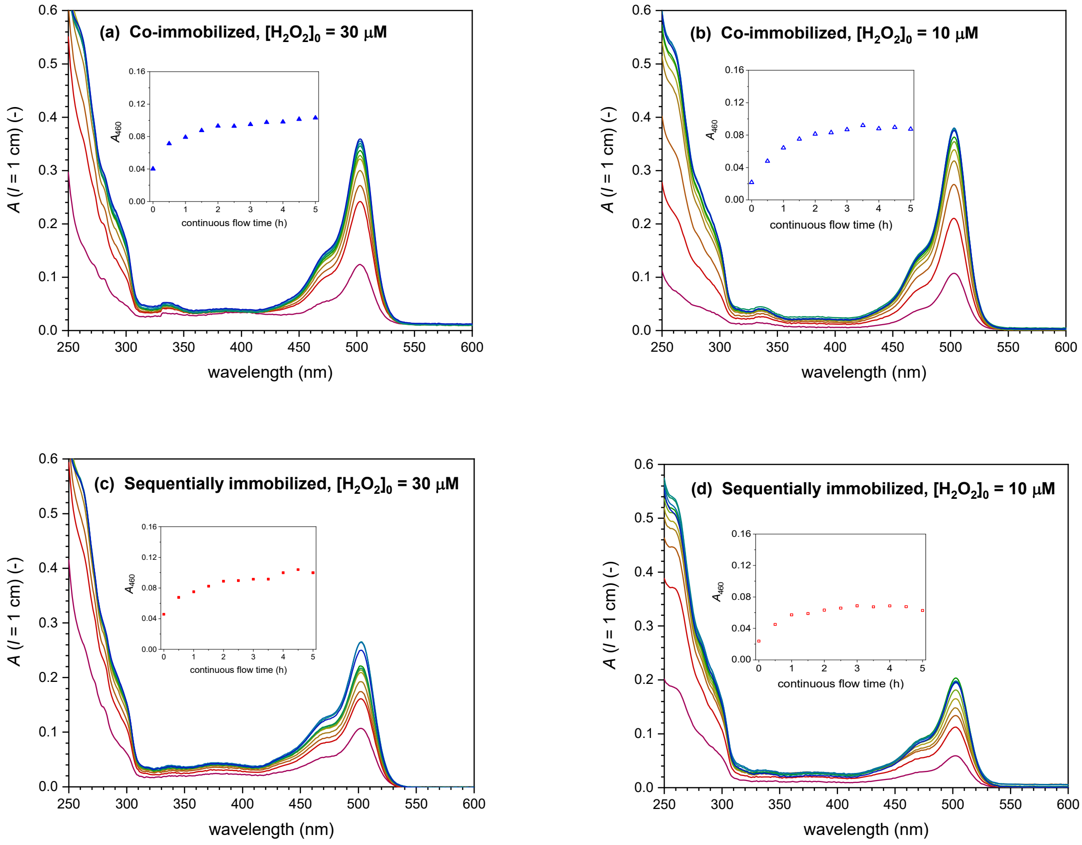

Figure S32. UV/vis absorption spectra of the regularly pooled outflow from two types of enzyme reactor systems - co-immobilized (**a** + **b**) or sequentially immobilized BCA and HRP (**c** + **d**) − through which a substrate solution consisting of $DCFH_2$-DA (50 µM, 1 vol% DMSO) and H_2O_2 (30 µM (**a** + **c**) or 10 µM (**b** + **d**)) in PBS (100 mM NaH_2PO_4, 150 mM NaCl, pH = 7.2) were pumped for 5 h at a flow rate of 5 µL min^{-1} at RT. The outflow was pooled every 30 min (see 2.8.4. for details). A_{460}, related to the total concentration of [DCF-MA] + [DCF] (ε_{460} (DCF-MA/DCF, pH = 7.2) = 19 800 $M^{-1}cm^{-1}$),[S11] is shown in the insets; A_{460} stabilizes after about 4 h of continuous flow. A comparison of A_{460} in the steady-state outflow between co-immobilization and sequentially immobilized enzymes is shown in **Figure 9**.
For both enzyme reactor systems, the same amounts of the respective enzymes were either co-immobilized (**a** + **b**) or sequentially immobilized (**c** + **d**); both in two monolith pieces of identical diameter and length (l_m = 5 mm, $d_m \approx$ 4 mm, see reactor types ***c*** and ***d*** in **Figure 2**). For the preparation of the enzyme reactors, 50 µL solutions with defined concentrations of *de*-$PG2_{1000}$-BAH-BCA_{89} and/or *de*-$PG2_{1000}$-BAH-HRP_{40} were ued, see Experimental Section (2.6.3. and 2.6.4.).

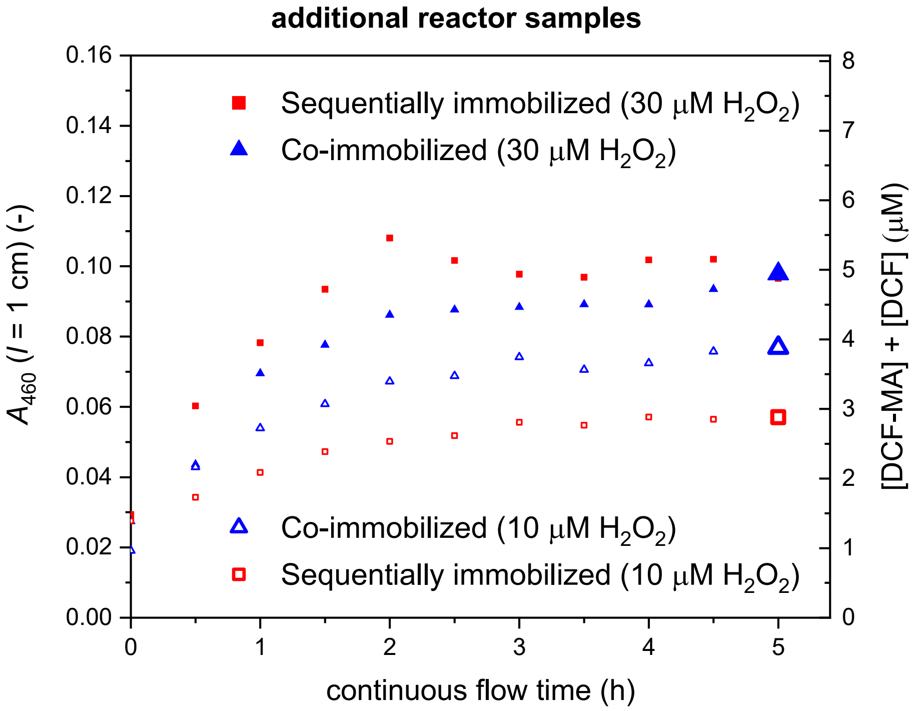

Figure S33. A_{460} was monitored in the outflow from the two types of enzyme reactor systems mentioned in **Figure S32**. The experiments were of the same type but the enzyme reactors were newly prepared to evaluate the reproducibility; the same results were obtained as shown in **Figure S32**. The substrate solution that was pumped though the two enzyme reactor systems consisted of $DCFH_2$-DA (50 µM, 1 vol% DMSO) and H_2O_2 (30 µM (filled symbols) or 10 µM (empty symbols)) in PBS (100 mM NaH_2PO_4, 150 mM NaCl, pH = 7.2); flow rate: 5 µL min^{-1}, RT, see leged of **Figure S32**. For the calculation of [DCF-MA] + [DCF], ε_{460} (DCF-MA/DCF, pH = 7.2) = 19 800 $M^{-1}cm^{-1}$)[S11] was taken into account.

29. Effect of Varying the Enzyme Reactor Length on the Outcome of the Cascade Reaction with $DCFH_2$-DA and H_2O_2 as Substrates Using Enzyme Reactor Systems with Sequentially Immobilized BCA and HRP

Different sequentially connected enzyme reactor systems were prepared, whereby the length of one of the two monolith pieces was varied – and thus the residence time (τ) (see 2.6.3 for reactor preparation). The performance of the enzyme reactors system was determined as described in Section 2.8.4. The substrate solution that was pumped through the enzyme reactor systems at a flow rate of 5 μL min^{-1} had the following composition: $[DCFH_2\text{-DA}]_0$ = 50 μM and $[H_2O_2]_0$ = 10 or 30 μM in PBS (100 mM NaH_2PO_4, 150 mM NaCl, pH = 7.2). See **Figure 10** (and sequential immobilization in **Figure 9**) for the type of enyzme reactor systems used and the averaged amounts of formed DCF-MA + DCF for the chosen conditions under steady-state. **Figure S34** shows the actual outflow data before the steady-state was reached and at steady-state (for all reactor systems). Varying the respective length of the monolith piece did only change the respective time the substrate solution was in contact with the immobilized enzymes, as the flow rate was not changed.

$[H_2O_2]_0$ = 30 μM. When prolonging the HRP-exposure time inside the second enzyme reactor, the product formation increased proportionally. For $[H_2O_2]_0$ = 30 μM and the enzyme reactors used, HRP was rate limiting. Prolonging the preceding BCA reactor did not increase the rate of oxidation of $DCFH_2$-MA (although the longer BCA reactor provided a higher concentration of oxidizable intermediate $DCFH_2$-MA). The immobilized HRP was apparently already saturated with intermediate. Compare **Figure 9** and **Figure 10**, and/or see **Figure S34**.

$[H_2O_2]_0$ = 10 μM. For $[H_2O_2]_0$ = 10 μM, a prolonged HRP-exposure time did not increase the amount of product formed, all H_2O_2 molecules might be already consumed before leaving the

enzyme reactor system. See Chapter 30, **Table S6**, for the outflow composition at steady state for the different enzyme reactor systems.

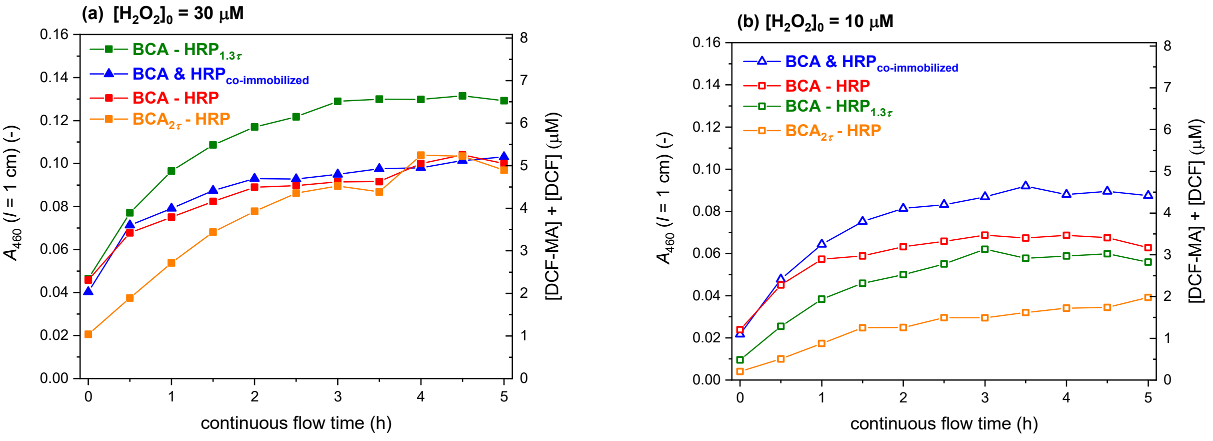

Figure S34. A_{460} monitored in the outflow from different enzyme reactor systems through which a substrate solution of $DCFH_2$-DA (50 µM, 1 vol% DMSO) and H_2O_2 (30 µM **(a)** or 10 µM **(b)**) in PBS (100 mM NaH_2PO_4, 150 mM NaCl, pH = 7.2) was pumped for a duration of 5 h at a flow rate of 5 µL min^{-1} at RT. A_{460} was converted to the total concentration of [DCF-MA] + [DCF] by using ε_{460} (DCF/DCF-MA, pH 7.2) = 19 800 $M^{-1}cm^{-1}$, see Ghéczy et al. (2020).[S11] A_{460} stabilized after about 4 h of continuous flow. See **Figure S32** for the changes of the entire UV/vis spectra of the pooled outflows. The four enzyme reactor systems used are indicated, see also **Figure 2**, enzyme reactor systems **(c)** and **(d)**.
For the preparation of the enzyme reactors from 50 µL solutions with defined concentrations of *de*-$PG2_{1000}$-BAH-BCA_{89} and/or *de*-$PG2_{1000}$-BAH-HRP_{40}, see Experimental Sections 2.6.3 and 2.6.4. In the case of the sequential setups, the enzyme-respective residence time (τ) was varied by varying the respective length of the monolith piece, using an accordingly higher volume of equally concentrated conjugate incubation solution (orange for BCA and green for HRP). The same amounts of the respective enzymes were either co-immobilized (blue) or sequentially immobilized (red) when using the standard length of monolith piece.

(a) At $[H_2O_2]_0$ = 30 µM, the product formation was *rate-limited by the HRP*-exposure (HRP concentration and residence time). Thus, all reactors except the one with a longer HRP-reactor showed the same product formation.
(b) At $[H_2O_2]_0$ = 10 µM, the product formation was *limited by* H_2O_2 (at least for the sequential setups). Therefore, the longer HRP-exposure did not lead to a higher product formation.

30. Determination of the Steady-State Composition in the Outflows from Different Enzyme-Reactor Systems Through Which a Substrate Solution Consisting of $DCFH_2$-DA and H_2O_2 were Pumped

The concentrations of remaining $DCFH_2$-DA (50 µM initially present) and of formed $DCFH_2$-MA, $DCFH_2$, DCF-MA, and DCF (see **Figure 3**) in the outflows at steady state from the different enzyme reactor systems shown in **Figure S34b** are given in **Table S6**, as obtained by spectral fitting (**Figure S35**), following the previously described procedure, see Ghéczy et al. (2020).[S11]

Table S6. Concentrations calculated by spectral fitting of the outflows after 5 h at a flow rate of 5 µL min^{-1} for the four different enzyme reactor systems shown in **Figure S34b**. The substrate solution that was pumped though the enzyme reactor systems had the follwing composition: $[DCFH_2\text{-DA}]_0$ = 50 µM, $[H_2O_2]_0$ = 10 µM, PBS (100 mM NaH_2PO_4, 150 mM NaCl, pH = 7.2). See **Figure S35** for the measured UV/vis absorption spectra in the pooled outflow that were fitted.

Enzyme Reactor System	$DCFH_2$ -DA (µM)	$DCFH_2$ -MA (µM)	$DCFH_2$ (µM)	DCF-MA (µM)	DCF (µM)
BCA & $HRP_{co\text{-}immobilized}$	28.1	16.8	-	0.7	3.6
BCA - HRP	28.5	15.5	1.9	1.2	1.9
BCA - $HRP_{1.3\tau}$[a]	28.0	14.2	2.0	1.1	1.7
$BCA_{2\tau}$ - HRP[a]	17.8	18.0	8.3	1.1	0.9

[a] Longer enzyme-respective residence time for longer monolith pieces in the enzyme reactor systems (the same enzyme concentration in the conjugate incubation solution was used).

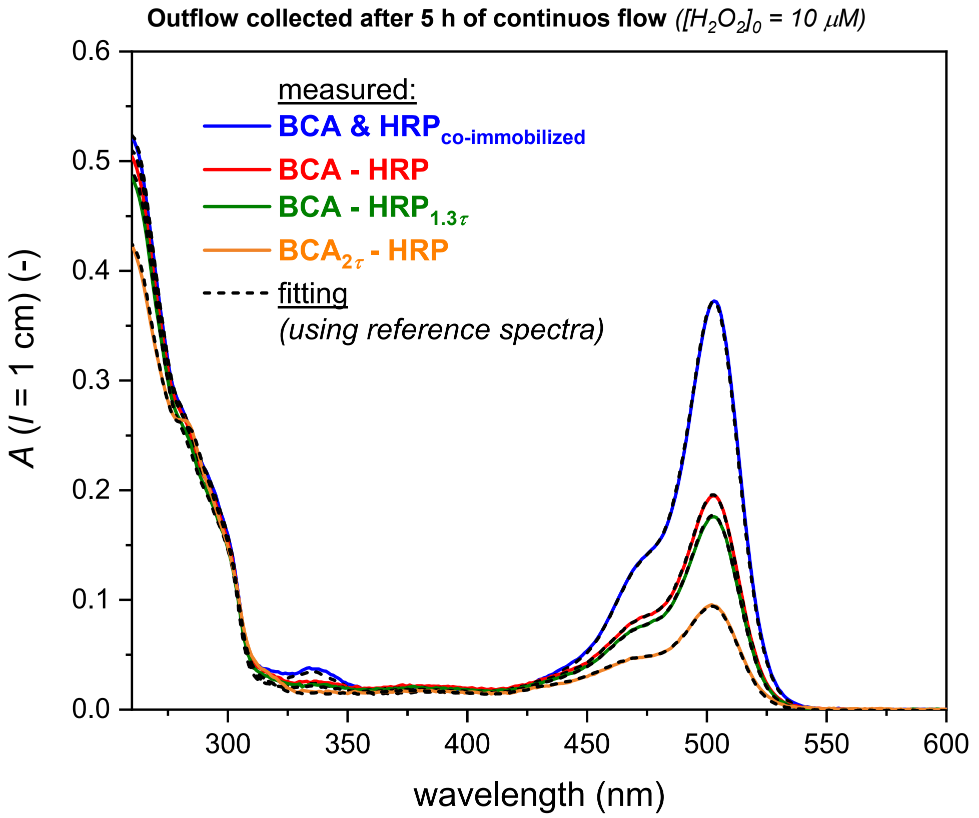

Figure S35. UV/vis absorption spectra of pooled outflows from the four different enzyme reactor systems shown in **Figure S34b** through which a substrate solution consisting of DCFH$_2$-DA (50 µM, 1 vol% DMSO) and H_2O_2 (10 µM) in PBS (100 mM NaH_2PO_4, 150 mM NaCl, pH = 7.2) were pumped at a flow rate of 5 µL min^{-1} at RT. The spectra were recorded of outflows pooled after 5h (steady-state conditions). Using reference spectra and molar absorptions at isosbestic points at pH = 7.2 determined in our previous work − Fig. 2 and Table 1 in Ghéczy et al. (2020)[S11] − the measured spectra in the outflow were fitted (dotted black curves). The concentrations used for the fitting indicated the composition of the outflow (see **Table S6**). For the preparation of the enzyme reactors from 50 µL solutions with defined concentrations of *de*-PG2$_{1000}$-BAH-BCA$_{89}$ and/or *de*-PG2$_{1000}$-BAH-HRP$_{40,}$ see Experimental Sections 2.6.3 and 2.6.4. In the case of the sequential setups, the enzyme-respective residence time (τ) was varied by varying the respective length of the monolith piece, using an accordingly higher volume of equally concentrated conjugate incubation solution (orange for BCA and green for HRP). The same amounts of the respective enzymes were either co-immobilized (blue) or sequentially immobilized (red) when using the standard length of the monolith piece.

31. Stability of H_2O_2 in Bulk Solution in the Presence of BCA and in Enzymatic Flow-Through Reactors Containing either *de*-PG2$_{1000}$-BAH-BCA$_{89}$ or *de*-PG2$_{1000}$-BAH-HRP$_{20}$

We investigated the stability of 10 µM H_2O_2 in PBS after passage through monolith pieces containing either immobilized *de*-PG2$_{1000}$-BAH-BCA$_{89}$ or immobilized *de*-PG2$_{1000}$-BAH-HRP$_{20}$ under similar conditions as used in the experiments in which a substrate solution containing DCFH$_2$-DA (50 µM) and H_2O_2 (10 µM) was passed at a flow rate of 5 µL min^{-1} through the different enzyme reactors. The concentration of H_2O_2 was determined before and after passing the H_2O_2 solution through the enzyme reactors by adding ABTS^{2-} and HRP, see **Figure S36**.

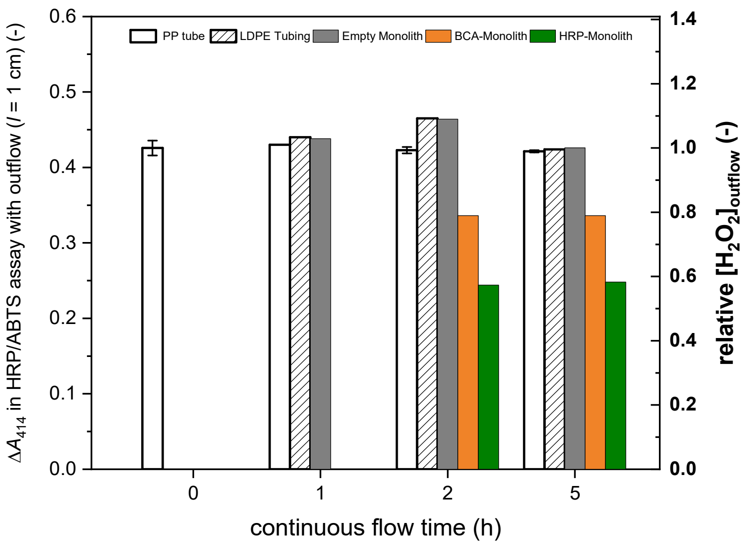

Figure S36. Stability of a 10 µM H_2O_2 solution in PBS (100 mM NaH_2PO_4, 150 mM NaCl, pH = 7.2), flowing through different enzyme reactors (at 5 µL min^{-1}, τ = 10 min). The different systems used were either LDPE tubing only, an "empty" monolith piece (placed within the LDPE tubing), or monolithic enzyme reactors containing immobilized enzymes.
For the preparation of the BCA reactor (orange bars), *de*-PG2$_{1000}$-BAH-BCA$_{89}$ was used at [BCA] = 5.13 µM in the conjugate incubation solution; for the preparation of the HRP-reactor (green bars), *de*-PG2$_{1000}$-BAH-HRP$_{20}$ was used at [HRP] = 0.5 µM in the conjugate incubation solution.
From the outflow, fractions were pooled every hour. An excess of native HRP (0.5 µM) and of the substrate ABTS^{2-} (1 mM) were added and the quickly stabilizing absorbance at λ = 414 nm, A_{414} (t ≈ 10 s) was taken as a relative measure for the amount of H_2O_2 present in the outflow collected after 1, 2 or 5 h. As a reference (100 % value, white bar), a part of the 10 µM H_2O_2 solution was kept in a light-protected PP tube and assayed every hour in the same way. This solution remained stable over time. In addition, a flow through the LDPE tubing as well as through the "empty" monolith piece did not result in a decrease of [H_2O_2] in the outflow. The decrease in H_2O_2 was thus originating from the immobilized conjugates (see text).

While H_2O_2 remained stable when passed through a monolith piece which did not contain any immobilized enzyme ("empty monolith"), in the case of the BCA reactor the concentration of H_2O_2 in the outflow decreased to 80% of the initial value, and in the case of the HRP reactor to 60%.

For immobilized *de*-$PG2_{1000}$-BAH-HRP_{20}, the observed consumption of H_2O_2 in the absence of a reducing substrate (like $DCFH_2$-MA) was not unexpected. In a similar experiment with native HRP in bulk PBS (0.5 μM), a substantial amount of the originally added H_2O_2 (9 μM) decomposed (≈ 40 % in 10 min) due to the "catalatic" activity of HRP, see Figure S-9 in the Electronic Supporting Information of Ghéczy et al. (2020),[S11] H_2O_2 being oxidizing as well as reducing substrate. According to the results from the bulk solution experiments (cascade reaction, stoichiometric H_2O_2 consumption, see **Figure 8D = Figure S30a**), the "catalatic" activity of HRP is largely suppressed in the flow-through cascade reaction (due to the intermediate formation of a sufficiently high concentration of the much better reducing substrate, $DCFH_2$-MA, than H_2O_2). In addition, a 10 min incubation of H_2O_2 with native BCA did not show any decrease in H_2O_2 concentration (see **Figure S37**). By exclusion principle, the observed decomposition of H_2O_2 seems to be caused by the denpol backbone of the conjugate (*de*-$PG2_{1000}$ or *de*-$PG2_{1000}$-HyNic). With still about four peripheral amino groups present per denpol r.u. and [r.u.] = 58 μM used in the conjugate incubation solution for immobilizing the denpol-BAH-BCA conjugate, 10 μM H_2O_2 were exposed to ≈ 200 μM amino groups for 10 min inside the BCA reactor. Whether the denpol backbone of the immobilized conjugates actually promotes the decomposition of H_2O_2 was not explored further.

Note that the observed H_2O_2 decomposition is only relevant for some of the conditions applied for running the cascade reaction in enzymatic flow-through reactor systems, namely for the low flow rate (5 μL min^{-1}, τ = 10 min) and the low H_2O_2 concentration of $[H_2O_2]_0$ = 10 μM. For $[H_2O_2]_0$

= 30 μM, and for the fast ABTS flow-through assay with excess H_2O_2 (200 μL min^{-1}, τ = 15 s, [ABTS^{2-}]$_0$ = 1.0 mM, [H_2O_2]$_0$ = 200 μM), the denpol-mediated decomposition of H_2O_2 was negligible. Consequently, pumping the ABTS assay solution (1.0 mM ABTS^{2-} and 200 μM, PBS) through the HRP reactor − with or without preceding BCA reactor − resulted in the same ABTS$^{\bullet-}$ concentrations in the reactor outflows. Moreover, when using 30 μM H_2O_2 in the cascade assay mixture (with [DCFH$_2$-DA]$_0$ = 50 μM), a longer BCA reactor through which the substrate solution passed first, did not change the oxidation turnovers (compare **Figure 10** to **Figure 9**).

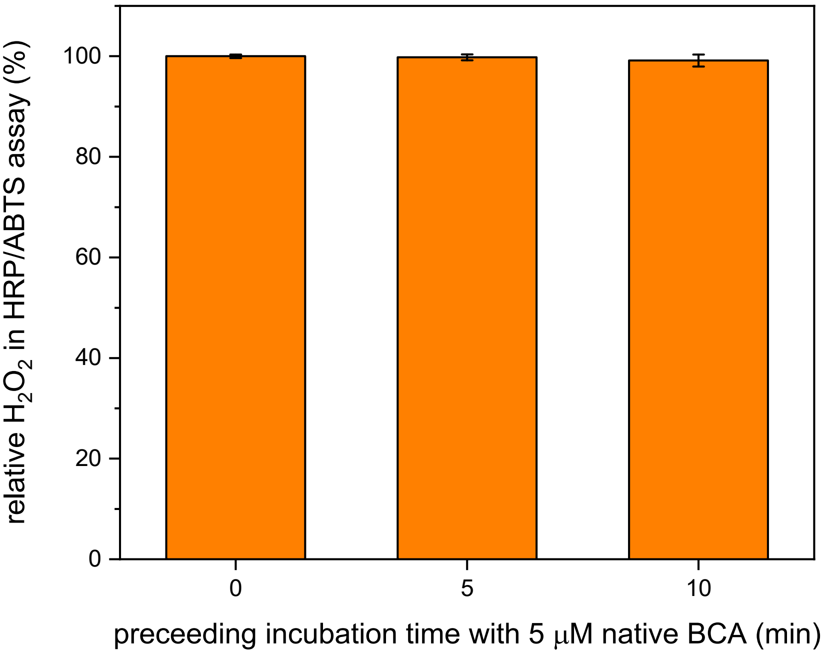

Figure S37. Stability of a 10 μM H_2O_2 solution in PBS (100 mM NaH_2PO_4, 150 mM NaCl, pH = 7.2) upon incubation with native BCA (5 μM) at RT. After mixing BCA and H_2O_2, excess amounts of HRP (0.5 μM) and ABTS^{2-} (1.0 mM) were added either immediately, or after 5 or 10 min of incubation. Upon addition of HRP/ABTS^{2-} the quickly stabilizing absorbance at λ = 414 nm, A_{414} (≈10 s) was taken as a relative measure of the amount of remaining [H_2O_2]. Apparently, incubation with native BCA had no effect on the stability of [H_2O_2] in PBS under the conditions used.

32. Quantification of DCF in the Cascade Reaction Outflow from Two Types of Reactor Systems − Consisting of either Sequentially or Co-immobilized BCA and HRP − Through Which a Solution of DCFH2-DA and H_2O_2 as Substrates was Pumped

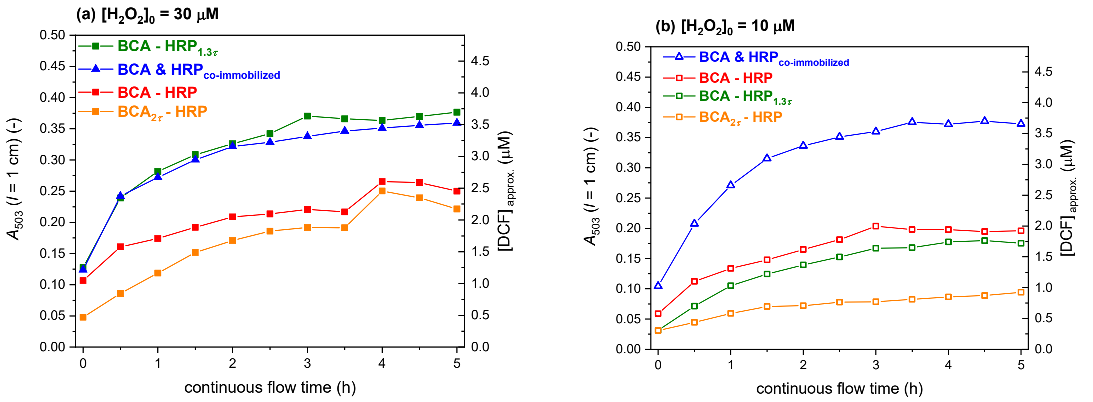

Figure S38. Four different enzyme reactor systems were used, as indicated (see also **Figure S34**). A substrate solution of DCFH2-DA (50 µM, 1 vol% DMSO) and H_2O_2 (30 µM **(a)** or 10 µM **(b)**) in PBS (100 mM NaH_2PO_4, 150 mM NaCl, pH = 7.2) was pumped through the enzyme reactor systems at 5 µL min^{-1}. The absorbance at λ = 503 nm, A_{503}, was measured for up to 5 h, and [DCF] was calculated by using ε_{503} (DCF, pH = 7.2) = 109 000 M^{-1}cm^{-1}, see Ghéczy et al. (2020).[S11] A_{460} stabilized after about 4 h of continuous flow. See **Figure S32** for the changes of the entire UV/vis spectra of the pooled outflows. The four enzyme reactor systems used are indicated, see also **Figure 2**, enzyme reactor systems (c) and (d).

For the preparation of the enzyme reactors from 50 µL solutions with defined concentrations of *de*-PG2$_{1000}$-BAH-BCA$_{89}$ and/or *de*-PG2$_{1000}$-BAH-HRP$_{40,}$ see Experimental Sections 2.6.3 and 2.6.4. In the case of the sequential setups, the enzyme-respective residence time (τ) was varied by varying the respective length of the monolith piece, using an accordingly higher volume of equally concentrated conjugate incubation solution (orange for BCA and green for HRP). The same amounts of the respective enzymes were either co-immobilized (blue) or sequentially immobilized (red) when using the monolith piece of standard length. In general, the enzyme reactor system consisting of co-immobilized enzymes had a higher molar ratio of DCF within the oxidized products (DCF + DCF-MA) since enzyme separation (sequentially immobilized) disallowed a BCA-catalyzed hydrolysis of DCF-MA (blocked BCA-catalyzed *Hyd_3* (see **Figure 3**), as already observed during our previous investigations, see Ghéczy et al. (2020)).[S11] See **Figure S39** for the molar ratio of DCF to (DCF + DCF-MA).

(a) $[H_2O_2]_0$ = 30 µM (*HRP rate limiting* for oxidation). The co-immobilized enzymes (blue) showed a higher DCF content in the outflow than the sequentially immobilized enzymes (red) due to the *increased molar ratio* of DCF to (DCF + DCF-MA). However, the main reason for the higher DCF content in the experiments with BCA-HRP$_{1.3\tau}$ (green data points) is the *increased yield of total oxidation products* (DCF-MA + DCF) due to a longer HRP-exposure of the substrate solution as compared to an enzyme reactor of standard length (compare with **Figure S34**).

(b) $[H_2O_2]_0$ = 10 µM (*H_2O_2 limiting for sequential immobilization*). When H_2O_2 was decreased from 30 µM to 10 µM, the product formation was not limited anymore by an oxidation rate that was linearly proportional to [HRP] and residence time but rather by H_2O_2 itself (at least for the sequential immobilization). The difference in DCF-formation between the enzyme reactor systems with co-immobilized enzymes and sequentially immobilized enzymes increased for $[H_2O_2]_0$ = 10 µM as compared to $[H_2O_2]_0$ = 30 µM because the co-immobilization performed better regarding the oxidation turnovers under H_2O_2-limited conditions while still yielding a higher molar ratio of DCF to (DCF + DCF-MA).

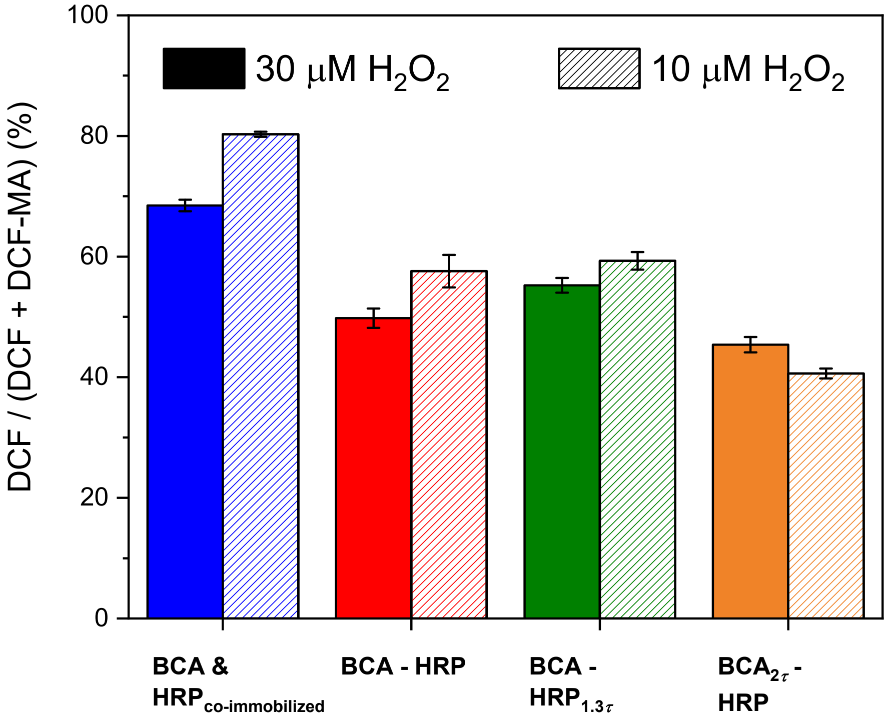

Figure S39. Determination of the molar ratio of DCF to (DCF + DCF-MA) for the four different enzyme reactor systems analyzed in **Figure S38**. For the cascade reaction proceeding along *pathway 2* (see **Figure 3**), the extent of hydrolysis of DCF-MA to DCF determines the distribution of the oxidzed products, *i.e.*, the molar ratio of DCF to (DCF + DCF-MA).

The data shown are based on an analysis of the outflow from the enzyme reactor systems between 4, 4.5 and 5 h, by taking into account A_{503} (see **Figure S38**) and A_{460} (see **Figure S34**). The DCF content was ≈ 20 % higher for the co-immobilized enzymes (blue) than for the sequentially immobilized enzymes (red) since enzyme separation in the case of the sequentially immobilized enzymes disallowed a BCA-catalyzed hydrolysis of DCF-MA to DCF (blocked BCA-catalyzed *Hyd_3*, as already observed by Ghéczy et al. (2020)).[S11] In both cases, the molar DCF to (DCF + DCF-MA) ratio was ≈ 10 or 20 % higher (for $[H_2O_2]_0$ = 30 or 10 μM) than in our previous work.[S11] This was most likely due to the significantly increased auto-hydrolysis of DCF-MA in the PBS buffer used in this work, as compared to our previous investigation using PB (see **Table S5**).

33. References

(S1) Childs, R. E.; Bardsley, W. G. The steady-state kinetics of peroxidase with 2,2′-azino-di-(3-ethyl-benzthiazoline-6-sulphonic acid) as chromogen. *Biochem. J.* **1975**, *145*, 93−103.

(S2) Dunford, H. B.; Stillman, J. S. On the function and mechanism of action of peroxidases. *Coord. Chem. Rev.* **1976**, *19*, 187−251.

(S3) Innocenti, A.; Scozzafava, A.; Parkkila, S.; Puccetti, L.; De Simone, G.; Supuran, C. T. Investigations of the esterase, phosphatase, and sulfatase activities of the cytosolic mammalian carbonic anhydrase isoforms I, II, and XIII with 4-nitrophenyl esters as substrates. *Bioorg. Med. Chem. Lett.* **2008**, *18*, 2267−2271.

(S4) Lindskog, S. Purification and properties of bovine erythrocyte carbonic anhydrase. *Biochim. Biophys. Acta* **1960**, *39*, 218−226.

(S5) Yoshimoto, M.; Schweizer, T.; Rathlef, M.; Pleij, T.; Walde, P. Immobilization of Carbonic Anhydrase in Glass Micropipettes and Glass Fiber Filters for Flow-Through Reactor Applications. *ACS Omega* **2018**, *3*, 10391−10405.

(S6) Fornera, S.; Balmer, T. E.; Zhang, B.; Schlüter, A. D.; Walde, P. Immobilization of Peroxidase on SiO_2 Surfaces with the Help of a Dendronized Polymer and the Avidin-Biotin System. *Macromol. Biosci.* **2011**, *11*, 1052−1067.

(S7) Hidaka, H.; Udenfriend, S. Evidence of a hydrazine-reactive group at the active site of the nonheme portion of horseradish peroxidase. *Arch. Biochem.Biophys.* **1970**, *140*, 174−180.

(S8) Grotzky, A.; Manaka, Y.; Kojima, T.; Walde, P. Preparation of Catalytically Active, Covalent α-Polylysine−Enzyme Conjugates via UV/Vis-Quantifiable Bis-aryl Hydrazone Bond Formation. *Biomacromolecules* **2011**, *12*, 134−144.

(S9) Goldberg, B.; Stern, A.; Peisach, J. The mechanism of superoxide anion generation by the interaction of phenylhydrazine with hemoglobin. *J. Biol. Chem.* **1976**, *251*, 3045−3051.

(S10) Hou, C.; Ghéczy, N.; Messmer, D.; Szymańska, K.; Adamcik, J.; Mezzenga, R.; Jarzębski, A. B.; Walde, P. Stable Immobilization of Enzymes in a Macro- and Mesoporous Silica Monolith. *ACS Omega* **2019**, *4*, 7795−7806.

(S11) Ghéczy, N.; Sasaki, K.; Yoshimoto, M.; Pour-Esmaeil, S.; Kröger, M.; Stano, P.; Walde, P. A two-enzyme cascade reaction consisting of two reaction pathways. Studies in bulk solution for understanding the performance of a flow-through device with immobilised enzymes. *RSC Adv.* **2020**, *10*, 18655−18676.

(S12) Küchler, A.; Messmer, D.; Schlüter, A. D.; Walde, P. *Methods Enzymol.* **2017**, *590*, 445−474.

(S13) Grotzky, A.; Altamura, E.; Adamcik, J.; Carrara, P.; Stano, P.; Mavelli, F.; Nauser, T.; Mezzenga, R.; Schlüter, A. D.; Walde, P. Structure and Enzymatic Properties of Molecular Dendronized Polymer–Enzyme Conjugates and Their Entrapment inside Giant Vesicles. *Langmuir* **2013**, *29*, 10831−10840.

(S14) Grotzky, A.; Nauser, T.; Erdogan, H.; Schlüter, A. D.; Walde, P. A Fluorescently Labeled Dendronized Polymer–Enzyme Conjugate Carrying Multiple Copies of Two Different Types of Active Enzymes. *J. Am. Chem. Soc.* **2012**, *134*, 11392−11395.

(S15) Reiniers, M. J.; van Golen, R. F.; Bonnet, S.; Broekgaarden, M.; van Gulik, T. M.; Egmond, M. R.; Heger, M. Preparation and Practical Applications of 2′,7′-Dichlorodihydrofluorescein in Redox Assays. *Anal. Chem.* **2017**, *89*, 3853−3857.
